# Regulation of mRNA poly(A) tail length governs mammalian body plan formation in gastruloids

**DOI:** 10.1038/s41467-026-76897-3

**Published:** 2026-08-29

**Authors:** David Taborsky, Fabiola Valdivia-Francia, Pablo Serrano, Sukalp Muzumdar, Neguin Ranjbar, Laura Llop-Grau, Guillermo Tellez, Pjeter Gjonlleshaj, Clara Duré, Peter F. Renz, Ramona Weber, Merve Yigit, Umesh Ghoshdastider, Aleksei Mironov, Katie Hyams, Stefano Vianello, Mihaela Zavolan, Ori Bar-Nur, Matthias P. Lutolf, Ataman Sendoel

**Affiliations:** 1 Institute for Regenerative Medicine (IREM), University of Zurich, Schlieren-Zurich, Switzerland; 2 Life Science Zurich Graduate School, Molecular Life Science Program, University of Zurich/ETH Zurich, Zurich, Switzerland; 3 Department of Biosciences, University of Milan, Milan, Italy; 4 ETH Zurich, Zurich, Switzerland; 5 Institute for Molecular and Translational Medicine, Department of Health Sciences and Technology, ETH Zurich, Schwerzenbach, Switzerland; 6 Biozentrum, University of Basel, Basel, Switzerland; 7 Institute of Bioengineering, École Polytechnique Fédérale de Lausanne, Lausanne, Switzerland; 8 Institute of Human Biology (IHB), Roche Pharma Research and Early Development, Roche Innovation Center, Basel, Switzerland; 9 Present Address: National Institute of Molecular Biology and Biotechnology, College of Science, University of the Philippines Diliman, Quezon City, Philippines

**Keywords:** Gastrulation, High-throughput screening, Gene expression analysis

## Abstract

The establishment of the body plan during gastrulation represents a hallmark of animal life. It emerges from the interplay of gene-regulatory programs and positional cues, yet how these signals are integrated post-transcriptionally remains largely unexplored. Here, we combine the scalability of mouse gastruloids with a single-cell CRISPR screening platform to functionally dissect germ layer specification at single-cell transcriptomic resolution. Focusing on post-transcriptional regulation, we systematically map drivers of mesodermal and endodermal fate and identify the deadenylase *Cnot8*. Loss of *Cnot8* leads to widespread poly(A) tail elongation and transcript stabilization, shifting mesoderm differentiation toward ectopic notochord fate, thereby profoundly impacting axial patterning. Collectively, our findings identify mRNA deadenylation as a fundamental mechanism linking cellular identity with morphogenetic signaling during mammalian body plan formation.

## Abstract (web summary)

How post-transcriptional mechanisms shape the mammalian body plan remains unclear. Here, the authors establish a single-cell CRISPR screening platform in gastruloids to identify Cnot8-dependent mRNA deadenylation as a driver of mesoderm patterning.

## Introduction

The formation of the embryo in vivo is an exquisitely complex process, integrating intrinsic and positional cues, coupled with dynamic cellular and tissue reorganization to establish the multidimensional body plan. Central to this process is gastrulation^1,2^, the event in which the multipotent and uncommitted epiblast gives rise to the three germ layers, ectoderm, mesoderm and endoderm. Gastrulation has been extensively studied using advanced imaging^3^ and sequencing tools^4,5^, providing insights into the transcriptional regulation of embryonic development, body axis formation and germ layer differentiation across species. Yet, how intrinsic and extrinsic signals are integrated at the post-transcriptional level remains largely unknown.

While these studies have greatly advanced our understanding of early embryogenesis, they have often remained observational, with functional perturbations largely limited to individual genes and requiring labor-intensive knockout embryos or cell-type-specific in vitro differentiation protocols^6,7^. Furthermore, although transcriptional regulation during early development has been extensively studied, post-transcriptional regulatory processes remain poorly defined, partly due to technical constraints and limited access to mammalian embryos. This gap underscores the need for scalable strategies to explore post-transcriptional regulation in a genome-wide manner to systematically define gene-regulatory networks during early development. By combining multiplexed perturbations with single-cell transcriptomic profiling, single-cell CRISPR screens offer a powerful strategy for uncovering regulators of embryonic processes^8,9^. Despite their promise, the application of these techniques to the embryo has seen limited use due to the technical challenges of delivering perturbation libraries to large numbers of accessible cells, highlighting the need for models that combine high cellular throughput with faithful recapitulation of embryonic cell diversity and morphogenesis.

Gastruloids can recapitulate key elements of mammalian embryogenesis in vitro^10–13^. Upon aggregation of mouse embryonic stem cells and exposure to a 24-h Wnt agonist pulse at 48 h of development^10,12,14,15^, gastruloids undergo essential steps of early embryonic development, including the emergence of the three germ layers^10,11,13–15^. Germ layer differentiation is accompanied by structural remodeling and gene expression changes^10–13,16^, including processes such as epithelial-to-mesenchymal transition and coordinated cell migration^16^.

Gastruloids capture crucial aspects of early body plan organization, including symmetry breaking and anterior-posterior axis formation, evidenced by spatially restricted expression of patterning factors such as *Hox* genes^11,12^. When cultured under specific conditions—including exposure to an extracellular matrix—gastruloids can undergo morphogenetic processes that give rise to complex structures, including somites^11,17^, neural tubes^17^, and cardiac precursors^18,19^. Consequently, gastruloids have enabled extensive profiling of developmental processes, spanning epigenetic^20^, transcriptomic^11–14,21^, metabolomic^22–25^ and proteomic^26^ landscapes.

Here, we couple gastruloids with a single-cell CRISPR screening strategy^8^ to establish a platform for the systematic identification of regulators of germ layer differentiation. Focusing on mRNA turnover and translational regulators, we uncover factors of germ layer specification in the mesodermal and endodermal lineages. We demonstrate that C*not8*, a deadenylase within the CCR4-NOT complex, orchestrates mammalian body-plan patterning via widespread control of mRNA decay. Our findings reveal a key developmental role of adenylation regulators, beyond the maternal-to-zygotic transition, demonstrating their involvement in governing body plan specification.

## Results

### Establishing a single-cell CRISPR platform in gastruloids

**Fig. 1:**
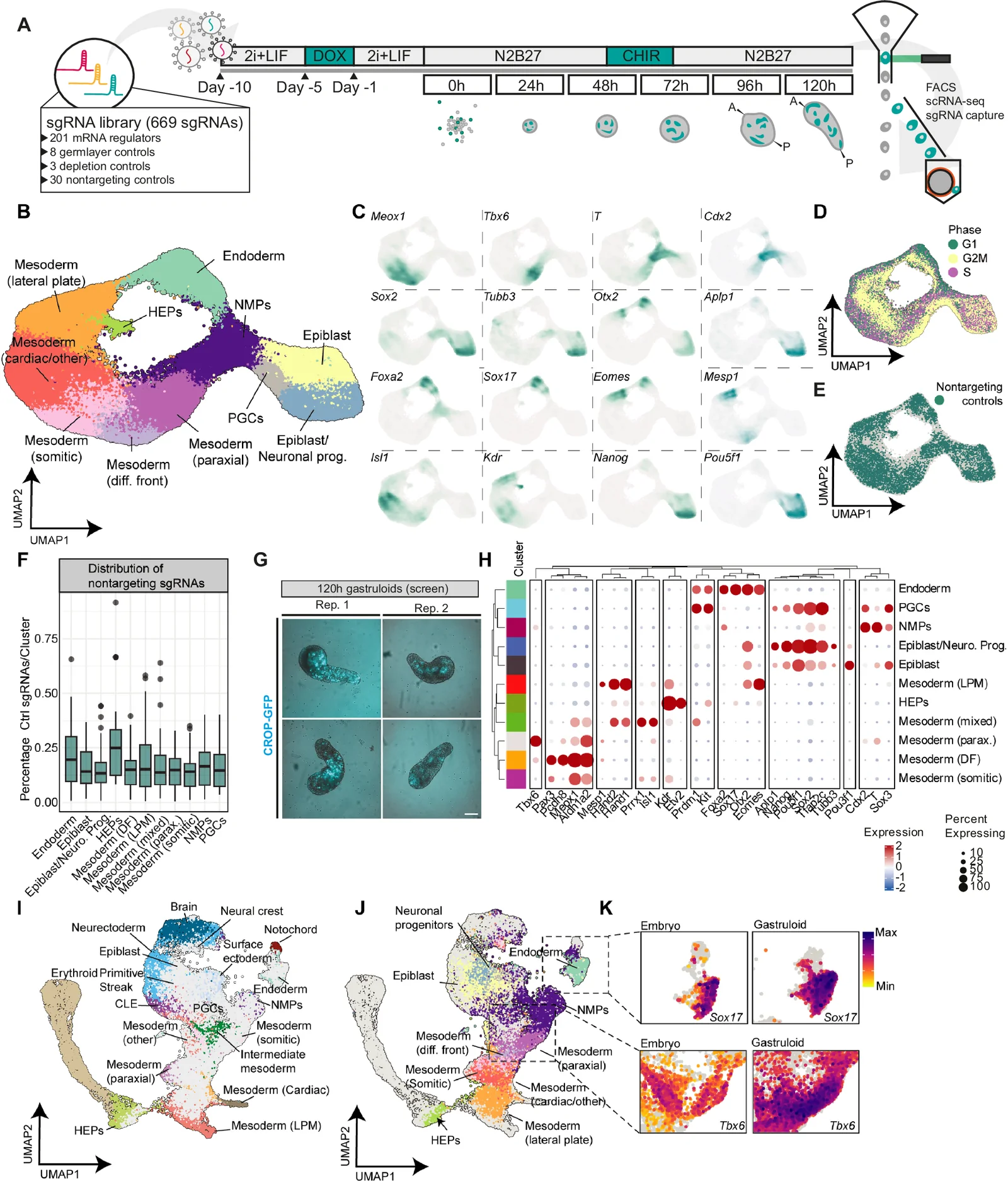
Establishing a single-cell CRISPR platform in gastruloids. **A** Schematic of the screen workflow. **B** UMAP embedding of 135,392 single cells from two replicate 120 h gastruloid CRISPR screens, identifying 11 distinct cell types after quality filtering and sgRNA assignment. **C** Marker gene expression profiles across major cell types were identified in 120 h gastruloids. Marker sets include mesodermal populations (*Meox1, Tbx6, Mesp1, Isl1*), neuromesodermal progenitors (*T, Sox2, Cdx2*), neuronal progenitors (*Tubb3, Sox2, Aplp1, Otx2*), endoderm (*Sox17, Foxa2, Eomes*), pluripotent epiblast (*Pou5f1, Nanog*) and early hematopoietic progenitors (*Kdr*). UMAP projections of 120 h gastruloids showing cell cycle states (**D**) and the uniform distribution of the 30 nontargeting control sgRNAs (**E**) across all cell types. **F** Box plot depicting the distribution of the 30 nontargeting control sgRNAs across all cell types. *n* = 30 nontargeting control sgRNAs (each point is one control guide’s percentage within a cluster). Box plots show the median (center line), the 25th and 75th percentiles (box bounds) and whiskers extending to the most extreme value within 1.5 times the interquartile range of the hinges; points beyond the whiskers are outliers. **G** Representative images of 120 h gastruloids on the screen. Scale bar, 200 µm. **H** Dot plot showing expression of cell-type-specific markers in the respective cell types. **I**, **J** UMAP projection of 135,392 cells from 120 h gastruloids mapped onto a reference atlas of mouse embryos spanning the developmental stages E7.5–E8.5. Gastruloid cells are shown in grey, with embryonic cell types highlighted (**I**). Embryonic cell types in grey, with gastruloid clusters highlighted, are shown in (**J**). **K** Feature plots showing the expression of endoderm marker *Sox17* and paraxial mesoderm marker *Tbx6* across gastruloid and mouse embryos.

**Fig. 2:**
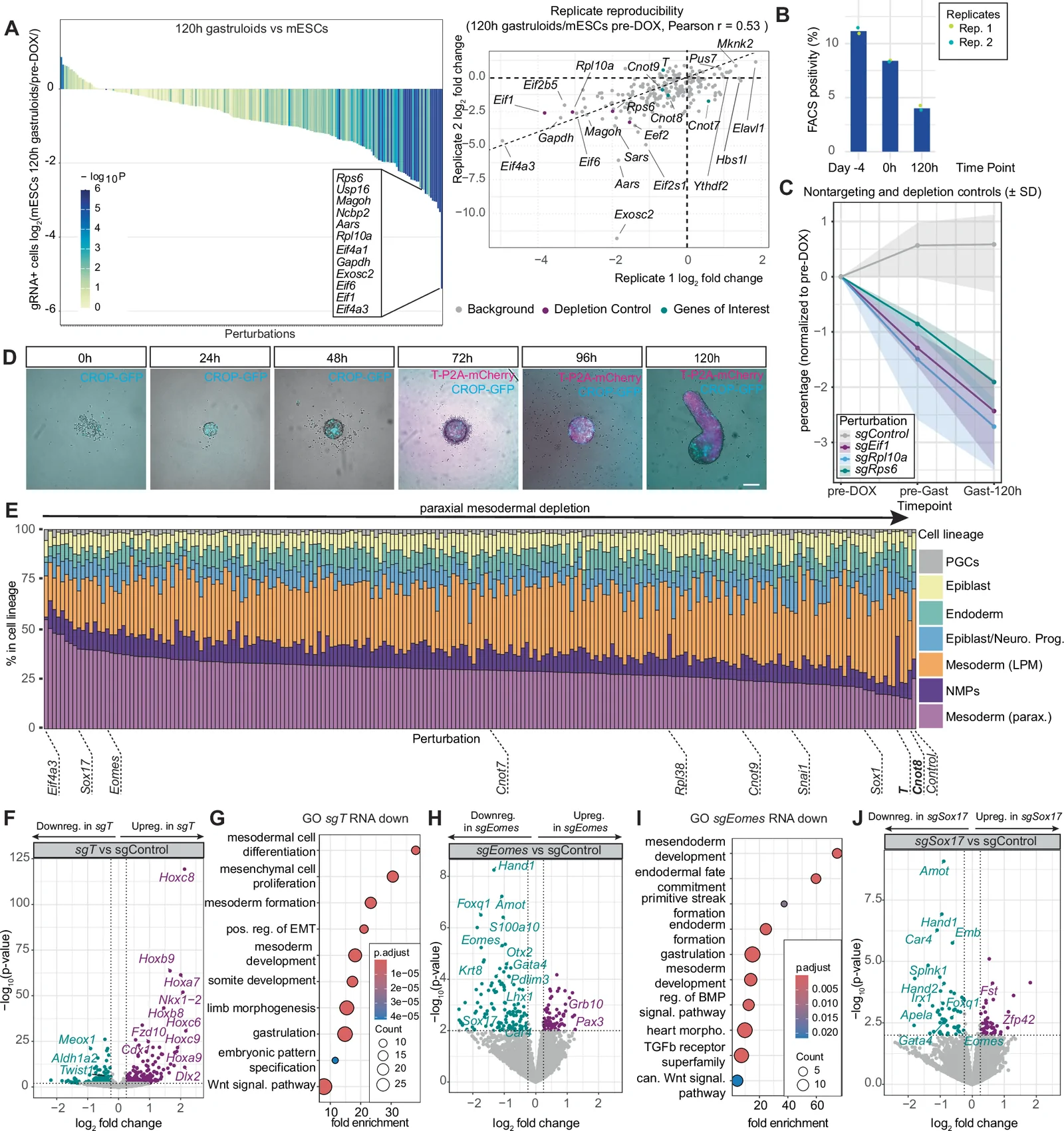
A single-cell CRISPR screen for identifying post-transcriptional regulators of germ layer differentiation in gastruloids. **A** Waterfall plot displaying log_2_ fold changes in 120 h gastruloid cell numbers relative to initial sgRNA representation in infected mouse embryonic stem cells (mESC) prior to doxycycline induction (left panel). Right panel, scatterplot showing gene-level reproducibility of enrichment and depletion between the two replicates. **B** Percentage of sgRNA-GFP positive cells across the different time points. **C** Stable propagation of the nontargeting control library. Mean abundance of 30 nontargeting control sgRNAs compared to the depletion of *Eif1, Rps6 and Rpl10a* (3 sgRNAs per gene) across 3 time points, pre-DOX induction (preDOX), pre-gastruloid aggregation (preGast) and after 120 h of gastruloid development (WTA). Data are presented as the mean (center), with the band representing the standard deviation (SD). **D** Time-lapse imaging of developing gastruloids from 0 h (mESCs) to 120 h, showing sgRNA-GFP expression and T-P2A-mCherry emerging from 72 h post-aggregation onward. **E** Cell type distribution plot of the 213 perturbations and nontargeting controls, sorted by perturbations most depleted in paraxial mesoderm. **F** Volcano plot displaying the top differentially expressed genes in 120 h gastruloids upon perturbation of *T* (*sgT*), pseudobulk differential expression by DESeq2 likelihood-ratio test with glmGamPoi dispersion, 2-sided, uncorrected *p* values (also **H** and **J**). **G** GO term enrichment of genes downregulated (*p* value < 0.01) in all *sgT* cells, clusterProfiler enrichGO (biological process, hypergeometric test, Benjamini-Hochberg adjusted). **H** Volcano plot displaying the top differentially expressed genes in 120 h gastruloids upon perturbation of *Eomes* (*sgEomes)*. **I** GO term enrichment of genes downregulated (*p* value < 0.01) in all *sgEomes* cells. ClusterProfiler enrichGO (biological process, hypergeometric test, Benjamini-Hochberg adjusted). **J** Volcano plot displaying the top differentially expressed genes in 120 h gastruloids upon perturbation of *Sox17* (*sgSox17*).

To systematically uncover post-transcriptional regulators of germ layer differentiation, we combined the CRISPR droplet sequencing (CROP-seq) strategy^8^ with gastruloids^10,15^. Starting from 406 genes associated with the gene ontology (GO) term “regulation of translation” (GO:0006417), we curated a list of 201 candidate genes based on their expression in gastruloids (Fig. S1A). In addition, we included 3 depletion controls and 8 known regulators of germ layer differentiation (herein referred to as germ layer controls). To target these genes, we designed a library containing 669 single guide RNAs (sgRNAs), with 3 sgRNAs per candidate gene, alongside sgRNAs for depletion controls, germ layer controls and 30 nontargeting control sgRNAs. We used a piggyBac-tetCas9 construct to introduce doxycycline-inducible Cas9 into a *Sox1/Brachyury* reporter (SBR) mouse embryonic stem cell (mESC) line proven to be well suited for gastruloid culture (Fig. S1B)^13,27^. The resulting tetCas9-SBR cells showed normal growth and elongation (Fig. S1C). We validated doxycycline-induced Cas9 expression by western blot and confirmed editing efficiency using sgRNAs targeting *Rpl11*, resulting in the expected depletion of infected cells (Fig. S1D–F). Following lentiviral production, the sgRNA library was used to infect tetCas9 mESCs on day −10 prior to the initiation of organoid culture. Cas9 expression was induced on day −5 for 72 h using doxycycline, and gastruloids were aggregated on day 0 (Fig. 1A). Gastruloids were harvested 120 h post-aggregation for single-cell RNA sequencing (scRNA-seq) coupled with sgRNA capture (Fig. 1A). We harvested ten 96-well plates of gastruloids from two independent cultures at 120 h post-aggregation and subjected them to single-cell RNA sequencing (scRNA-seq) with sgRNA capture. Following doublet removal, filtering, and sgRNA annotation, 135,392 cells were retained, corresponding to a median coverage of 201 cells per sgRNA. To prevent disruption of gastruloid development due to potential depletion of essential genes, we kept the proportion of sgRNA-positive cells low (8.3–8.5%) (Fig. 2B). Flow cytometry and time-lapse imaging revealed clearly separated clusters of GFP^+^ cells and a progressive overall depletion of the library by day 5 of gastruloid development (120 h post-aggregation), suggesting an effective depletion of essential translational regulators in the library (Fig. 2A–D). In contrast, control guides were evenly distributed across cell types (Figs. 1D–F and S2A, B) and showed stable propagation throughout gastruloid development until day 5 (Figs. 2C and S2D).

We identified cells belonging to all 3 germ layers in our dataset, as well as undifferentiated epiblast cells. Gastruloids showed an overall mesodermal bias with 81,710 cells (60% of total cells) belonging to various mesodermal lineages, including somitic (high in *Meox1*), paraxial (*Tbx6*) and lateral plate/cardiac mesoderm (*Hand1*, *Hand2*, *Isl1*) (Fig. 1B, C, H). Additionally, we identified an endodermal cluster with high expression of *Sox17* and *Foxa2* and an ectodermal cluster high in *Tubb3* and *Aplp1*, as well as neuromesodermal progenitors (NMPs) high in *Sox2*, *T/Brachyury* and *Cdx2* (Fig. 1B, C, H). Undifferentiated epiblast cells retained expression of the pluripotency markers *Nanog* and *Pou5f1* (Fig. 1B, C, H), in line with previous reports showing the persistence of undifferentiated epiblast throughout gastruloid development^13^. We noted the presence of neuronal markers *Tubb3*, *Aplp1* and Sox2 with pluripotency genes in one cluster, suggesting early neuronal fate mixed with pluripotent cells. We also observed a minor cluster high in the hematoendothelial progenitor markers *Kdr* and *Etv2* (Fig. 1B, C, H) and primordial germ cells (PGCs) high in *Tfap2c* and *Kit* (Fig. 1H). Integration and co-embedding of gastruloid cells with embryonic cell data from embryonic day (E) 7.5, E7.75, E8.0 and E8.5 embryos^4^ showed co-clustering of gastruloid cells with their assigned embryonic cell types, supporting the presence of cell types consistent with in vivo embryonic development (Fig. 1I–K).

**Fig. 3:**
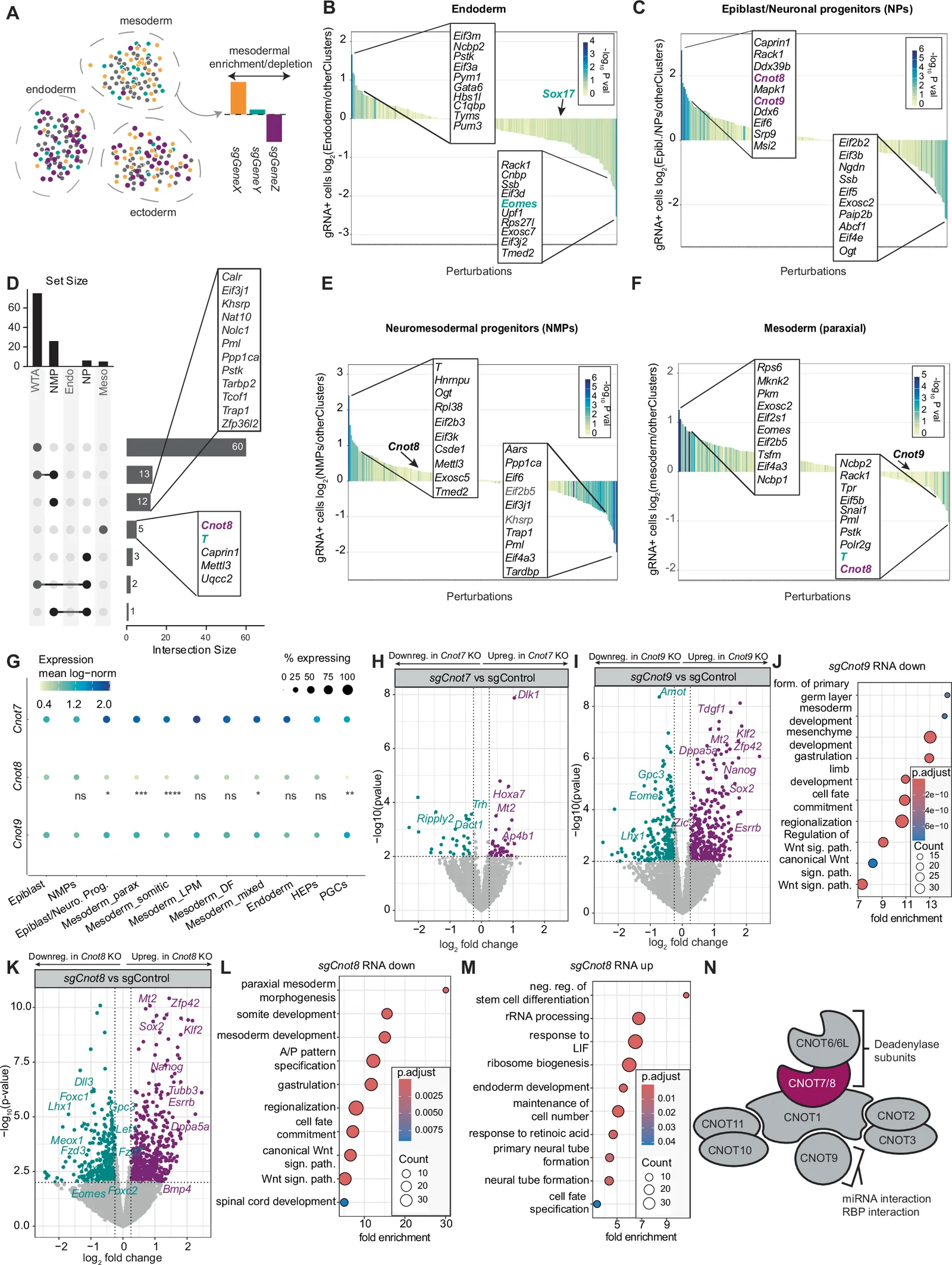
Uncovering novel post-transcriptional regulators of germ layer differentiation. **A** Schematic illustrating the strategy for identifying regulators of germ layer differentiation based on cell-type-specific sgRNA enrichment or depletion. Waterfall plots visualizing changes in sgRNA representation in endoderm (**B**) and neuronal progenitors (**C**). MAGeCK robust-rank aggregation normalized to the 30 nontargeting controls with permutation-based FDR computed separately for complementary enrichment and depletion one-sided tests (also for **E** and **F**). **D** UpSet plot displaying depleted targets across all cells (WTA), mesoderm (Meso), endoderm (Endo), epiblast (Epi), and neuronal progenitors (NPs). *Cnot8* and the germ layer control *T* are exclusively depleted in mesoderm (*p* value < 0.01, calculated using MAGeCK). **E** Waterfall plot showing sgRNA enrichment and depletion in neuromesodermal progenitors (NMP). **F** Waterfall plot showing sgRNA enrichment and depletion in paraxial mesodermal cells, with *Cnot8* emerging as the most significantly depleted perturbation in mesoderm. **G** Dot plot showing the expression of the CCR4-NOT components *Cnot7*, *Cnot8* and *Cnot9* across gastruloid cell types (control sgRNA cells). Percentage, % cells with >0 counts. *p* value, *p* adjusted from Wilcoxon rank-sum test comparing expression in cell type vs. epiblast (*p < 0.05, ****p < 10⁻⁴, ns *p* ≥ 0.05). **H**, **I** Volcano plot displaying the top differentially expressed genes in 120 h gastruloids upon perturbation of *Cnot7* (*sgCnot7*) and *Cnot9* (*sgCnot9*). DESeq2 likelihood-ratio test, two-sided, uncorrected *p* values (also for **K**). **J** GO term enrichment of genes downregulated (*p* value < 0.01) in all *sgCnot9* cells, showing enrichment for genes associated with Wnt signaling and developmental pathways. ClusterProfiler enrichGO (biological process, hypergeometric test, Benjamini-Hochberg adjusted) (also for **L** and **M**). **K** Volcano plot displaying the top differentially expressed genes in 120 h gastruloids upon perturbation of *Cnot8* (*sgCnot8*). **L** GO term enrichment of genes downregulated (*p* value < 0.01) in all *sgCnot8* cells, showing enrichment for genes associated with paraxial mesoderm development, anterior/posterior (A/P) pattern specification and Wnt signaling. **M** GO term enrichment of genes upregulated (*p* value < 0.01) in all *sgCnot8* cells, showing enrichment for genes associated with negative regulation of stem cell differentiation and neural tube formation. **N** Schematic of the CCR4-NOT complex with the paralogs CNOT7 and CNOT8 highlighted in magenta.

To validate our CRISPR screening strategy and Cas9 efficacy, we first examined known depletion controls. As expected, we found a strong and reproducible depletion of *Eif1*, *Rps6* or *Rpl10a* perturbed cells across gastruloid development (Fig. 2A, C). We then assessed cell-type-specific depletion phenotypes by comparing the distribution of perturbed cells across different cell types relative to the nontargeting control library (Figs. 2E and 3B–F). sgRNAs targeting the germ layer controls *Eomes* and *Sox17* showed endodermal depletion (Figs. 2E and 3B), while *T/Brachyury* and *Snai1* sgRNAs were depleted in mesodermal lineages (Fig. 2E), validating the high specificity of our screening strategy.

Unlike conventional CRISPR screens, the single-cell CRISPR approach also enables the evaluation of the transcriptomic changes of perturbed cells, providing insights into underlying mechanisms driving the observed phenotypes^8,28,29^. As validation, we first analyzed the transcriptomes of our germ layer controls (Figs. 2F–J and S2E–I). *T/Brachyury* is a key transcription factor during gastrulation^30–32^, particularly in mesodermal lineage commitment. As expected, its perturbation led to downregulation of mesodermal markers, including *Meox1* (Fig. 2F). This was further supported by GO term enrichment among downregulated genes, which highlighted processes related to gastrulation and mesodermal development (Fig. 2G). In line with these observations, depletion of *Eomes*, a transcription factor essential for endoderm induction^30,33,34^, showed a prominent downregulation of genes associated with endodermal development (*Gata4, Sox17, Krt8)* (Fig. 2H, I). *Sox17* perturbation similarly resulted in reduced expression of endodermal markers (*Eomes*, *Gata4, Krt8*) and markers of endocardial differentiation and heart development, including *Hand1* and *Irx1*, consistent with *Sox17’*s essential role in these processes (Figs. 2J and S2E)^35–37^. Furthermore, *Snai1* perturbed cells showed reduced expression of genes associated with mesodermal differentiation (*Mest*) and epithelial-to-mesenchymal transition^38,39^ (*Twist1*), while upregulating markers of neuronal lineages (*Sox2*) and undifferentiated cells (*Utf1*) (Fig. S2F, G).

### Loss of *Cnot8* results in mesodermal differentiation defects

To investigate germ layer-specific enrichment and depletion of sgRNAs, we used MAGeCK^40^ to calculate the significance of sgRNA alterations within individual germ layers compared to all other cell clusters (Tables S1 and S2). By focusing on germ layer-specific factors that were not globally depleted across gastruloids (Fig. 3D), we identified a set of strong candidates with putative roles in post-transcriptional control of germ layer differentiation (Figs. 3H–M and S3C–M). These included the RNA-binding protein *Msi2*, which showed marked depletion in the endoderm (Fig. 3D). Transcriptomic profiling reflected this phenotype, with downregulation of endodermal cell markers (*Sox17*, *Eomes*) as well as genes associated with mesoderm and heart morphogenesis (*Tbx6*, *Mesp1*) (Fig. S3D, E). Concomitantly, genes associated with pluripotency (*Dppa5a, Zfp42*) were upregulated, suggesting profound differentiation defects in *Msi2* perturbed cells. Another candidate was the Poly(A) binding protein interacting protein *Paip2b*, whose corresponding sgRNAs showed specific depletion in the NMP population (Fig. 3D, E). Transcriptomic analysis of *Paip2b*-perturbed cells supported this phenotype, revealing downregulation of NMP markers such as *Hoxc8* and upregulation of endodermal markers, including *Gata4* (Fig. S3F, G).

Intriguingly, CCR4-NOT complex components *Cnot8* and *Cnot9*^41–43^ showed a profound depletion of their corresponding sgRNAs in the mesoderm (Fig. 3F), with *Cnot8* being the top depleted candidate, while remaining unaltered in overall sgRNA representation and unaltered or even enriched within the endodermal, neuromesodermal and epiblast/neural compartments (Figs. 2A and 3B, C, E). This phenotype was more pronounced in paraxial mesodermal lineages compared to the lateral plate mesoderm and its derivatives, suggesting a role of *Cnot8* in this differentiation pathway (Figs. 2E and S3A, B).

*Cnot7* is a paralogue of *Cnot8*, with largely similar structure and function but higher binding affinity to the CCR4-NOT complex (Figs. 3N and S3N)^44^. Notably, our single-cell data suggested rather ubiquitous expression of *Cnot7* across all cell types, while *Cnot8* expression was lower in paraxial and somitic mesoderm compared to the epiblast population (Fig. 3G). As suggested by the lack of germ-layer-specific depletion of *Cnot7*, perturbation of *Cnot7* had little effect on the transcriptome compared to nontargeting controls (Fig. 3H). In contrast, perturbation of another CCR4-NOT component, *Cnot9*, led to strong transcriptomic changes, including downregulation of genes involved in Wnt signaling, gastrulation and differentiation, alongside upregulation of undifferentiated cell markers (Fig. 3I, J).

These transcriptomic changes were even more pronounced in *Cnot8* perturbed cells, with marked downregulation of genes associated with mesodermal differentiation (*Meox1*, *Eomes*, *Tbx6*), body patterning (*Dll1*) and Wnt signaling (*Lef1*) (Fig. 3K). In contrast, markers for neuronal differentiation (*Tubb3*) and pluripotent cell types (*Klf2*, *Nanog*) showed a robust upregulation (Fig. 3K–M). GO term enrichment analysis for genes depleted in *Cnot8* perturbed cells revealed an association with mesenchyme development, somite development, anterior-posterior (A/P) pattern specification, gastrulation and Wnt signaling (Fig. 3L). In contrast, genes upregulated in *Cnot8* perturbed cells revealed an enrichment for negative regulators of differentiation, endoderm development and neural tube and crest differentiation (Fig. 3M).

We observed a partial overlap between differentially expressed genes upon *Cnot8* and *Cnot9* perturbation, such as the upregulation of pluripotency factors *Zfp42*, *Klf2* and *Esrrb* (Figs. 3I, K and S3O). However, given its more pronounced paraxial mesodermal depletion (Fig. S3A) and transcriptomic impact (Fig. S3O), we subsequently focused on *Cnot8* and its function in gastruloid differentiation and patterning. Considering the central role of Wnt signaling in NMP differentiation^45,46^, promoting mesodermal fate upon activation and neuronal fate upon inhibition^47,48^, we surmised that the deregulation of Wnt signaling upon *Cnot8* perturbation may underlie the differentiation phenotypes observed in the screen. Despite their structural similarity (Fig. S3N), *Cnot7* and *Cnot8* exhibit distinct functional roles, as evidenced by the lack of mesodermal depletion upon *Cnot7* perturbation, mirroring the viability of *Cnot7* knockout mice compared to the gastrulation-stage lethality observed in *Cnot8* knockouts^49^. Other CCR4-NOT components included in the screen, such as *Cnot1* and *Cnot10*, exhibited strong overall depletion, consistent with their essential roles in the complex^41,50^, thereby precluding statistically significant germ layer–specific enrichment or depletion due to low cell numbers.

Consistent with previous reports^49^, *Cnot8*-perturbed cells showed upregulation in pluripotency markers, including *Klf2, Pou5f1* and *Nanog* (Fig. 3K–M). However, in contrast to earlier findings, our data indicated that *Cnot8*-perturbed cells retain the capacity to transition from naïve to formative and primed pluripotent states and can potentially acquire neuromesodermal, neuronal and endodermal fates, while being selectively depleted from the (paraxial) mesodermal lineage. These findings suggest a key role of *Cnot8*-mediated clearance of transcripts beyond the transition from naïve to formative pluripotency, aligning more closely with the in vivo phenotypes observed in *Cnot8* knockout embryos^49^. These embryos generally appear normal until E6.5, forming egg cylinders phenotypically similar to wild-type mice, but then fail during subsequent gastrulation between E7.5–8.5. Since these events take place after the naïve-to-formative transition^51^, the pronounced developmental defects upon *Cnot8* loss likely reflect disruptions in gastrulation-related signaling and mesoderm commitment.

### *Cnot8* knockout gastruloids show profound patterning defects with the induction of notochord cells

**Fig. 4:**
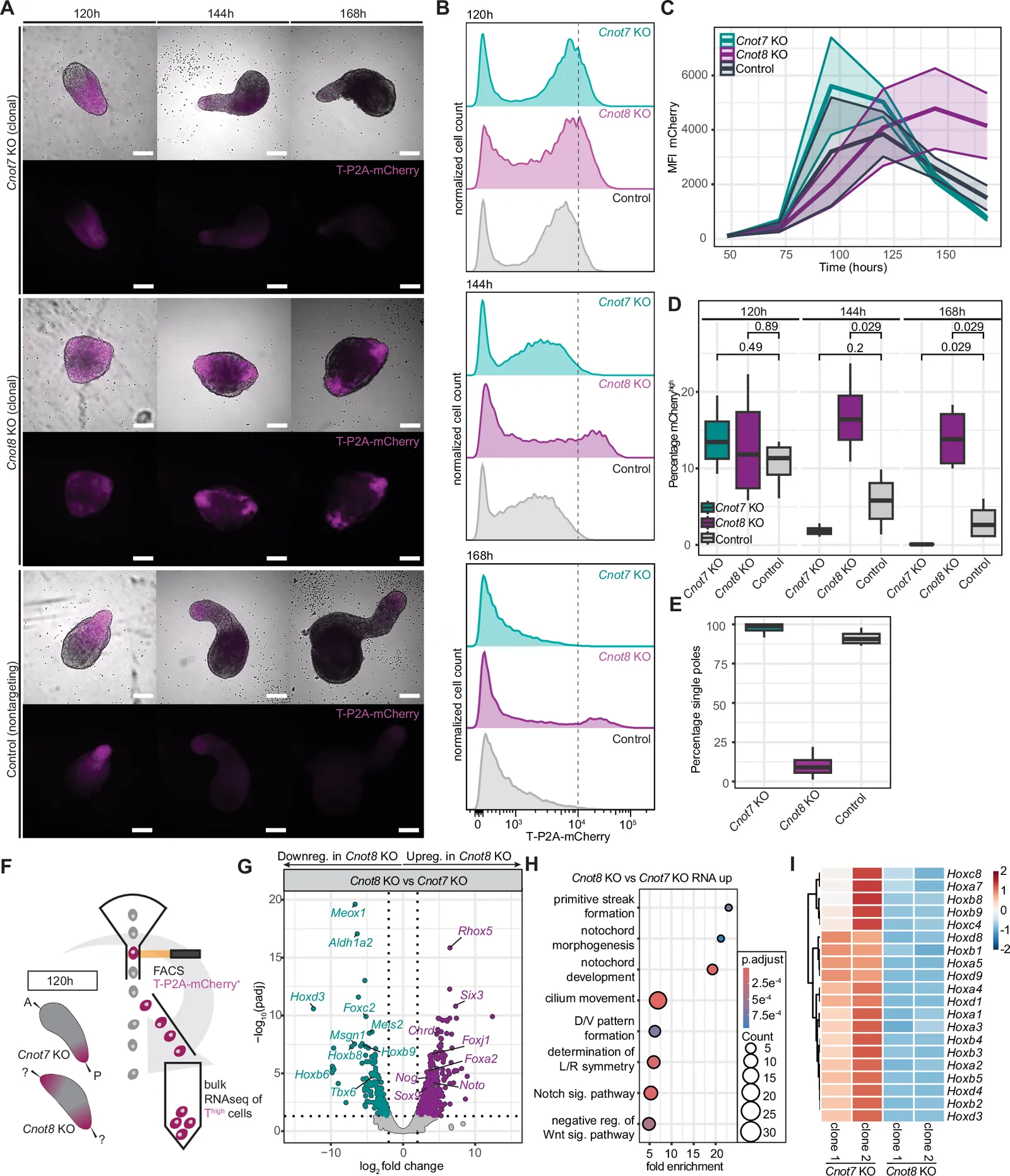
Loss of *Cnot8* results in gastruloid polarity disruption, higher T expression and appearance of notochord cells. **A** Time-lapse imaging of *Cnot8* knockout (KO), *Cnot7* KO and control (nontargeting sgRNA) gastruloids from 120 h (left), 144 h (middle) and 168 h (right), with T-P2A-mCherry expression in magenta. Scale bars, 200 µm.  **B** Flow cytometry of T-P2A-mCherry in *Cnot8* KO, *Cnot7* KO and control gastruloids from 120 h (top), 144 h (middle) and 168 h (bottom), showing the appearance of an mCherry^high^ population in *Cnot8* KO (dotted line). **C** Quantification of the mean fluorescence intensity for *Cnot8* KO, *Cnot7* KO and control gastruloids (*n* = 4). Error bands depict SD. **D** Quantification of the percentage mCherry^high^ cells as indicated in (**B**) (dotted line). Statistics were calculated using a two-sided Wilcoxon rank-sum test. *n* = 4 per genotype, comprising 2 independent clonal lines each measured in 2 independent replicate experiments (biological replicates); clones: *Cnot7* KO (c1, c2), *Cnot8* KO (c2, c4) and Control (CTRL2, CTRL11). Box plots show the median (center line), the 25th and 75th percentiles (box bounds) and whiskers extending to the most extreme value within 1.5 times the interquartile range; points beyond the whiskers are outliers. **E** Quantification of the number of gastruloids with single vs. multiple poles in 144 h *Cnot8* KO, *Cnot7* KO and control gastruloids (*n* = 4 replicates, 48 organoids per replicate/condition). Box plots show the median (center line), the 25th and 75th percentiles (box bounds) and whiskers extending to the most extreme value within 1.5 times the interquartile range; points beyond the whiskers are outliers. **F** Schematic of the sorting strategy used to isolate mCherry^high^ cells from 120 h gastruloids for bulk RNA-sequencing. **G** Volcano plot showing differentially expressed genes between *Cnot8* KO and *Cnot7* KO mCherry^high^ cells. DESeq2 Wald test, Benjamini-Hochberg corrected *p* values. **H** GO term enrichment analysis of genes upregulated in *Cnot8* KO vs. *Cnot7* KO mCherry^high^ cells. ClusterProfiler enrichGO (biological process, hypergeometric test, Benjamini-Hochberg adjusted). **I** Heatmap showing expression levels of *Hox* genes in *Cnot8* KO vs. *Cnot7* KO mCherry^high^ cells from two different cell lines per condition.

To validate the differentiation defects observed in *Cnot8* perturbed cells, we next generated *Cnot8* and *Cnot7* clonal knockout cell lines. We verified the knockout alleles in the clonal cell lines by employing the sequencing-based TIDE assay^52^ (Fig. S4A–H) and evaluated their capacity to form gastruloids (Figs. 4A and S4I). Consistent with the results of the single-cell CRISPR screen, while *Cnot7* knockout gastruloids developed normally, *Cnot8* knockout gastruloids showed profound phenotypes (Figs. 4A and S4I). Using the *T*/*Brachyury*-P2A-mCherry reporter system in the SBR cell line, we observed that *Cnot8* knockouts failed to generate a T/Brachyury-positive posterior pole and exhibited impaired elongation (Fig. 4A). Instead, *T/Brachyury* expression was initially broad at 120 h and later coalesced into multiple poles (Fig. 4A, E). While *T/Brachyury* expression decreased over time in both control and *Cnot7* knockout organoids due to NMP depletion, *Cnot8* knockout organoids maintained a robust *T/ Brachyury*-P2A-mCherry^high^ population beyond 144 h of development, confirmed by both time-lapse imaging (Fig. 4A) and flow cytometric analysis (Fig. 4B–D).

To further investigate this phenotype, we isolated the *T-P2A-mCherry*^*high*^ populations from 120 h *Cnot7* and *Cnot8* knockout gastruloids for transcriptomic profiling (Fig. 4F and Table S3). Differential expression analysis of *Cnot8* knockouts revealed a strong enrichment for GO terms associated with notochord development, including canonical notochord markers like *Foxa2*, *Noto* and *Chrd*, along with genes associated with cilium movement (Fig. 4G, H). These data suggest that the *T/Brachyury*-P2A-mCherry^high^ cells observed in *Cnot8* knockout gastruloids represent notochord cells and not NMPs. Additionally, markers of other mesodermal lineages, especially somitic and paraxial mesoderm markers like *Meox1* and *Tbx6*, were drastically downregulated in *Cnot8* knockouts (Fig. 4G), alongside an array of *Hox* genes (Fig. 4I), suggesting a loss of anterior-posterior polarity in the gastruloids^11,12^. This phenotype was particularly notable as mouse gastruloids typically do not generate notochord cells without modified culture conditions or additional signaling cues^19,53^.

The notochord is a defining patterning hub in chordate embryos, essential for the formation of the dorsal-ventral body axis and the differentiation of adjacent tissues, including the neural tube, endoderm and paraxial mesoderm^53^. It is derived from the anterior primitive streak, which gives rise to both axial and paraxial mesoderm^48^. While the precise mechanisms of notochord formation remain incompletely understood, modulation of Nodal/Activin and more broadly TGFβ signaling^53,54^ is required for in vitro generation of notochord-like cells. Thus, the formation of notochord cells in *Cnot8* knockout gastruloids points to a substantial signaling landscape shift toward a state permissive for notochord differentiation, ultimately leading to the emergence of a secondary signaling center capable of producing key morphogens such as sonic hedgehog (*Shh)* and bone morphogenetic protein (BMP) inhibitors, including *Chrd* and *Nog*.

### Loss of *Cnot8* induces notochord formation and reprograms axial patterning

**Fig. 5:**
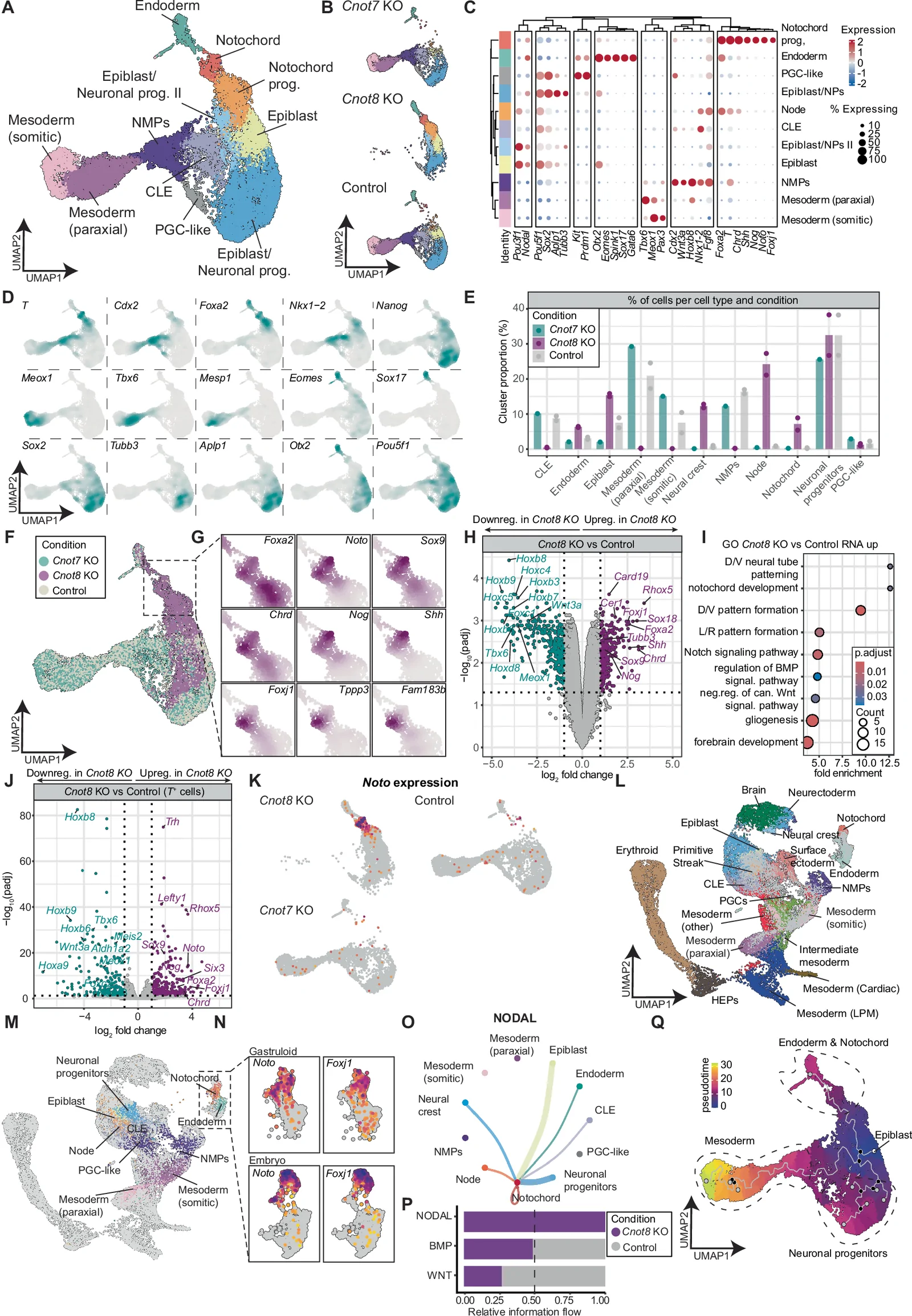
*Cnot8* KO single-cell RNA sequencing reveals altered differentiation trajectories. **A** UMAP of 16,966 cells from *Cnot8* KO, *Cnot7* KO and control (nontargeting sgRNA) gastruloids at 120 h post-aggregation. **B** Individual UMAPs of *Cnot7* KO (top), *Cnot8* KO (middle) and control (bottom) gastruloids. **C** Dot plot showing marker gene expression across the identified cell types. **D** Nebulosa density plots showing cell-type markers for neuromesodermal progenitors (NMPs) (*T*, *Cdx2, Nkx1-2*), mesoderm (*Meox1, Tbx6, Mesp1*), endoderm (*Sox17, Eomes, Otx2, Foxa2*), neuronal progenitors (NPs) (*Tubb3, Aplp1, Sox2, Otx2*), epiblast (*Pou5f1, Nanog*), node-like and caudal lateral epiblast (CLE) (*Nkx1-2*) and notochord (*Foxa2*). **E** Bar plot quantifying cell type composition in *Cnot7* KO, *Cnot8* KO and control gastruloids. **F** UMAP highlighting *Cnot8* KO enrichment in notochord and anterior primitive streak/node-like regions. **G** Nebulosa density plots showing the expression of canonical notochord markers (*Foxa2*, *Noto*, *Sox9*, *Chrd*, *Nog*, *Shh*, *Foxj1*, *Tppp3* and *Fam183b*). **H** Volcano plot displaying the top differentially expressed genes in *Cnot8* knockout vs. control gastruloids across all cell types. DESeq2 likelihood-ratio test with glmGamPoi dispersion, two-sided with Benjamini-Hochberg correction (also for **J**). **I** GO term enrichment of genes downregulated (*p* adj <0.05, log_2_ fold change < −1) in all *Cnot8* knockout cells. ClusterProfiler enrichGO (biological process, hypergeometric test, Benjamini-Hochberg adjusted). **J** Volcano plot showing the top differentially expressed genes in *Cnot8* knockout vs. control *T*-positive cells. **K** Feature plot showing *Noto* expression in *Cnot7* KO, *Cnot8* KO and control gastruloids. **L**, **M** UMAP showing the mapping of gastruloid cells to a reference embryo atlas (E7.5–E8.5). **L** UMAP showing embryonic cell clusters with gastruloid cells in grey, **M** UMAP showing gastruloid cell clusters with embryonic cells in grey. **N** Feature plot of *Noto* and *Foxj1* expression in gastruloids (top) and embryonic (bottom) cells. **O** CellChat analysis of *Nodal* signaling in *Cnot8* KO gastruloids, showing high activity between notochord and other cell types. **P** Relative information flow of *Nodal*, *Bmp* and *Wnt* signaling between *Cnot8* KO and control gastruloids. **Q** Pseudotime trajectory analysis comparing differentiation dynamics in *Cnot7* KO, *Cnot8* KO and control organoids.

We sought to characterize the overall changes in cell type composition in *Cnot8* knockout, *Cnot7* knockout and control organoids using scRNA-seq (Table S4). We derived *Cnot8* knockout and control gastruloids from two different clonal knockout cell lines harboring different sgRNAs, complemented with gastruloids from a single *Cnot7* knockout clonal cell line. Organoids showed comparable size, cell viability, and appeared healthy, while recapitulating the described *Cnot8* knockout phenotype. For each condition, we harvested 16 gastruloids, dissociated the organoids and pooled single cells in equal ratios and subjected to scRNA-seq. After removal of doublets, low-quality and dead cells, 16,966 cells were retained for analysis, consisting of 4873 *Cnot7* knockout, 4858 *Cnot8* knockout and 7235 control cells. Unbiased clustering allowed for the identification of 11 clusters, including mesodermal cells, neuromesodermal progenitors (NMPs), a mixed population containing epiblast and neuronal progenitors, epiblast, endoderm as well as an unexpectedly high number of notochord cells (Fig. 5A). Marker gene expression was consistent with these cell type assignments, including *Meox1* and *Tbx6* for mesoderm, *Tubb3* and *Otx2* for neuronal progenitors*, T/Brachyury* and *Cdx2* for NMPs, *Sox17* and *Gata6* for endoderm and *Foxa2*, *Noto* and *Chrd* for notochord (Fig. 5A, C, D).

The emergence of a *Foxa2*, *Chrd* and *Noto*-expressing cluster resembling notochord was particularly striking, as prior mouse gastruloid datasets^11–13^ suggested a complete absence of this cell type. Additionally, notochord differentiation in a human model of trunk development has only recently been achieved under highly specialized culture conditions^53,55^. Closer examination of cell line-specific contributions revealed a profound enrichment of *Cnot8* knockout cells in this notochord cluster, alongside a marked depletion from all other mesodermal cell types and NMPs (Figs. 5B, E, F and S5A). In addition, *Cnot8* knockout cells were also overrepresented in undifferentiated epiblast in line with previous reports that *Cnot8* loss interferes with pluripotency exit^49^. In contrast, endoderm differentiation was preserved or mildly enhanced compared to *Cnot7* knockout and control gastruloids (Fig. 5E). *Cnot7* knockout organoids displayed differentiation profiles similar to controls, supporting their utility as a second control condition (Figs. 5E and S5A). Differential expression analysis between *Cnot8* knockout cells and control cells echoed the observed cell type changes, with downregulation of paraxial mesoderm markers *Tbx6* and *Meox1* and upregulation of neuronal (*Tubb3*) and notochord (*Foxa2*, *Sox9*, *Shh*, *Nog*, *Chrd*) markers (Figs. 5H, I and S5D). This coincided with a loss of *Hox* genes (Figs. 5H and S5C). Additionally, consistent with our previous observations in *T-P2A-mCherry*^*high*^ populations (Fig. 4G), T-positive *Cnot8* knockout cells displayed increased expression of notochord markers such as *Foxa2, Noto* and *Chrd*, accompanied by a reduction in *Hox* gene expression (Fig. 5J). Focusing on progenitor populations, we combined epiblast, caudo-lateral epiblast (CLE, enriched in *Cnot7* knockout and nontargeting controls) and node-like clusters (enriched in *Cnot8* knockout), which collectively revealed upregulation of genes involved in neural, notochord and primitive streak differentiation as well as dorso-ventral patterning. In contrast, genes associated with anterior-posterior patterning and mesoderm formation were downregulated (Fig. S5E–H). The upregulation of neuronal markers, including *Tubb3*, could be indicative of increased maturity of the neuronal progenitor cells found in the organoids, even though the overall immature state of these cells limits our ability to make definitive conclusions regarding their differentiation.

Further analysis of the putative notochord cluster revealed the presence of notochord-associated signaling factors, including BMP inhibitors *Chrd*, *Nog*, the hedgehog ligand *Shh*, the notochord marker *Noto*, as well as the cilia-related genes *Tppp3* and *Fam183b* (Fig. 5F, G, K). Integration of these cells with embryonic data from E7.5-E8.5 embryos^4^ revealed co-clustering of gastruloid and embryo notochord, with largely overlapping markers (Fig. 5L–N), consistent with the presence of bona fide notochord cells in *Cnot8* knockout gastruloids.

Given the known function of the notochord as a signaling hub in the developing embryo, especially as a source of BMP inhibitors and *Shh*, we compared the signaling landscape of *Cnot8* knockout organoids to *Cnot7* knockout and control. CellChat^56^ analysis of control cells confirmed the role of NMPs and CLE as a central source of Wnt signaling, particularly of *Wnt3a* (Fig. S5I). The depletion of NMPs in *Cnot8* knockouts results in a loss of *Wnt3a* across the organoid. This was also reflected in the downregulation of Wnt target genes, such as *Axin2*, in *Cnot8* knockout organoids and the loss of anterior-posterior patterning markers, including *Hox* genes, which are typically expressed at this developmental stage^11,12^. Using CellChat, significant Nodal signaling could only be detected in the *Cnot8* knockout organoids, where epiblast, neural crest, endoderm and the notochord itself provided Nodal signals for the notochord (Fig. 5O, P). Pseudotime analysis of the combined dataset revealed three independent differentiation pathways starting in the epiblast, with both cell types forming neuronal lineages, but *Cnot8* knockout organoids taking a strikingly altered mesodermal differentiation path, favoring axial over paraxial mesoderm development (Fig. 5Q).

### CNOT8 regulates transcript poly(A) tail length and transcript abundance

**Fig. 6:**
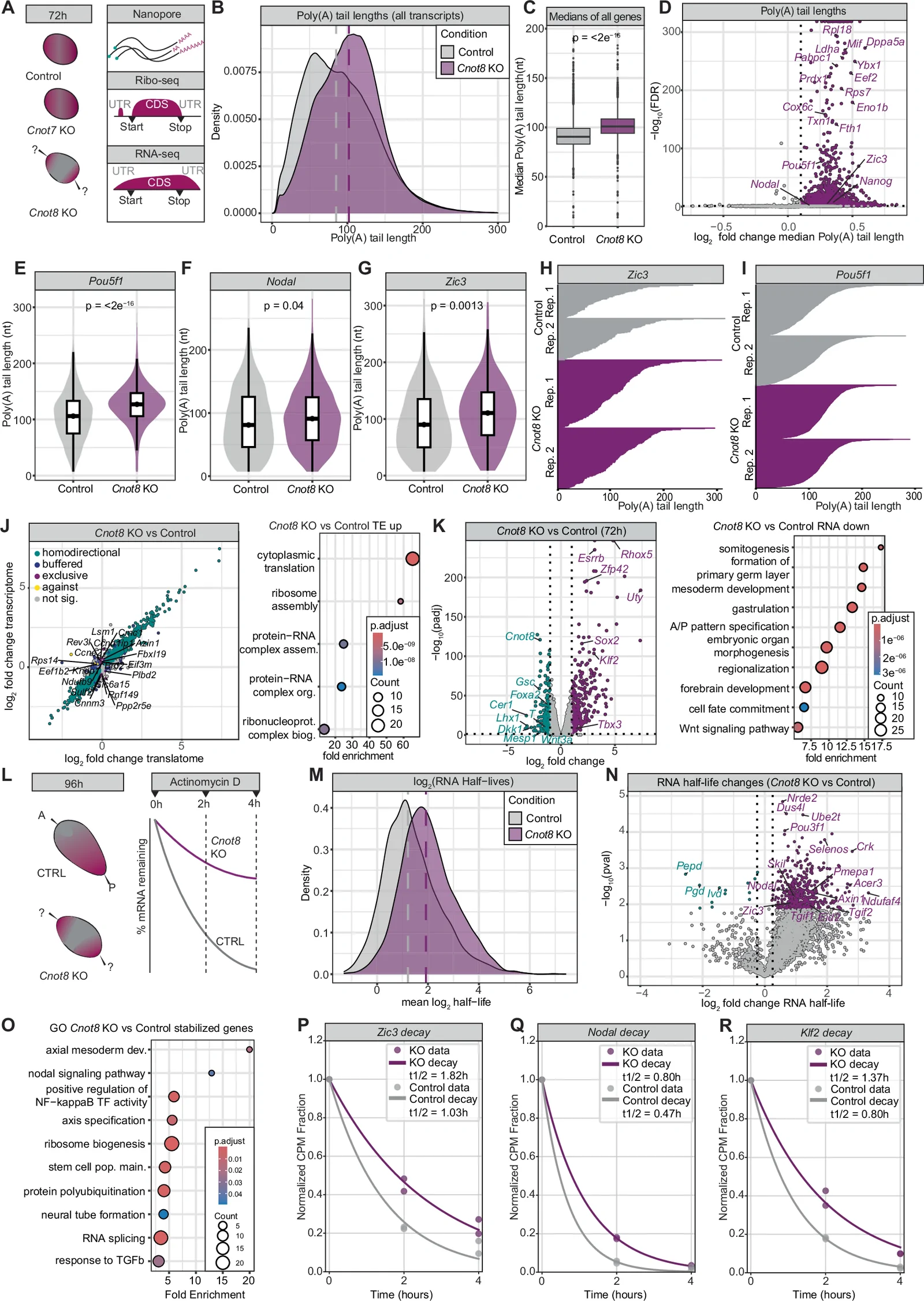
Loss of *Cnot8* results in poly(A) tail elongation and transcript stabilization. **A** Experimental outline to dissect *Cnot8* function. Gastruloids from *Cnot8* KO, *Cnot7* KO and control organoids were harvested at 72 h and analyzed by ribosome profiling, bulk RNA-sequencing, and Nanopore direct RNA-sequencing to quantify poly(A) length. **B** Density plot showing poly(A) tail lengths across all transcripts in *Cnot8* KO and control 72 h gastruloids. **C** Box plot showing the median poly(A) tail lengths in *Cnot8* KO and control gastruloids. *n* = number of genes passing filter (each point is one gene’s median poly(A) length), Control versus *Cnot8* KO. Box plots show the median (center line), the 25th and 75th percentiles (box bounds) and whiskers extending to the most extreme value within 1.5 times the interquartile range; points beyond the whiskers are outliers. Statistical significance was assessed by a two-sided Wilcoxon rank-sum test. **D** Volcano plot showing the log_2_ fold change in poly(A) tail length upon *Cnot8* knockout. *p* values indicate a Wilcoxon test with multiple testing correction using the Benjamini-Hochberg method. Box plot showing poly(A) tail lengths of *Pou5f1* (**E**), *Nodal* (**F**) and *Zic3* (**G**). The unit is the individual Nanopore read, pooled from 2 control and 2 *Cnot8* KO libraries; read counts after the qs > 10 and mapq > 10 filter (control versus *Cnot8* KO) are *Pou5f1* *n* = 2566/2630 (**E**), *Nodal* *n* = 599/776 (**F**) and *Zic3* *n* = 175/302 (**G**). Box plots show the median (center line), the 25th and 75th percentiles (box bounds) and whiskers extending to the most extreme value within 1.5 times the interquartile range; points beyond the whiskers are outliers. Statistical significance was assessed by a two-sided Wilcoxon rank-sum test on per-read poly(A) lengths (Control versus *Cnot8* KO). Plot depicting the poly(A) tail lengths of *Zic3* (**H**) and *Pou5f1* (**I**). **J** Scatter plot showing the log_2_ fold changes in transcript abundance and ribosome occupancy (translatome) of 72 h *Cnot8* KO versus control organoids. Genes regulated exclusively at the translational level are shown in magenta. DESeq2 likelihood-ratio test on the Condition x SeqType interaction, *p*adj <0.05, Benjamini-Hochberg. Right panel: GO terms enriched among genes with increased translational efficiency (TE). **K** Volcano plot showing differentially expressed genes (transcriptome) in *Cnot8* KO versus control. DESeq2 Wald test, Benjamini-Hochberg corrected *p* values. Right panel: GO term enrichment of transcriptionally downregulated genes in *Cnot8* KO versus control at 72 h. ClusterProfiler enrichGO (biological process, hypergeometric test, Benjamini-Hochberg adjusted). **L** Schematic of RNA decay experiment. 96 h gastruloids were treated with Actinomycin D and RNA was collected at 0 h, 2 h and 4 h post-treatment. **M** Density plot comparing RNA half-lives for *Cnot8* KO and control organoids. **N** Volcano plot highlighting genes with altered RNA half-lives in *Cnot8* KO versus control organoids. **O** GO term enrichment of stabilized genes in *Cnot8* KO, suggesting an enrichment for genes associated with axial mesoderm development, Nodal signaling, NF-κB activity and ribosome biogenesis. ClusterProfiler enrichGO (biological process, hypergeometric test, Benjamini-Hochberg adjusted). Transcript decay plots of stabilized genes in *Cnot8* KO, including *Zic3* (**P**), *Nodal* (**Q**) and *Klf2* (**R**).

Having confirmed the *Cnot8* perturbation phenotype from the initial screen, we next investigated the molecular mechanisms underlying the observed developmental changes. Given the canonical function of CNOT8 as a deadenylase subunit of the CCR4-NOT complex (Fig. 3N)^49,57^, we examined how its loss impacts the polyadenylation landscape of gastruloids. To accurately quantify changes in poly(A) tail length upon *Cnot8* loss, we performed Nanopore direct RNA sequencing on 72 h gastruloids from *Cnot8* knockout and control conditions (Fig. 6A). As expected, *Cnot8* knockout gastruloids showed a marked global increase in median poly(A) tail length (Fig. 6B) across all transcripts (Fig. 6C, D). Mitochondrial transcripts exhibited no increase in poly(A) tail length, consistent with the known cytoplasmic and nuclear restriction of the CCR4-NOT complex (Fig. S7B)^57^. To identify functionally relevant targets of CNOT8-mediated deadenylation, we assessed candidate transcripts with poly(A) tail changes in *Cnot8* knockouts. Consistent with previous findings^49^, *Pou5f1*, *Nanog* and *Dppa5a* showed increased poly(A) tail lengths (Figs. 6E, I and S7C, D), supporting a role for CNOT8 in modulating pluripotency. Interestingly, *Zic3* and *Nodal* also showed increased poly(A) tail length in *Cnot8* knockout (Fig. 6F–H). *Nodal*, a TGFβ family member, plays an essential role in patterning the early embryo and promoting primitive streak formation^58,59^. *Zic3* has been implicated in the formation of axial mesoderm/notochord in other chordates like *Xenopus laevis*^60^, making both genes promising candidate mediators of the *Cnot8* knockout phenotype.

Deadenylation plays a central role in reducing transcript stability and, in early development, additionally mediates translational repression^61,62^. To distinguish between these two outcomes, we first set out to establish a translational landscape of wild-type gastruloids. We performed ribosome profiling from gastruloids across 4 developmental stages, including 48, 72, 96, and 120 h post-aggregation. The resulting dataset showed hallmarks of high-quality ribosome profiling datasets, including triplet periodicity, P-site distribution biased toward coding sequence (CDS) and an enrichment of 30-nucleotide footprint sizes (Fig. S6A–C). To assess translational control during development, we examined changes in translational efficiency (TE, defined as ribosome profiling reads divided by RNA sequencing reads) throughout organoid development (Table S5). Comparing 72 h to 48 h post-aggregation, corresponding to early gastrulation, we observed translational regulation of the translational machinery, including various components of the ribosome and eukaryotic initiation factors (Fig. S6D–F). Components of the Wnt signaling pathway also showed altered translational efficiency, consistent with translational buffering of this key developmental pathway in gastrulation (Fig. S6E, F). GO term analysis highlighted enrichment of Wnt signaling among genes with reduced translational efficiency (Fig. S6F). At later stages, fewer TE changes were detected, which may reflect the limitations of bulk ribosome profiling in resolving translational differences across diverse cell types of late-stage gastruloids (Fig. S6G, H).

Having established the translational landscape of gastruloids, we investigated translational changes in *Cnot8* knockouts. We subjected 72 h *Cnot8* knockout, *Cnot7* knockout and control gastruloids to ribosome profiling (Table S6). *Cnot8* knockout gastruloids displayed widespread transcriptional changes, with 5451 genes showing differential expression compared to controls (Figs. 6J, K and S6I–L). Downregulated genes were enriched for terms related to gastrulation, differentiation and Wnt signaling, while pluripotency-associated genes such as *Sox2*, *Esrrb*, and *Klf2* were strongly upregulated (Fig. 6K). However, when contrasting transcriptomic and translatomic changes, we observed minimal alterations in translational efficiency (Fig. 6J), suggesting that *Cnot8* primarily impacts transcript stability rather than translation. In line with the lack of phenotype observed in *Cnot7* knockout organoids, we found minimal transcriptional and translational changes in *Cnot7* knockouts (Fig. S6L). Altogether, our integrated 72 h dataset demonstrates that *Cnot8* knockout leads to extensive remodeling of the transcriptome and poly(A) tail landscape, while having a limited impact on the translational efficiency.

### Loss of *Cnot8* increases transcript stability in gastruloids

We next explored the CCR4-NOT complex’s canonical role in transcript destabilization via deadenylation^57^. To capture a more mature time point of gastruloid development, we performed an RNA stability assay at 96 h post-aggregation. Gastruloids were treated with Actinomycin D to globally inhibit transcription, and transcript abundance was quantified at 0, 2 and 4 h by RNA sequencing (Fig. 6L). We first assessed transcriptome-wide differences between *Cnot8* knockout and control gastruloids at 96 h (corresponding to 0 h Actinomycin D time point). We observed widespread transcriptomic changes, including a dramatic loss of Wnt signaling components and regulators, indicative of global repression of Wnt signaling (Fig. S7E, F). Additionally, markers of anterior-posterior axis formation such as *Hox* genes and genes essential for mesodermal differentiation, like *Msgn1*, were depleted (Fig. S7E). Conversely, markers of neuronal fates such as *Pou3f1* and *Tubb3* were enriched, alongside increased expression of notochordal markers such as *Foxa2*, *Chrd* and *Tppp3* (Fig. S7E).

We then determined the impact of *Cnot8* loss on transcript stability. Consistent with its role in deadenylation, *Cnot8* knockout led to a pronounced increase in transcript half-lives, with median half-lives increasing across hundreds of genes (Fig. 6M, N). Interestingly, *Nodal* and *Zic3* were among the transcripts with increased stability (Fig. 6P–R), consistent with the observed poly(A) tail elongation in *Cnot8*-deficient gastruloids and implicating *Cnot8* in modulating key developmental regulators through mRNA decay (Table S7).

In addition, we observed transcript stabilization of *Axin1, Eomes* and *Fgf8*, all of which have roles in the formation, patterning and migration of the primitive streak and axial mesoderm (Fig. S7G–O)^63,64^. Stabilization of *Axin1*^64,65^ may underlie at least partly the global downregulation of Wnt signaling, while stabilized *Nodal* and *Fgf8* could indicate activation of TGFβ and FGF signaling. Notably, TGFβ activity may be further influenced by changes in the stability of multiple TGFβ regulators, including repressors such as *Skil*, *Eid2* and *Tgif2* all of which were stabilized in *Cnot8* knockout gastruloids (Figs. 6N and S7J–M). Given that temporally and spatially regulated TGFβ is critical for the differentiation of the notochord in embryo models and embryos^53,55,59^, the effect of *Cnot8* knockout on many TGFβ-related transcripts may underlie the ectopic notochord differentiation seen in *Cnot8* knockout gastruloids. In line with this notion, *Zic3* has also been strongly implicated in node formation, migration^66,67^ and notochord specification in *Xenopus*^60^ and mice^66^.

**Fig. 7:**
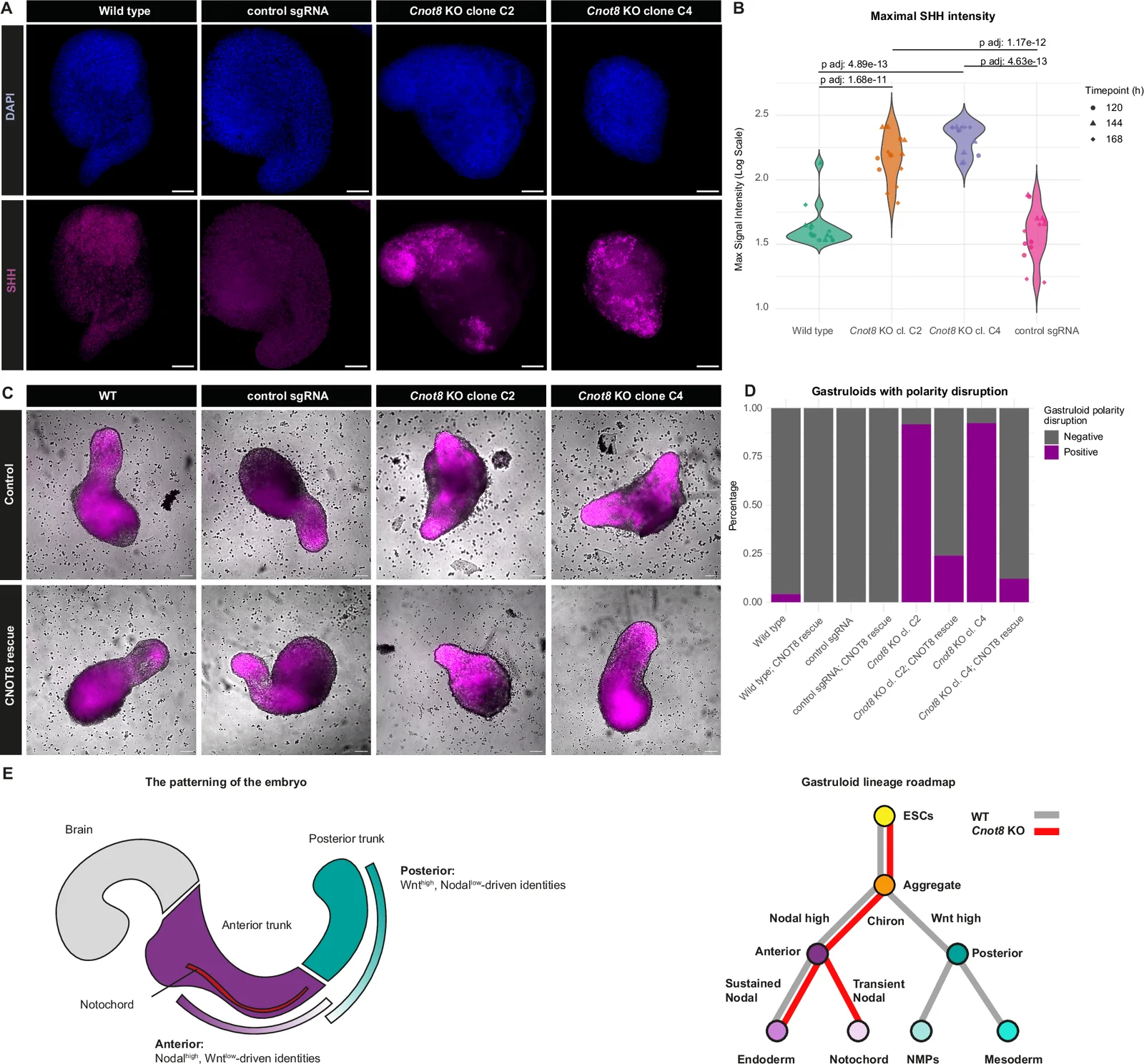
*Cnot8* loss induces ectopic notochord markers and is rescued by CNOT8 re-expression. **A** Immunostaining for *Shh* in wild-type, control sgRNA or *Cnot8* knockout of 120 h gastruloids. Scale bars, 100 μm. Images are representative; the number of gastruloids imaged across 120, 144 and 168 h was *n* = 5 per genotype and time point. Image analysis was performed blind to condition. **B** Maximal *Shh* intensity in wild-type, control sgRNA or *Cnot8* knockout gastruloids. *p* value, one-way ANOVA test. *n* = 5 per genotype and time point. **C** Representative brightfield and *T*-mCherry fluorescence images of *Cnot8* knockout gastruloids at 120 h with and without re-expression of CNOT8. Scale bars, 100 μm. *n* = 8–9 per genotype. Images are representative; image analysis was performed blind to condition. **D** Quantification of polarity defects across the 120 h, 144 h and 168 h *Cnot8* knockout gastruloids with and without re-expression of CNOT8. *n* = 8–10 per genotype and time point. **E** Proposed model and lineage roadmap for *Cnot8*-mediated body plan formation. Deletion of *Cnot8* prolongs the half-life of transcripts that specify anterior identity (*Nodal, Axin1, Zic3*), shifting developmental fates toward primitive-streak and anterior cell fates, including notochord. This stabilization may indirectly suppress Wnt pathway activity, leading to diminished expression of posterior markers (*Hox*, *Cdx*) and impaired posterior axis formation.

To further validate the presence of notochord-like cells in *Cnot8* knockout gastruloids, we directly assessed notochord marker expression. Immunostaining for the notochord marker *Shh* revealed no detectable signal in wild-type or control sgRNA gastruloids, whereas both *Cnot8* knockout clones exhibited a pronounced *Shh* signal (Fig. 7A). Quantification of maximal staining intensity confirmed a clear increase in Shh levels in the *Cnot8* knockout clones (Fig. 7B). To verify that these phenotypes are specifically attributable to *Cnot8* depletion, we re-expressed CNOT8 in both knockout clones. While *Cnot8* knockout gastruloids displayed the expected strong elongation defect, re-expression of CNOT8 largely restored normal elongation, supporting a direct role for CNOT8 in regulating this developmental phenotype (Fig. 7C, D).

Finally, to assess the developmental potential of *Cnot8* knockout ESCs in vivo, we generated chimeric embryos by injecting GFP-labelled *Cnot8* knockout ESCs into blastocysts. At E10.5, GFP signal indicated that *Cnot8* knockout cells contributed extensively to host embryos; however, *Cnot8*-*KO*/WT chimeras showed considerable variability in size and morphology, with some appearing delayed or potentially abnormal (Fig. S7N). Analysis of E10.5 sections confirmed widespread distribution of GFP-positive *Cnot8* knockout cells across embryonic tissues, with heterogeneous levels of chimerism (Fig. S7O). Although further studies will be required to define their lineage identity and determine the developmental competence of *Cnot8* knockout-derived cells in vivo, these findings suggest that their presence in chimeric embryos may be associated with developmental abnormalities.

Together, these data suggest a profound impact of *Cnot8*-mediated deadenylation on lineage specification and body axis formation in gastruloids. Beyond its role in regulating the exit from pluripotency, our scRNA-seq dataset indicates an essential role of *Cnot8* in mesodermal patterning, with *Cnot8* knockout organoids lacking paraxial mesoderm and its derivatives, while gaining node-like and axial mesodermal tissues such as the notochord. This shift profoundly alters the signaling landscape of *Cnot8* knockout gastruloids, from a Wnt-high to a Wnt-low, *Nodal*- and BMP-inhibitor-rich state (Fig. 7E). Our findings establish the transcript poly(A) tail length regulation as a key mediator of embryonic patterning and body plan formation.

## Discussion

Here, we leverage the scalability and reproducibility of mouse gastruloids to systematically investigate the role of post-transcriptional regulation in germ layer differentiation. By developing a single-cell CRISPR screening strategy, we identify regulators of germ layer commitment. In the endoderm, this includes factors such as *Musashi-2* (*Msi2*), an RNA-binding protein implicated in the regulation of cell proliferation and differentiation in the context of cancer^68,69^ and development^70^. Our data extend *Msi2’s* role to early endodermal lineage specification during mammalian embryogenesis. *Paip2b*, a poly(A)-binding protein interacting protein previously implicated in inhibiting cap-dependent translation^71^, was another candidate showing marked depletion in the NMP compartment. Importantly, depletion of the CCR4-NOT components *Cnot9* and, more prominently, *Cnot8*, but not its paralogue *Cnot7*, results in profound defects in mesoderm differentiation and embryo patterning. These results complement its previously described role in mESC pluripotency exit^49^ and align with the peri-gastrulation lethality observed in *Cnot8* knockout embryos, a phenotype not seen in *Cnot7* knockout mice, which remain viable^49^. Importantly, prior work in non-embryonic systems indicates functional redundancy between CNOT7 and CNOT8, rendering the present observations particularly striking^72,73^. Given its canonical function as the deadenylase subunit of the CCR4-NOT complex^57^, the principal deadenylating machinery in the cell, our findings unearth a hitherto unappreciated layer of post-transcriptional control over germ layer specification and body plan formation.

*Cnot8* knockout organoids show dramatic morphological defects, including a lack of axial polarity and sustained *T/Brachyury* expression beyond normal developmental time points. Importantly, we document the emergence of notochord cells starting at 96 h, a cell type absent in mouse gastruloids without modified culture conditions^53,55^. These cells express functional signals such as *Shh*, *Nog*, and *Chrd*, along with cilia-associated genes, suggesting organizer-like activity, thereby reprogramming patterning cues across the entire organoid. Our findings imply a crucial role of *Cnot8* in gastruloid patterning, mediated by its canonical role as deadenylase in poly(A) tail shortening.

Regulation of poly(A) length is crucial for oocyte activation and pre-implantation development, primarily by regulating translational efficiency of mRNAs^61,62,74,75^. During these early stages, particularly around the transition from the maternal to the zygotic genome, poly(A) tail lengths and transcript stability are decoupled. In contrast, later in development and in mature organisms, poly(A) tail length is no longer thought to influence translation, but instead strongly impacts transcript stability^74,75^, with CCR4-NOT-mediated deadenylation promoting transcript destabilization and clearance^76^. Using Nanopore direct RNA sequencing^77^, scRNA-seq, ribosome profiling^78,79^ and RNA degradation assays, we demonstrate that *Cnot8* loss has negligible effects on translation but rather leads to widespread transcript poly(A) tail elongations and transcript stabilization. This includes transcripts such as *Nodal*^58^ and *Zic3*^60,66^, which are crucial for primitive streak development and its patterning^59,60,66,67^. Thus, our findings implicate *Cnot8* in regulating the turnover of patterning transcripts beyond the exit from naive pluripotency.

Given *Zic3*’s involvement in node and notochord formation in *Xenopus*^60^ and mice^66,67^, and in left-right symmetry in humans^80–82^, it is tempting to speculate that *Zic3* represents a strong candidate mediator of the mesodermal patterning defects. In *Xenopus*, *Zic3* also antagonizes Wnt signaling^60^, potentially contributing to the diminished Wnt activity observed in *Cnot8*-deficient organoids. The elevated expression of *Zic3* in 120 h progenitor populations supports the hypothesis that *Cnot8* loss may partly phenocopy *Zic3* overexpression, driving aberrant mesodermal patterning. In addition, Nodal signaling is crucial in determining gastruloid patterning^25,59^ and a recent study has identified an antagonistic interaction between Wnt signaling and Nodal signaling in establishing the gastruloid body plan^59^. Ectopic activation of Nodal signaling has been shown to drive early primitive streak characteristics in gastruloids, enabling the differentiation of more anterior, early primitive streak-derived structures, which include the notochord. Although direct *Zic3* overexpression data in mammals are lacking, our results raise the possibility that co-stabilization of *Nodal* and *Zic3* may be central to the developmental defects seen in *Cnot8* knockouts. This combination of active Nodal and repressed Wnt may drive differentiation of anterior cell lineages typically derived from the early primitive streak (Fig. 7E).

Collectively, our findings unveil the regulation of mRNA polyadenylation as a central driver in integrating intrinsic and extrinsic cues to govern mammalian body plan formation and position gastruloid-based single-cell CRISPR screening as a powerful platform for systematic discovery of developmental regulators.

## Methods

All animal procedures were conducted in accordance with an authorized animal experimentation protocol that has been approved by the Federal Food Safety and Veterinary Office and the Cantonal Veterinary Office of Zurich under license number ZH103/25.

### Mouse embryonic stem cell culture

The dual T/Brachyury-mCherry, SOX1-GFP reporter (SBR) mouse ES cell line was cultured in tissue culture treated 6-well plates (Thermo Scientific, 140675)^27^, in DMEM with Glutamax (Gibco, 7001569) supplemented with 10% FCS (2-01F10, BioConcept), Penicillin-Streptomycin (100 U/ml, 100 µg/ml respectively), non-essential amino acids (0.1 mM, Gibco, 11140050), sodium pyruvate (1 mM, Thermo Scientific, 11360070), beta-mercaptoethanol (0.11 mM, Gibco, 21985023), 3 µM CHIR99021 (SML1046), 1 µM PD0325901 (S1036), 100 U/mL LIF (produced in house). Cells were split every 48–72 h by Accutase treatment followed by centrifugation at 200 × *g*, 4 min and seeding between 75,000 and 150,000 cells per well. SBR mESCs were kindly provided by the Deluz Lab and are based on CGR8 mouse ES cells (male).

### Gastruloid culture

Gastruloids were generated as described previously. In short, murine embryonic stem cells (mESCs) are washed twice with PBS, followed by a detachment using Accutase (AT-104) for 4 min at 37 °C, 5% CO_2_. Cells were collected in 4.5 ml of embryonic stem cell medium with 10% fetal calf serum. Cells were centrifuged 200 × *g*, 4 min, 4 °C and then resuspended in 10 ml PBS, followed by a second spin and PBS wash. Finally, cells were resuspended in 1 ml of N2B27 and counted using a hemocytometer. A total of 40 µl of N2B27 with 300 cells per well were seeded in low adhesion 96 well plates (CLS7007-24EA) and incubated for 48 h at 37 °C, 5% CO_2_. After 48 h, gastruloids were pulsed with 150 µl of N2B27 supplemented with 3 µM CHIR99021 (SML1046-5MG). Medium was refreshed using N2B27 every 24 h for the extent of the culture (between 120–144 h). For 168 h culture, gastruloids were transferred using a cut tip P1000 pipette into 24-well plates with 500 µl of fresh N2B27 medium and incubated at 37 °C, 5% CO_2_, shaking at 30 rpm on an orbital shaker.

### Cloning of the sgRNA library into the CROP-GFP backbone

A total of 669 sgRNA sequences were ordered as an IDT oligo pool and cloned in batch using Gibson assembly (HiFi 1 Step Master Mix Kit, SGIDNA Synthetic Genomics GA1100-50) as previously described^28,29^. Following cloning and plasmid purification using the PureLink HiPure Expi Plasmid Megaprep Kit (K210008XP, Thermo), libraries were sequenced to confirm homogeneous sgRNA distribution.

### Cloning of individual guides into the CROP-GFP or CROP-Puro backbone

Individual sgRNAs were ordered as oligos from Microsynth. Oligos were annealed and cloned into the CROP-GFP or CROP-Puro backbone using T4 ligase (IVGN2104, Thermo).

### Virus production

Vesicular stomatitis virus (VSV-G) pseudotyped lentivirus was produced by calcium phosphate transfection of Lenti-X 293 T cells (TaKaRa Clontech, #632180). CROP-Library-GFP construct, or CROP-GFP with the individual sgRNAs were transfected together with the plasmids pMD2.G and psPAX2 (Addgene plasmids #12259 and #12260), as previously described^28,29^. Media containing viral supernatant was collected 46 h after initial transfection and passed through a 0.45 µm filter (Sarstedt AG, 83.1826). For the full-scale library, the viral supernatant was further concentrated ~2000-fold using a 100 kDa MW cut-off Millipore Centricon 70 Plus (Merck Millipore, UFC710008), and the individual guide virus was used without further concentration.

### Murine embryonic stem cell infection—screen and clonal cell line generation

Before infection for the screen, the virus was titrated by infecting mESCs and measuring GFP signal 3 days post-infection. After identification of a suitable virus dilution to achieve an infectivity of about 10–15%, tet-Cas9 mESCs were infected by mixing cells with virus and an infection mix (1/10 dilution of polybrene [10 mg/ml Sigma; 107689-100MG in PBS] in FBS) in a 6-well plate, followed by centrifugation at 1100 × *g* for 30 min at 37 °C. Cells were then cultured for 5 days in standard mESC conditions, followed by induction of Cas9 expression using doxycycline (2 µM) for 72 h. Cells were then recovered for 24 h, followed by gastruloid aggregation and single-cell sequencing at 120 h. For clonal cell line generation, mESCs were infected with a single-guide carrying virus, aiming for an infectivity of around 10%. Cas9 expression was induced using doxycycline for 72 h, followed by single-cell sorting using a BD Aria III cell sorter with a 70 µm nozzle. Clones were genotyped using the TIDE assay^52^.

### Gastruloid single-cell sequencing

A total of 120 h gastruloids were harvested using P1000 cut-tips and subjected to single-cell sequencing. Briefly, gastruloids were collected in 15 ml tubes, washed once with PBS and incubated with accutase for 15 mins at 37 °C. Cell suspensions were resuspended using low adhesion tips and centrifuged 200 × *g*, 4 min, 4 °C. For sorting, cells were resuspended in FACS buffer (2% FCS and 0.5 ng/μl DAPI in PBS), passed through a 35 µm filter cap into 5 ml FACS tubes and sorted for GFP^+^ populations using a BD Aria III cell sorter using a 70 µm nozzle. Sorted cells were then spun down 15 min at 400 × *g*, 4 °C and then resuspended in BD sample buffer. Unsorted gastruloids were harvested and processed the same way, resuspended in sample buffer and then passed through 35 µm cell filters 3 times. For the single-cell CRISPR screen, CROP-GFP+ cells were sorted using a BD Aria III sorter and a 70 µm nozzle. For clonal cell line scRNA-seq, cells were used directly. Single-cell suspensions were prepared for sequencing using the BD Rhapsody Single-Cell Analysis System with the BD Rhapsody Cartridge Kit (633733). Bead-based reverse transcription, amplification and sequencing library production was performed following the manufacturer’s instructions (BD Biosciences, doc ID: 210967 rev. 2.0).

### Targeted sgRNA amplification (Dial-out)

To increase sgRNA annotation in the final dataset, the sgRNA sequence was amplified from BD Rhapsody beads using a nested KAPA PCR, as described previously^28,29^. In the first PCR (PCR1), the Rhapsody beads from individual cartridges were resuspended in 200 µl mastermix (100 µl KAPA HiFi HotStart ReadyMix (Roche 07958935001), 6 µl forward primer (5′-ACACGACGCTCTTCCGATCT-3′, 10 µM), 6 µl reverse primer (5′-TCTTGTGGAAAGGACGA-3′, 10 µM), 12 µl Bead RT/PCR Enhancer reagent from BD Biosciences and 72 µl nuclease-free water) and separated into 4 separate 50 µL reactions. Conditions for PCR1: initial denaturation at 95 °C/5 min, 25 cycles of denaturation 95 °C/30 s, annealing 53 °C/30 s, extension 72 °C/20 s and final extension for 10 min at 72 °C.

PCR1 products were pooled, followed by bead removal using a magnet and amplicon cleanup by Agencourt AMPure XP beads (Beckman Coulter, A63881) following the manufacturer’s instructions. Three µl of purified PCR1 product was used as template for PCR 2 and mixed with 47 µl of PCR2 mastermix (25 µL KAPA HiFi HotStart Ready Mix, 2 µl forward primer (5′- ACACGACGCTCTTCCGATCT-3′, 10 µM), 2 µl reverse primer (CAGACGTGTGCTCTTCCGATCTCTTGTGGAAAGGACGAAACA*C*C* G-3′, 10 µM), 18 µl nuclease-free water). PCR2 conditions were: Initial denaturation 95 °C/3 min, followed by 10 cycles of denaturation 95 °C/30 s, annealing 60 °C/3 min, extension 72 °C/60 s, and final extension of 5 min/72 °C. PCR2 product was purified by Agencourt AMPure XP beads and purified PCR2 product was used for indexing PCR. Indexing PCR followed the BD Biosciences ‘mRNA Targeted Library Preparation’ protocol (doc ID: 210968 rev. 3.0).

### Bulk RNA-sequencing

RNA was isolated from ribosome profiling lysate buffer lysates using Direct-zol RNA Kit (Zymo Research, R2052), according to the manufacturer’s instructions. Purified RNA was sent to the Functional Genomics Center Zürich or Novogene for library preparation and sequencing.

### Ribosome profiling

Ribosome profiling experiments were performed as previously described^29,79^ with minor modifications for gastruloids. In short, gastruloids were harvested at indicated time points using cut P1000 tips and collected into 15 ml tubes. Gastruloids were washed 2 times in PBS + CHX, supernatant was removed, and organoids were flash frozen in liquid nitrogen. Frozen organoids were then resuspended in ribosome lysis buffer (1x mammalian polysome buffer, 1 mM DTT, 1% Triton X-100, 0.5% NP40, 25 U/µL Turbo DNase, 100 µg/mL CHX), incubated for 5 min on ice, followed by 5 min spin at 20,000 × *g* at 4 °C. Supernatant was collected and flash frozen in liquid nitrogen, before being stored at −80 °C for downstream processing.

For ribosome profiling library preparation, samples were thawed and subjected to RNAse digest (1 U/µg of RNase 1 (Epicentre/Lucigen, 10 U/µL) for 45 min at room temperature, which was stopped by adding 4 µL of SUPERase Inhibitor (20 U/µL, SUPERase-In RNase Inhibitor Thermo Scientific AM2694). Ribosome-protected footprints were first purified using Amersham MicroSpin S-400 HR columns (Cytiva, 27514001) and then isolated using Direct-zol RNA Kit, according to the manufacturer’s instructions. Total RNA concentration was measured using Qubit RNA Broad Range Kit (Invitrogen, Q10210). Samples were then loaded onto a 15% TBE-UREA gel (Invitrogen, EC68852BOX) and ribosome-protected footprints were excised and extracted using the 20 nt, 22 nt, 27 nt, 30 nt and 60 nt oligonucleotides as markers (1 µL of 10 µM dilution in 19 µL RNase-free H2O). Extraction from gel pieces was performed overnight using RNA extraction buffer (300 mM NaOAc pH = 5.5, 1 mM EDTA, 0.25% v/v SDS). Extracted footprints were subjected to T4 PNK (NEB, M0201S) end-healing for 1 h at 37 °C. Linker ligation was performed by combining 20 µM of barcoded preadenylated linker with T4 RnI truncated K227Q (NEB, M0351L) for 3 h at 22 °C (50% w/v PEG-8000, 10X T4 RNA ligase, preadenylated linker, T4 RnI truncated K227Q). Samples were then pooled to reduce the sample number. This was followed by ribosomal RNA (rRNA) depletion by mixing 10 µL of sample with 10 µL rRNA depletion oligos (2 µM), incubating for 2 min at 80°C, followed by a gradual decrease in temperature to 25 °C and incubation with MyOne Streptavidin dynabeads (Life Technologies, 65001) to remove rRNA depletion oligos. SuperScript IV Reverse Transcriptase (Invitrogen, 18090050) was used for reverse transcription of rRNA-purified samples. cDNA was then run over a 15% TBE-UREA gel, and footprints were excised and extracted using DNA extraction buffer (10 mM Tris-HCl pH = 8, 300 mM NaCl, 1 mM EDTA) overnight. Extracted cDNA was circularized using CircLigase II (100 U/µL, Biosearch, CL4115K) for 1 h at 60 °C, followed by PCR amplification using Phusion High-Fidelity DNA polymerase (Thermo Scientific, F530S). After amplification, amplicons between 6 and 12 cycles were visualized using an 8% TBE non-denaturing polyacrylamide gel for the determination of the ideal number of cycles. This was followed by large-scale PCR using barcoded indexing primers and between 7 and 9 cycles. A large-scale PCR product was run over an 8% polyacrylamide gel (Invitrogen, EC61252BOX). PCR product was excised from the gel and extracted using DNA extraction buffer by incubating overnight. DNA was then recovered and precipitated using GlycoBlue™ Coprecipitant (1:50,000, 15 mg/mL, Thermo Scientific, AM9516) and resuspended in 10 mM Tris-HCl pH = 8. DNA quantification and quality control were performed using Qubit 1X dsDNA High Sensitivity Assay (Invitrogen, Q33230) and Bioanalyzer High Sensitivity DNA kit (Agilent, 5067-4626). Samples were pooled for sequencing.

### Nanopore direct RNA sequencing

Samples for Nanopore direct RNA sequencing were harvested at 72 h of gastruloid development. Gastruloids were harvested using cut P1000 tips, collected in 15 ml tubes and washed 2 times in PBS + CHX. Supernatant was removed, and organoids were flash frozen in liquid nitrogen. Frozen organoids were then resuspended in ribosome lysis buffer (1x mammalian polysome buffer, 1 mM DTT, 1% TritonX-100, 0.5%NP40, 25U/µL Turbo DNase, 100 µg/mL CHX), incubated for 5 min on ice, followed by 5 min spin at 20,000 × *g* at 4 °C. Supernatant was collected and flash-frozen in liquid nitrogen before being stored at −80 °C for downstream processing. RNA was isolated from ribosome profiling lysate buffer lysates using Direct-zol RNA Kit (Zymo Research, R2052), according to the manufacturer’s instructions. Nanopore direct RNA-sequencing library preparation was performed using the Direct RNA Sequencing Kit (SQK-RNA004) according to the manufacturer’s instructions. Samples were loaded onto MinION flow cells (FLO-MIN114).

### RNA degradation experiments

Gastruloids at 96 h of development were treated with 10 µM Actinomycin D (Thermo Scientific, 11805017) in N2B27, and gastruloids were harvested after 0, 2 and 4 h of treatment. Gastruloids were harvested at the indicated time points using cut P1000 tips and flash frozen. Frozen organoids were then resuspended in ribosome lysis buffer without cycloheximide (1x mammalian polysome buffer, 1 mM DTT, 1% Triton X-100, 0.5%NP40, 25 U/µL Turbo DNase), incubated for 5 min on ice, followed by 5 min spin at 20,000 × *g* at 4 °C. Supernatant was collected, and RNA was purified using the DirectZol RNA kit (Zymo Research, R2052). Purified RNA was submitted for library preparation and sequencing to Novogene.

### Bulk RNA sequencing of sorted cells

T/Brachyury-P2A-mCherry+ cells were sorted using a BD Aria III sorter and a 70 µm nozzle. Sorted cells were spun down, and the pellet was suspended in Trizol. RNA was purified using the DirectZol RNA kit, including on-column DNase digest and purified RNA was submitted to Novogene for library preparation and sequencing.

### Immunostaining and imaging

Gastruloids were harvested at indicated time points using cut P1000 tips, washed with PBS and then transferred into 2 ml of 4% paraformaldehyde (PFA) in PBS. Immunostaining was performed as previously described^83^. Gastruloids were fixed in PFA for 2 h at 4 °C, followed by 3 washes in PBS and 2 h blocking in PBS-FT (PBS with 10% FCS, 0.1% Triton). Primary antibodies were added in PBS-FT, and gastruloids were incubated overnight at 4 °C, with shaking. This was followed by 3 washes in PBS-FT, followed by incubation with secondary antibody in PBS-FT overnight at 4 °C, with shaking. After 3 more washes in PBS-FT, gastruloids were transferred into PBS and imaged using a Zeiss Axio Observer.

### Flow-cytometry

Gastruloids were harvested at the indicated time points using cut P1000 tips and transferred into 15 ml tubes. Media supernatant was removed, and gastruloids were washed once with PBS. Gastruloids were resuspended in 500 µl of Accutase and incubated for 10 min at 37 °C. Gastruloids were then dissociated using pipetting with low-adherence tips, followed by an additional 5 min of incubation. Cells were then centrifuged 200 × *g*, 4 min, 4 °C, supernatant was removed, and cells were resuspended in FACS buffer (2% FCS and 0.5 ng/ μl DAPI in PBS). DAPI, GFP, and mCherry signals were measured using a BD LSRFortessa™ flow cytometer.

### Western blot

TetCas9-SBR mESCs were collected from a 6-well plate. After one wash with 1x PBS (Gibco 10010-015), cells were scraped off and lyzed for 10 min with RIPA buffer (Sigma R0278) with 1X Protein Cocktail inhibitor (50X Promega, G6521) on ice. Lysate was spun for 10 min at 20,000 × *g*, and supernatant was collected. Protein lysate was mixed with NuPAGE LDS Sample buffer (4X, Invitrogen NP0007) and NuPAGE Sample Reducing Agent (10X, Invitrogen NP0009). Protein samples were denatured at 95 °C for 5 min and loaded on a 4–12% NuPage 12-well Bis-Tris Gel (Invitrogen, #7001691) using 1X NuPAGE MOPS SDS Running Buffer (20X, Invitrogen, NP0001). Following protein transfer to a nitrocellulose membrane (Cytiva Amersham Protran 0.45 µm NC, 10600002) by tank transfer at 4 °C, 30 V for 90 min, membranes were blocked using 5% skim milk at room temperature for 2 h. Primary antibody staining was performed overnight at 4 °C, followed by 3 washes with TBS-Tween 0.1%. Following the washes, membranes were incubated with secondary antibody for 2 h at 4 °C, followed by 3 additional washes with TBS-Tween 0.1%. Membranes were developed with freshly mixed ECL solutions (Amersham Cytiva, RPN2209). Antibodies used in this study: Cas9 (7A9-3A3) mouse mAb (Cell signaling #14697), Actin (8H10D10) mouse mAb (Thermo Scientific MA5-15452), Brachyury (D2Z3J) Rabbit mAb (Cell Signaling, #81694), mouse Shh (5E1, developed by Jessell, T.M. / Brenner-Morton, S. from Columbia University/HHMI, was obtained from the Developmental Studies Hybridoma Bank (DSHB) at The University of Iowa), Donkey anti-Mouse IgG (H + L) Highly Cross-Adsorbed Secondary Antibody, Alexa Fluor 647 (Catalog Number: A-31571, RRID:AB 162542) from Thermo Fisher Scientific, and Anti-mouse IgG HRP-linked antibody (Cell signaling #7076).

### Bioinformatic analysis of single-cell sequencing data

Raw sequencing data were processed as previously described^28,29^. In short, sequencing data in BCL format were demultiplexed into FASTQ files using Illumina’s bcl2fastq (v2.20). The analysis was performed with default settings, permitting up to one base mismatch in the sample barcode sequences. The resulting FASTQ files were then processed through the BD Rhapsody™ Analysis Pipeline (v1.9.1), executed on the Seven Bridges cloud genomics platform. For alignment, a custom mouse reference genome was created using Gencode vM25 (GRCm38.p6), which was augmented with the sequences of 669 single guide RNAs (sgRNAs). A custom STAR genome index was generated from this reference. Alignment was performed within the pipeline with the ‘Refined Putative Cell Calling’ option disabled. Following alignment and cell calling, the raw unique molecular identifier (UMI) counts were corrected using the Recursive Substitution Error Correction (RSEC) algorithm provided by BD Genomics to generate the final gene expression matrices for downstream analysis. For a subset of analyses, the WTA reads were aligned and quantified with STARsolo (STAR v2.7.11b) against the Gencode vM25 (GRCm38.p6) genome, using the BD Rhapsody bead cell-barcode and UMI configuration (CB_UMI_Complex mode with single-mismatch whitelist matching).

### Bioinformatic analysis of dial-out data

PCR dial-out data were processed using an in-house custom Python script as previously described^28,29^. Three 9-nucleotide cell barcodes were extracted from Read 1, allowing for up to one mismatch for each barcode (Hamming distance of 1). The script retrieved the 8-nucleotide UMI sequences from Read 1 as per the BD manual. This was followed by sgRNA annotation in Read 2 for each valid barcode, using an exact match search for the 20-nucleotide sgRNA sequence of the 669 sgRNAs in the screen or the 5 sgRNAs used to demultiplex the individual clonal cell lines in the Cnot7 and *Cnot8* knockout scRNA-seq experiment. Following UMI deduplication, sgRNA UMI counts per cell were computed. In case of multiple sgRNAs detected for an individual cell, specific sgRNA assignment was only performed if the cell’s UMI count exceeded the 99th percentile.

### Single-cell RNA-seq data analysis

Doublets were identified and removed from each sample using the Scrublet Python package^84^, with expected doublet rates adjusted based on estimates from the BD Rhapsody scanner. For downstream processing, only cells classified as singlets and containing a detected sgRNA were retained. Cells were further filtered based on quality control metrics, requiring UMI counts >500, UMI counts below the 99th percentile to remove outliers, and mitochondrial gene content <20%. The filtered dataset was processed using the standard Seurat pipeline. Gene expression was normalized using the NormalizeData function, which scales counts to 10,000 per cell, followed by a natural log transformation. The top 2000 highly variable genes were identified using FindVariableFeatures, and the data were scaled with ScaleData. Principal Component Analysis (PCA) was performed with RunPCA, and the top 50 principal components were used to construct a nearest-neighbor graph (FindNeighbors). Cell clusters were identified using the FindClusters function with Leiden modularity optimization^85^. Data was visualized in two dimensions using Uniform Manifold Approximation and Projection (UMAP).

Batch correction for the screen data was performed using Harmony. Marker genes for each cluster were identified with FindAllMarkers, and clusters were manually annotated based on the expression of canonical cell type markers. Differential expression analysis was conducted using both the MAST package and the Wilcoxon rank-sum test. Kernel density of gene expression was estimated using the Nebulosa package. The dataset was integrated with an external embryo reference using the MouseGastrulationData package^4^ and the FindTransferAnchors and MapQuery functions in Seurat. Cell signaling pathways were analyzed with CellChat, and pseudotime trajectories were inferred using Monocle3^86–88^.

### Bulk RNA sequencing data analysis

Paired-end bulk RNA-seq data were processed using the nf-core/rnaseq pipeline (v3.17.0)^89^. Briefly, raw read quality was assessed using FastQC, followed by adapter and quality trimming with TrimGalore. Reads were aligned to the Gencode vM25 (GRCm38.p6) reference genome using the STAR aligner. Transcript abundance was quantified using Salmon. An aggregated quality control report for the entire analysis was generated by MultiQC.

### Ribosome profiling data analysis

Ribosome profiling data were processed as previously described^29^. Ribosome footprints were extracted from FASTQ files using UMI-tools and a custom regular expression pattern. Footprints with a minimum length of 20 nt were retained. Reads aligning to ribosomal and transfer RNAs (rRNA and tRNA) were removed using Bowtie2 (v2.2.5). Reads remaining after rRNA/tRNA removal were aligned to the Gencode vM25 (GRCm38.p6) reference genome using STAR (v2.7.11a), allowing for one mismatch (--outFilterMismatchNmax 1) and up to 20 multi-mapped reads (--outFilterMultimapNmax 20). PCR duplicates were removed based on UMIs using the UMI-tools dedup command, and reads mapping to coding sequence (CDS) regions were counted with featureCounts.

Quality control and periodicity analysis were performed with the Ribowaltz package^90^. Deduplicated, transcriptome-aligned BAM files were used as input. P-site offsets were calculated using the psite command in auto mode with a flanking region of 6 nucleotides. Standard Ribo-seq quality control plots, including periodicity analysis, were generated using the package’s functions.

### sgRNA enrichment and depletion calculation

The number of sgRNAs in the amplicon sequencing data was counted by the count command of the MAGeCK package^40^. To calculate enrichment and depletion of sgRNAs, we used amplicon counts detected in mESCs before doxycycline induction (preDOX) and before gastruloid formation (preGast) compared to the number of cells per sgRNA at 120 h. Additionally, cell-type-specific depletion and enrichment were computed by comparing cells in one cell type vs all other cells.

### Differential expression

For single-cell differential expression analysis, the DESeq2^91^ interface with its glmGamPoi R package^92^ interface was used, which employs a Gamma-Poisson generalized linear model on the data. The *p* values were corrected for false discovery rate using the Benjamini-Hochberg procedure. Due to limited cell numbers and statistical power, uncorrected *p* values were used for downstream plots in data from the original screen. RNA-seq and Ribo-seq DE and TE analyses were performed by DESeq2 or deltaTE^93^.

### Gene ontology analysis

To identify biological processes associated with downregulated genes, Gene Ontology (GO) enrichment analysis was performed. The input gene cutoffs were adjusted to each dataset. The analysis was conducted using the enrichGO function from the clusterProfiler R package^94^. The function performed an over-representation test on the Biological Process (BP) ontology, using the mouse annotation database (org.Mm.eg.db) with official gene symbols as input. The resulting *p* values were adjusted for multiple hypothesis testing. Selected significant (*p*adj <0.05) GO terms were shown in Figures.

### Nanopore data bioinformatic analysis and poly(A) length estimation

Raw nanopore reads in pod5 format were base-called and mapped to the reference mouse genome GRCm38 (primary assembly, obtained from the GENCODE website) using the Dorado software [https://github.com/nanoporetech/dorado] version 0.8.3 with the model rna004_130bps_sup@v5.1.0. Dorado is the software package developed and supported by Oxford Nanopore Technologies for the processing of raw nanopore data obtained on their platform. Dorado uses a deep learning-based algorithm for base-calling (transforming nanopore current data to nucleotide sequence), minimap2 aligner^95^ for read alignment to the genome, and a separate heuristic algorithm for estimating poly(A) tail length in each read. Importantly, successfully estimated tail lengths are stored as the “pt:i” tags in the output.bam files, while the base-called sequences of reads contain only short fragments of poly(A) tails, which are not informative for the actual length of the poly(A) tails. Technically, the following command was used: dorado basecaller rna004_130bps_sup@v5.1.0 $pod5 --estimate-poly-a --poly-a-config polya_config.toml --mm2-opts “-x splice -Y” --reference GRCm38.primary_assembly.genome.fa

Identification of the poly(A) tails in the reads was configured to tolerate non-adenine gaps up to 3 nt in length: tail_interrupt_length = 3. We further retained only uniquely mapped reads with mapping quality above or equal to one (in minimap2 output, quality=0 indicates that a read is a multimapper that aligns equally well to more than one genomic locus) and present “pt:i” tag. In total, ~8.1 M reads were retrieved from four samples (two bioreplicates for each of the two conditions: Cnot8 KO and Control), with ~1.7 to ~2.5 M reads per sample.

To identify the genes from which the reads originated, the following procedure was applied: without considering splicing configuration (“CIGAR” string in the alignment), genomic coordinates of read alignments were intersected with the genomic coordinates of genes from the comprehensive GENCODE annotation of the mouse genome version vM25^96^ using bedtools intersect version v2.31.1^97^. After requiring the minimum overlap to be 0.75 of the read length, ~5.5 M reads ( ~ 68%) were assigned to genes. Only ~230 K reads ( ~ 2.9%) were assigned to more than one gene. The resulting table of per-read poly(A) tail lengths was further analyzed in R. First, reads were filtered to retain those with a quality score > 10 and a mapping quality > 10. For robust statistical comparison, only genes with at least 50 assigned reads in every sample were included in the differential analysis.

To identify genes with significant changes in poly(A) tail length, a Wilcoxon rank-sum test was performed for each gene, comparing the distributions of individual read poly(A) tail lengths between the Cnot8 KO and control conditions. The effect size was calculated as the log_2_ fold change of the median poly(A) tail length of the KO versus the control. P-values were adjusted for multiple testing using the Benjamini-Hochberg method to calculate the False Discovery Rate (FDR).

### RNA half-life calculation and statistical analysis

RNA degradation rates were determined from gene expression data quantified in Counts Per Million (CPM). First, genes with low expression, defined as having a mean of less than 10 CPM across all 0-h timepoint replicates, were excluded from the analysis. The data normalization was performed in a two-step process. First, to account for initial expression differences, CPM values for each gene at 2 and 4 h were normalized to the CPM value at the 0-h timepoint within the same biological replicate. Second, to correct for systemic variations across samples, a scaling factor was calculated for each sample by taking the median of the T0-normalized values from a predefined set of 30 stable housekeeping genes. All T0-normalized expression values for every gene were then divided by their corresponding sample’s scaling factor.

### For each gene within each individual replicate, a first-order exponential decay model

$N\left(t\right)={N}_{0}{e}^{-{kt}}$ was fitted to the fully normalized expression values at 0, 2, and 4 h using the curve_fit function in the SciPy library. To ensure the reliability of the decay kinetics, fits with a coefficient of determination (R^2^) below 0.75 were discarded. The RNA half-life (${t}_{1/2}$) was then calculated from the decay rate constant (k) for each valid fit using the formula ${t}_{1/2}=\frac{{{\mathrm{ln}}}(2)}{k}$.

To identify significant differences in RNA stability, the calculated half-lives were first log2-transformed. A two-sample Welch’s t-test was performed between the knockout and control groups for each gene with at least two valid half-life values per condition. The resulting p-values were adjusted for multiple comparisons using the Benjamini-Hochberg procedure to control the False Discovery Rate (FDR). Genes with an FDR less than 0.1 were considered to have a statistically significant difference in RNA half-life.

### Blastocyst injections, embryo transfer and assessment of chimerism

All animals were housed in Allentown cages under standard room temperature conditions with 50-60% relative humidity, a 12-hour light-dark cycle, and unrestricted access to food and water. B6 Albino mice (*B6(C)/Rj-Tyrc/c*) from Janvier Labs (France) were used for blastocyst production. Additionally, Rj:SWISS mice (Janvier Labs, France) were used as foster females, which were mated with Rj:SWISS vasectomized males (Janvier Labs, France) to induce pseudopregnancy. For blastocyst production, superovulation was induced in female mice by intraperitoneal injection of 5 IU pregnant mare serum gonadotropin (PMSG; ProSpec, HOR-272), followed 48 h later by 5 IU human chorionic gonadotropin (hCG; ProSpec, HOR-250). Females were then paired with single-housed males. Embryos were recovered at the blastocyst stage by uterine flushing in M2 medium (Sigma-Aldrich, M7167). Recovered blastocysts were maintained at 37 °C in 5% CO₂ in either EmbryoMax Advanced KSOM Embryo Medium (Sigma-Aldrich, MR-101-D) or EmbryoMax KSOM Mouse Embryo Medium (Sigma-Aldrich, MR-106-D) until injection, as previously described^98^. For blastocyst injections, *CNOT8*-KO ESCs were cultured on DR4 “feeder cells” in “Serum + 2i”, as previously described^99^. On the day of injection, ESCs were dissociated with 0.05% trypsin–EDTA (Thermo Fisher Scientific, 25300054) for 3–5 min at 37 °C in 5% CO₂, then transferred to 0.1% gelatin-coated dishes for 1 h to deplete feeders. The cell suspension was then collected, pelleted by centrifugation at 130 × *g* for 5 min, and resuspended in ESC medium. Cells were kept on ice until injection. Around 10-15 ESCs were injected per blastocyst. A total of 10–17 injected blastocysts were transferred into recipient females using non-surgical embryo transfer (ParaTechs, 60010), as previously described^99^. Chimeric embryos were identified by fluorescence from the appropriate reporter(s).

### Embryo isolation and visualization

Embryos were collected immediately following euthanasia. Isolated embryos were briefly washed in ice-cold PBS and then imaged using a Nikon SMZ-1270 stereomicroscope equipped with a QImaging Retiga R1 camera. GFP fluorescence was displayed as a pseudocolor overlay using NIS-Elements software (Nikon).

### Immunofluorescence staining of E10.5 embryo cross-sections

Freshly collected embryos were fixed in 4% paraformaldehyde (PFA) overnight at 4 °C. Samples were then embedded in OCT (CellPath, KMA-0100-00A), snap-frozen on dry ice, and stored at −80 °C until sectioning using a cryostat (Leica, CM1950) to generate 10 µm-thick sections. For immunostaining, embryo sections were washed three times in PBS and incubated for 1 h at room temperature in blocking/permeabilization buffer (2% BSA, 0.1% Triton X-100 in PBS). Antibodies were diluted in blocking buffer (2% BSA in PBS) and included anti-GFP (1:500; Invitrogen, A11122) and conjugated Alexa Fluor Plus 647 phalloidin (1:500, Invitrogen, A30107) and were applied overnight at 4 °C. The following day, sections were washed twice with PBS and incubated for 1 h at room temperature with goat anti-rabbit IgG Alexa Fluor 488 (1:500; Thermo Fisher Scientific, A11008) and DAPI (1:1000; Sigma-Aldrich, MBD0015-1ML). After two additional PBS washes, sections were mounted in ProLong Glass Antifade Mountant (Thermo Fisher Scientific, P36980) and sealed with a coverslip before visualization.

### Statistics and reproducibility

No statistical method was used to predetermine sample size. Sample sizes were chosen on the basis of standard practice in the field and the numbers required to obtain sufficient cells, reads or organoids for robust analysis, and are stated for each experiment in the corresponding figure legends. The single-cell CRISPR screen was performed in two independent biological replicates, yielding 135,392 cells after quality filtering and sgRNA assignment. Clonal Cnot7 and Cnot8 knockout experiments used two independent clonal lines per genotype, each analyzed in independent experiments as indicated in the figure legends. Bulk RNA-sequencing of FACS-sorted cells was performed on two clones per genotype; Nanopore direct-RNA and ribosome-profiling libraries were generated from two biological replicates per condition; and RNA-decay experiments comprised three biological replicates for Cnot7 knockout and two for Cnot8 knockout, each compared with control. Flow-cytometry quantification of the mCherry-high population used *n* = 4 per genotype (two clonal lines measured in two independent experiments), and organoid polarity was scored across four replicates with 48 organoids per replicate and condition. The number of gastruloids imaged for each condition in Fig. 7 is provided in the corresponding legend.

Data exclusions were limited to pre-specified quality-control criteria. In the single-cell data, doublets, low-quality and dead cells and cells with more than 20% mitochondrial gene content were removed, and only cells with an assigned sgRNA were retained. Nanopore reads were filtered to a base-calling quality score greater than 10 and a mapping quality greater than 10. No other data were excluded from the analyses.

The experiments were not randomized. Image analysis of gastruloid immunostainings (Fig. 7) was performed blind to condition; for all other experiments, the investigators were not blinded to allocation during data acquisition and analysis, as experimental groups were defined by genotype, and this information was required for sample processing.

Statistical analyses were performed in R. Enrichment and depletion in the CRISPR screen were computed with MAGeCK robust rank aggregation using permutation-based FDR. Differential gene expression was assessed with DESeq2 (likelihood-ratio or Wald tests, as indicated in the figure legends), using glmGamPoi dispersion estimation for pseudobulk analyses, with *p* values corrected for multiple comparisons by the Benjamini-Hochberg method. Single-cell marker expression was tested using the Wilcoxon rank-sum test within the MAST framework. Gene Ontology enrichment was performed with clusterProfiler enrichGO (biological process; hypergeometric test; Benjamini-Hochberg adjustment). Poly(A) tail-length differences were evaluated with two-sided Wilcoxon rank-sum tests, and maximal Shh immunostaining intensity with one-way ANOVA. The specific test, sample size, definition of error bars and measure of center for each panel are stated in the corresponding figure legends; unless otherwise indicated, statistical tests were two-sided.

### Reporting summary

Further information on research design is available in the Nature Portfolio Reporting Summary linked to this article.

## Supplementary information

Supplementary Information

Description of Additional Supplementary Files

Supplementary Data 1

Supplementary Data 2

Supplementary Data 3

Supplementary Data 4

Supplementary Data 5

Supplementary Data 5

Supplementary Data 6

Reporting Summary

Transparent Peer Review file

## Source data

Source Data

## Data Availability

The single-cell CRISPR, ribosome profiling and RNA sequencing data are available in GEO under accession number GSE302657. Source data are provided with this paper.

## Code availability

Custom analysis code used in this study is available at https://github.com/Sendoel-Lab/crop-seq-analysis.
