# Supplementary material for: Regulation of mRNA poly(A) tail length governs mammalian body plan formation in gastruloids
Source: Nat Commun. 2026 Aug 29;17:10390. doi: 10.1038/s41467-026-76897-3 (PMC13627704; doi:10.1038/s41467-026-76897-3)
Supplement: Supplementary file 2 [file 41467_2026_76897_MOESM11_ESM.pdf]

nature portfolio

Peer Review File

Regulation of mRNA poly(A) tail length governs mammalian body plan formation in gastruloids

Corresponding Author: Professor Ataman Sendoel

This file contains all reviewer reports in order by version, followed by all author rebuttals in order by version.

Version 0:

Reviewer comments:

Reviewer #1

(Remarks to the Author)
In this paper, Taborsky et al. use an exquisitely elegant and advanced approach to identify regulators of embryonic development using gastruloids as a model system. They identify Cnot8 as a key regulator of mesodermal differentiation. This conclusion is fully supported by the data. The amount and quality of data included is impressive.

In terms of the significance of the findings, I considered the manuscript asking whether (and how) it advances knowledge of how the Ccr4-Not complex regulates deadenylation and gene expression programmes.

Cnot8 encodes a catalytic subunit of the Ccr4-Not complex. This complex has been identified as the major cytoplasmic deadenylase in eukaryotic cells. The complex contains two catalytic nuclease subunits: a DEDD-type (encoded by the paralogues CNOT7 or CNOT8) and an EEP-type (CNOT6 or CNOT6L) deadenylase. It is currently unclear why each deadenylase is encoded by two paralogous genes. Several studies indicated that the paralogues have largely overlapping functions (e.g. Mostafa et al. RNA Biol, 2020, Aslam et al., Mol Biol Cell, 2009). However, it is clear that the mere existence of two paralogues indicates that the two genes must have unique functions. Indeed, Cnot7 knockout mice are viable, while Cnot8 knockouts die before E10.5.

Recently, Quan et al. (Nucleic Acids Res, 2020) investigated the unique role of Cnot8 in embryonic development and identified that deletion of Cnot8 cannot be rescued by expression of Cnot7, that the catalytic activity of Cnot8 is required, and incorporation of Cnot8 into the Ccr4-Not complex as well. Moreover, they showed that Cnot8 is a regulator of global poly(A) tail length in some scenarios.

Taborksy et al extend these observations by also looking the role of Cnot9 (another Ccr4-Not subunit) and at possible effects on translation using ribosome profiling. The finding that knockout of Cnot8 (or Cnot7) does not result in significant changes in translation is interesting, as the Ccr4-Not complex (incl the deadenylase subunits) are also implicated in the regulation of translation.

How Cnot8 specifically functions despite its high similarity to Cnot7 (>80% identical) is unclear. Unfortunately, this study by Taborsky et al. does not answer that question, or significantly advances our insights into how Ccr4-Not functions and why there is a unique role for Cnot8 in embryonic development (or phrased differently, why Cnot7 cannot compensate in this specific scenario).

Specific comments
The title is not quite correct. The Cnot8 deadenylase is identified as the key regulator. Therefore, the title should read “Regulation of mRNA deadenylation governs mammalian body plan formation in gastruloids”, or “Regulation of mRNA poly(A) tail length governs mammalian body plan formation in gastruloids”.

It is useful to include the list of 406 genes associated with the gene ontology term “regulation of translation” (GO:0006417), the curated a list of 201 candidate genes based on their expression in gastruloids and the included 4 depletion controls and 8 known regulators of germ layer differentiation. They are not available in Figure S1. It is clear from the experiments that Cnot9 and Cnot10 were included, but were there, for example, other Ccr4-Not components in the library?

Missing: a discussion/reference to the function of a specific role of Cnot8 in contrast to observations that CNOT7 and CNOT8 are largely redundant in function in MEFs and human cancer cell lines (Mostafa et al. RNA Biol, 2020, Aslam et al., Mol Biol Cell, 2009).

Fig.S3N is not informative. Instead, it would be helpful to show the structure of the proteins (which have very similar/identical folds as shown) with highlighted the positions that are not identical/conserved/not-conserved.

Ref.51 is dated; there are several more recent reviews to choose from that are up-to-date and include the concept that the Cnot9 subunit is used to recruit deadenylase activity via interactions with RNA-binding proteins.

Fig.3B An over-representation of Cnot10 is observed in endoderm as well as an overrepresentation of Cnot9 in neural progenitors. Given that these are Ccr4-Not subunits, it would be helpful to comment briefly.

Reviewer #2

(Remarks to the Author)
Here, Taborsky et al. report a Perturb-Seq screen of ~200 genes in 3-dimensional mouse gastruloids, and they unexpectedly discover that deletion of RNA deadenylase Cnot8 leads to the emergence of notochord cells in gastruloids. This discovery is important in multiple ways. First, notochord cells are often lacking from conventional gastruloid models, and it is therefore very interesting that Cnot8 loss can enable their generation. Second, RNA deadenylation is often overlooked as a molecular mechanism that controls gastrulation and germ layer development, and the authors' work is thus quite exciting. Third, notochord cells in Cnot8-/- gastruloids express an impressive suite of archetypic markers, including Foxj1, Noto, Noggin, Chordin, Foxa2, and Brachyury, lending confidence that they are indeed notochordal in nature (Fig. 6).

While the authors do not fully resolve the molecular mechanism leading to this notochordal expansion, they show that Cnot8 deletion leads to an increase in both the poly(A) tail length and stability of the Nodal and Zic3 mRNAs. Stabilization of these two mRNAs, which encode notochord identity regulators, may contribute to the observed phenotype.

In any case, this is a strong and compelling paper that could be published in short order without fully resolving the molecular mechanism, at least in the opinion of this reviewer.

Novelty: This reviewer felt that the present study was original and novel. A previous study found that Cnot8-/- mouse embryos failed to complete gastrulation (with major defects seen by ~E7.5; https://www.ncbi.nlm.nih.gov/pubmed/35390160), but did not explore any potential axial mesoderm or notochord phenotypes. Reciprocally, recent studies have claimed to differentiate pluripotent stem cells towards notochord (https://pubmed.ncbi.nlm.nih.gov/39611739/, https://pubmed.ncbi.nlm.nih.gov/39695233/), but none of these studies invoked RNA deadenylation as a potential mechanism.

Major comments:
1. Further confirmation of notochord expansion in Cnot8-/- gastruloids:
a. In Fig. 6, it would be ideal if the authors could show 3 separate feature plots of notochord marker expression (e.g., of Noto) in control vs. Cnot7-/- vs. Cnot8-/- gastruloids. This would make it unequivocally clear that notochord markers are elevated in Cnot8-/- gastruloids, complementing the data in Fig. 6B and Fig. 6F.
b. To support the compelling scRNA-seq data of expanded notochord cells in Fig. 6, it would be very nice if the authors could consider in situ staining or immunostaining for notochord markers, to back up their transcriptomic data. At present, they show increased Brachyury-P2A-mCherry signal in Fig. 4, but as the authors are aware, Brachyury is broadly expressed in multiple cell-types and therefore is not the most specific marker.
2. Cell-type assignment of scRNA-seq clusters. Overall, this was a strong study, but this reviewer had questions about the cell-type assignment of several scRNA-seq clusters. These questions could be addressed by re-labeling clusters, or potentially re-clustering at higher resolution.
a. "Neuronal progenitor" cluster (Fig. 1B,C,H): Expresses pluripotency transcription Nanog and Pou5f1, as well as the neural crest/surface transcription factor Tfap2c (https://pubmed.ncbi.nlm.nih.gov/17258188/). It may comprise a heterogeneous mixture of pluripotent and ectoderm cells. While this cluster expresses Tubb3, this gene is also expressed in other cell-types. Plotting the expression of neuronal markers Mapt and Snap25 may help to clarify the identity of this cluster.
b. "NMP" cluster (Fig. 1B,C,H): Expresses Foxa2, which typically is not thought of as an NMP marker. Could this reflect Foxa2+ notochord or anterior primitive streak cells (https://pubmed.ncbi.nlm.nih.gov/8306889/) within this cluster?
c. "Cardiac mesoderm" cluster (Fig. 1B,C,H): Expresses Prrx1, which is a marker of limb bud progenitors, not heart progenitors (https://pubmed.ncbi.nlm.nih.gov/8101172/). Perhaps this cluster comprises a mixture of lateral mesoderm derivatives? To claim that this cluster corresponds to cardiac mesoderm, the authors should plot expression of the archetypic cardiac mesoderm transcription factor Nkx2.5.
d. "Differentiation front mesoderm" cluster (Fig. 1B,C,H): Are the authors referring to the anterior paraxial mesoderm where cells are undergoing segmentation? If so, assessing the expression of Mesp2 and other genes would be critical (https://pubmed.ncbi.nlm.nih.gov/9242490/). At present, the authors show that this cluster lacks Tbx6 and expresses Pax3, but more evidence is needed to show that these cells correspond to cells undergoing segmentation into somites, or the others should perhaps rename this cluster.
e. Fig. 6C: What is the rationale for the authors to distinguish "node" vs. "notochord" clusters?
f. Fig. 6C: What is the rationale for the authors to annotate the "CLE" cluster as such?
g. Fig. 6C: A cluster is labeled as "neural crest", but showing the expression of distinctive markers Sox9, Sox10, or Wnt1 would be helpful.

3. Potentially swapping the order of Figs. 5 and 6. In Fig. 4, the authors present preliminary evidence for increased notochord identity in Cnot8-/- gastruloids (Fig. 4G). However, Fig. 4G is not easy to interpret: it shows differential gene expression in Cnot8-/- vs. Cnot7-/- gastruloids, but ideally one would focus on Cnot8-/- vs. control gastruloids. After Fig. 4, it might be better for the authors to directly proceed to Fig. 6 (which presents very strong proof for expanded notochord cells), and then to close the manuscript with Fig. 5 (molecular mechanism).

Minor comments:
1. “Despite their promise, the application of these techniques to the embryonic system” (Introduction) — would it be clearer to replace “embryonic system” with “embryo” here?
2. “sgRNAs targeting the germ layer controls Eomes and Sox17 showed endodermal depletion (Figure 2E and 3B), while T/Brachyury and Snai1 sgRNAs were depleted in mesodermal lineages (Figure 2E), validating the high specificity of our screening strategy.” (Results) – Fig. 2E is a beautiful figure, but each row is too thin for the reader to interpret. The authors could consider showing in the supplemental figures a larger figure focusing on only a few positive control sgRNAs.
3. In the main text, the authors should consider citing the figure panels in order. At the end of pg. 6, the authors cite Fig. 2J, but at the beginning of pg. 7, Fig. 3D is mentioned next.
4. Fig. 3B — In the Perturb-Seq screen, it seems that Cnot8 is required for endoderm induction (log2 fold change = 0.75). However, in Fig. 6E, when the authors perform detailed analysis of the Cnot8-/- gastruloids, there is now a modest increase in endoderm induction. Can the authors reconcile these two? This is a minor comment, because of course one would believe the results from detailed follow-up more than the screening results. In any case, would it be helpful for the Perturb-Seq screen to show the distribution of non-targeting sgRNAs? Maybe the variability in scores seen in non-targeting sgRNAs would encompass the endoderm score of Cnot8, implying that it was not statistically significant in the endoderm readout of the screen?
5. Fig. 3G — This figure displays scRNAseq expression of genes in their gastruloids. Is Cnot8 mRNA also decreased in axial mesoderm and notochord cells in vivo? scRNAseq datasets of the gastrulating mouse embryo are available: https://pubmed.ncbi.nlm.nih.gov/30787436/ and https://crukci.shinyapps.io/mousegastrulation2018/.
6. Fig. 3E — If the reviewer understands this correctly, sgRNA deletion of T increased the number of NMPs? Is this surprising, given the central role of Brachyury/T in NMP development (https://pubmed.ncbi.nlm.nih.gov/26329597/)? Apologies if the reviewer did not understand this figure panel.
7. Fig. 4B — The authors should consider re-labeling the x-axis from “mCherry” to “T-P2A-mCherry”.
8. Fig. 4F — It would be terrific if the authors could label this cartoon to specify that T+ cells were isolated, so readers know what purple signifies.
9. Fig. 4G could be modified or replaced to make it easier to interpret.
a. To support the claim that Cnot8 deletion leads to an increase in notochord markers, the authors would have to show that notochord markers (e.g., Noto) are upregulated in Cnot8-/- gastruloids vs. control gastruloids. Instead, in Fig. 4G, the authors compare Cnot8-/- vs. Cnot7-/- gastruloids. A volcano plot of Cnot8-/- vs. control gene expression is shown in Fig. S6E, but notochord markers were not highlighted. It would be ideal if the authors could show two separate volcano plots comparing Cnot8-/- vs. wild-type, and separately, Cnot7-/- vs. wild-type.
b. In volcano plots, ideally the authors could insert labels to indicate whether the “left” or “right” of the plot signifies that a gene is upregulated (or is downregulated) in a specific genotype.
10. Fig. 6D — It would be helpful if the gene labels are spaced out more, so the reader can understand which feature plot corresponds to which gene. For instance, the left-most plot is intersected by both “T” and “Cdx2” text boxes.
11. “Additionally, notochord differentiation in a human model of trunk development has only recently been achieved under highly specialized culture conditions (49).” — It may be worth also citing a second paper on the similar subject here (https://pubmed.ncbi.nlm.nih.gov/39611739/).
12. “BMP inhibitors Chrd, Nog, and Shh” — The authors should consider editing the phrasing to clarify that Shh is not a BMP inhibitor.
13. “This transcriptional profile suggests that these notochord cells represent a functional notochord akin to the embryonic equivalent” — Given that notochord functionality was not tested in this report, the adjective “functional” should be removed here.
14. Fig. 6C — The “paraxial” and “somitic” labels are scrunched up and are a bit hard to read.
15. Fig. 6P — This is a very nice summary cartoon, but the authors may want to revisit it. The cartoon indicates that anterior regions of the embryo are specified by high Nodal and low Wnt, but as the authors are aware, it is more complicated, since high Nodal specifies the anterior primitive streak, but subsequently it seems that Nodal must likely be inhibited to specify notochord identity (https://pubmed.ncbi.nlm.nih.gov/39611739/, https://pubmed.ncbi.nlm.nih.gov/39695233/). It is definitive endoderm that requires high and sustained Nodal signaling for its specification. Since it is difficult to depict these temporally- and spatially-dynamic signaling domains on a static photo of the embryo, maybe the authors could consider alternative cartoons, like a lineage roadmap, where it is easier to label which lineages are induced by what signals.
16. If Nodal and Zic3 transcript stability is improved by Cnot8 deletion, are these mRNAs also elevated in their RNA-seq datasets?

Reviewer #3

(Remarks to the Author)
In this manuscript, Taborsky et al conduct a perturb-seq screen focused on post transcriptional regulators in mouse gastruloids to identify candidates that influence germ layer fate. They focus on Cnot8, a deadynlase whose KO surprisingly leads to the generation of notochord cells. They characterize the Cnot8 KO mutant and find that Cnot8 is involved in transcript stability of several genes that are important for embryonic patterning.

This manuscript incorporates a lot of different genomic techniques (high throughput screening, long read sequencing, ribosome profiling, etc) with a hypothesis driven design resulting in a satisfying understanding of how post-transcriptional regulation through Cnot8 deadenylation affects cell fate. This work includes useful data resources for the community including the CROP-seq screens and the translation atlas for mouse gastruloids among others. The experiments and analysis are conducted well with appropriate controls. The manuscript is well written with clear logic and is easy to follow.

Comments:

1. Reproducibility of screen results
The authors perform CROP-seq screens targeting 243 genes with two replicates. It is not entirely clear how the screen is carried out, for example: is each replicate composed of a single gastruloid or does the screen take place across several gastruloids? What is the coverage, i.e. how many cells are infected by each gRNA? Most importantly, how reproducible are the screen results? The authors need to show a scatterplot of the log fold change reported by each screen. Points can be either gRNAs or genes (where the value is the avg across gRNAs) with a correlation coefficient to measure reproducibility. This is a valuable measurement for readers to understand how well these types of screens perform where the initial population undergoes stratification towards different outcomes.

2. Cnot8 KO notochord cells in Figure 4
The authors observe an unusual phenotype in the Cnot8 KO gastruloids – the T-P2A-mCh cells do not localize to the posterior pole. They isolate these cells using FACS and perform bulk RNA-seq and conduct DEG analysis and GO enrichment to claim these cells have notochord phenotype instead of the expected NMP phenotype. Will the authors please compare the transcriptome profile from the Cnot8 KO gastruloids T-positive population directly with the notochord signature from mouse embryos and quantify their similarity? At this point in the manuscript, the authors have established the atlas data from Figure 1. I believe a comparison at this stage would be complementary to the analysis in Figure 6 because there is some location information provided by imaging. Also, Cnot8 KO cells appear in the crop-seq screen. Will the authors please comment on whether the notochord phenotype was apparent in the screen?

3. Other cell types enriched in Cnot8 KO
Inspection of Fig 6E and Supp Fig 7A reveals that “Node” also appears in addition to notochord upon KO of Cnot8. Will the authors please add a comment into the manuscript that addresses its presence because at present, it is a glaring omission. Also, it appears the neural progenitors produced in the Cnot8 KO are different from those in WT or the Cnot7 KO. Will the authors perform a DEG analysis for the neural progenitors partitioned by Cnot8KO vs rest to examine if meaningful differences exist between the two populations?

Minor points:
- Fig 2E is not informative and should be replotted with each gene/sgRNA against the sgRNA control instead of raw cell type proportions. If the visualization is kept, then consider ordering the cell types so that paraxial meso is either on top or bottom of the column.
- Fig 3D: “UpSet” not upset.
- Fig 3G: Statistical test to support claim that Cnot8 is “markedly reduced”.
- In the scRNA-seq gastruloid data (Figure 5), why are there so many more control cells that passed filter for analysis than KO cells? From the text, it appears that the same number of gastruloids went into the pipeline. Were the KO gastruloids smaller or was there a technical issue for the KO scRNA-seq libraries?
- Supp Fig 7A does not have a key to map colors to cell types

Version 1:

Reviewer comments:

Reviewer #1

(Remarks to the Author)
In the revised manuscript, the authors fully addressed my comments.
From the perspective of Ccr4-Not, the manuscript provides a second clear example where the paralogues CNOT7 and CNOT8 are not redundant. Instead, knockout of CNOT8 results in a specific phenotype not observed upon knockout of CNOT7. This is a very interesting observation, even though it is not fully established why the phenotype is only observed upon disruption of CNOT8.

Reviewer #2

(Remarks to the Author)
The authors have satisfactorily addressed our comments, and should be congratulated for a well done and interesting study.

Reviewer #3

(Remarks to the Author)
I commend the authors for their work updating the manuscript. They have addressed my concerns in a satisfactory manner.

Open Access This Peer Review File is licensed under a Creative Commons Attribution 4.0 International License, which permits use, sharing, adaptation, distribution and reproduction in any medium or format, as long as you give appropriate credit to the original author(s) and the source, provide a link to the Creative Commons license, and indicate if changes were made.
In cases where reviewers are anonymous, credit should be given to 'Anonymous Referee' and the source.
The images or other third party material in this Peer Review File are included in the article's Creative Commons license, unless indicated otherwise in a credit line to the material. If material is not included in the article's Creative Commons license and your intended use is not permitted by statutory regulation or exceeds the permitted use, you will need to obtain permission directly from the copyright holder.
To view a copy of this license, visit https://creativecommons.org/licenses/by/4.0/

We thank all three reviewers for their very constructive comments and input, which substantially improved the manuscript.

REVIEWER COMMENTS

Reviewer #1 (Remarks to the Author):

In this paper, Taborsky et al. use an exquisitely elegant and advanced approach to identify regulators of embryonic development using gastruloids as a model system. They identify Cnot8 as a key regulator of mesodermal differentiation. This conclusion is fully supported by the data. The amount and quality of data included is impressive.

In terms of the significance of the findings, I considered the manuscript asking whether (and how) it advances knowledge of how the Ccr4-Not complex regulates deadenylation and gene expression programmes.

Cnot8 encodes a catalytic subunit of the Ccr4-Not complex. This complex has been identified as the major cytoplasmic deadenylase in eukaryotic cells. The complex contains two catalytic nuclease subunits: a DEDD-type (encoded by the paralogues CNOT7 or CNOT8) and an EEP-type (CNOT6 or CNOT6L) deadenylase. It is currently unclear why each deadenylase is encoded by two paralogous genes. Several studies indicated that the paralogues have largely overlapping functions (e.g. Mostafa et al. RNA Biol, 2020, Aslam et al., Mol Biol Cell, 2009). However, it is clear that the mere existence of two paralogues indicates that the two genes must have unique functions. Indeed, Cnot7 knockout mice are viable, while Cnot8 knockouts die before E10.5.

Recently, Quan et al. (Nucleic Acids Res, 2020) investigated the unique role of Cnot8 in embryonic development and identified that deletion of Cnot8 cannot be rescued by expression of Cnot7, that the catalytic activity of Cnot8 is required, and incorporation of Cnot8 into the Ccr4-Not complex as well. Moreover, they showed that Cnot8 is a regulator of global poly(A) tail length in some scenarios.

Taborksy et al extend these observations by also looking the role of Cnot9 (another Ccr4-Not subunit) and at possible effects on translation using ribosome profiling. The finding that knockout of Cnot8 (or Cnot7) does not result in significant changes in translation is interesting, as the Ccr4-Not complex (incl the deadenylase subunits) are also implicated in the regulation of translation.

How Cnot8 specifically functions despite its high similarity to Cnot7 (>80% identical) is unclear. Unfortunately, this study by Taborsky et al. does not answer that question, or

significantly advances our insights into how Ccr4-Not functions and why there is a unique role for Cnot8 in embryonic development (or phrased differently, why Cnot7 cannot compensate in this specific scenario).

We thank the Reviewer for the encouraging words and excellent suggestions.

Specific comments
The title is not quite correct. The Cnot8 deadenylase is identified as the key regulator. Therefore, the title should read “Regulation of mRNA deadenylation governs mammalian body plan formation in gastruloids”, or “Regulation of mRNA poly(A) tail length governs mammalian body plan formation in gastruloids”.

We agree with the reviewer and now have changed the title to “Regulation of mRNA poly(A) tail length governs mammalian body plan formation in gastruloids”, as suggested.

It is useful to include the list of 406 genes associated with the gene ontology term “regulation of translation” (GO:0006417), the curated a list of 201 candidate genes based on their expression in gastruloids and the included 4 depletion controls and 8 known regulators of germ layer differentiation. They are not available in Figure S1. It is clear from the experiments that Cnot9 and Cnot10 were included, but were there, for example, other Ccr4-Not components in the library?

Thank you for this suggestion. We agree with the reviewer that these lists will be informative and have therefore added the corresponding information to the Supplementary Table S1 , including a complete list of all CCR4–NOT subunits included in the screen. Specifically, we included the 404 genes with the gene ontology term “regulation of translation” (GO:0006417), the curated list of 201 candidate genes (including the germ layer and depletion controls).

For ease of access, CCR4-NOT subunits included in the screen where: *Cnot1, Cnot2, Cnot7, Cnot8, Cnot9 and Cnot10.* Other subunits did not make the initial expression cut-off in our bulk RNA-seq data from 48h and 72h of gastruloid development.

Missing: a discussion/reference to the function of a specific role of Cnot8 in contrast to observations that CNOT7 and CNOT8 are largely redundant in function in MEFs and human cancer cell lines (Mostafa et al. RNA Biol, 2020, Aslam et al., Mol Biol Cell, 2009).

We thank the reviewer for this suggestion and added a corresponding section in our discussion, including the missing references:

These results complement its previously described role in mESC pluripotency exit [46] and align with the peri-gastrulation lethality observed in *Cnot8* knockout embryos, a

phenotype not seen in *Cnot7* knockout mice, which remain viable [46]. Importantly, prior work in non-embryonic systems indicate functional redundancy between CNOT7 and CNOT8, rendering the present observations particularly striking [69,70]. (**New References**).

Fig.S3N is not informative. Instead, it would be helpful to show the structure of the proteins (which have very similar/identical folds as shown) with highlighted the positions that are not identical/conserved/not-conserved.

Thank you for this comment, Figure S3N has now been revised to include an overlay of CNOT7 and CNOT8, highlighting their structural similarities and differences.

Ref.51 is dated; there are several more recent reviews to choose from that are up-to-date and include the concept that the Cnot9 subunit is used to recruit deadenylase activity via interactions with RNA-binding proteins.

Thank you for this suggestion, we now included the following more recent reviews as additional references:

41. Raisch, T. & Valkov, E. Regulation of the multisubunit CCR4-NOT deadenylase in the initiation of mRNA degradation. *Curr Opin Struct Biol* **77**, 102460 (2022).

42. Caulier, G., Siblini, J., Sène, L., Mauxion, F. & Séraphin, B. The CCR4–NOT complex: a multifaceted sensor of molecular signals instructing eukaryotic mRNA translation and stability. *Nucleic Acids Res* **53**, gkaf1401 (2025).

43. He, S., Valkov, E., Cheloufi, S. & Murn, J. The nexus between RNA-binding proteins and their effectors. *Nat Rev Genet* **24**, 276–294 (2023).

Fig.3B An over-representation of Cnot10 is observed in endoderm as well as an overrepresentation of Cnot9 in neural progenitors. Given that these are Ccr4-Not subunits, it would be helpful to comment briefly.

Thank you for raising this point and the suggestion to comment. *Cnot10* was overall depleted, rendering its overrepresentation in the endoderm non-significant due to the overall low number of sgCnot10 cells in our dataset.

We added the following section in the text:

"Other CCR4-NOT components included in the screen, such as *Cnot1* and *Cnot10*, exhibited strong overall depletion, consistent with their essential roles in the complex [41,50],

thereby precluding statistically significant germ layer–specific enrichment or depletion due to low cell numbers."

As additional information, we have in the meantime also updated our single-cell RNA-sequencing and CRISPR bioinformatics pipelines. To ensure consistency with the current analysis framework, which we plan to make publicly available on GitHub upon publication of this study, we reprocessed the present dataset using the updated pipeline. Consequently, we updated all affected panels, primarily Figures 1-3, resulting in minor changes to UMAP visualizations and differential gene expression analyses.

Reviewer #2 (Remarks to the Author):

Here, Taborsky et al. report a Perturb-Seq screen of ~200 genes in 3-dimensional mouse gastruloids, and they unexpectedly discover that deletion of RNA deadenylase Cnot8 leads to the emergence of notochord cells in gastruloids. This discovery is important in multiple ways. First, notochord cells are often lacking from conventional gastruloid models, and it is therefore very interesting that Cnot8 loss can enable their generation. Second, RNA deadenylation is often overlooked as a molecular mechanism that controls gastrulation and germ layer development, and the authors' work is thus quite exciting. Third, notochord cells in Cnot8-/- gastruloids express an impressive suite of archetypic markers, including Foxj1, Noto, Noggin, Chordin, Foxa2, and Brachyury, lending confidence that they are indeed notochordal in nature (Fig. 6).

While the authors do not fully resolve the molecular mechanism leading to this notochordal expansion, they show that Cnot8 deletion leads to an increase in both the poly(A) tail length and stability of the Nodal and Zic3 mRNAs. Stabilization of these two mRNAs, which encode notochord identity regulators, may contribute to the observed phenotype.

In any case, this is a strong and compelling paper that could be published in short order without fully resolving the molecular mechanism, at least in the opinion of this reviewer.

Novelty: This reviewer felt that the present study was original and novel. A previous study found that Cnot8-/- mouse embryos failed to complete gastrulation (with major defects seen by ~E7.5; https://www.ncbi.nlm.nih.gov/pubmed/35390160), but did not explore any potential axial mesoderm or notochord phenotypes. Reciprocally, recent studies have claimed to differentiate pluripotent stem cells towards notochord (https://pubmed.ncbi.nlm.nih.gov/39611739/, https://pubmed.ncbi.nlm.nih.gov/39695233/), but none of these studies invoked RNA deadenylation as a potential mechanism.

We thank the Reviewer for the insightful comments and advice and are especially grateful to the high level of detail, which has given us an opportunity to strengthen the presentation of our findings.

Major comments:
1. Further confirmation of notochord expansion in Cnot8-/- gastruloids:
a. In Fig. 6, it would be ideal if the authors could show 3 separate feature plots of notochord marker expression (e.g., of Noto) in control vs. Cnot7-/- vs. Cnot8-/- gastruloids. This would make it unequivocally clear that notochord markers are elevated in Cnot8-/- gastruloids, complementing the data in Fig. 6B and Fig. 6F.

Thank you for this helpful input, we now added a separate feature plot to Figure 6P (new Figure 5K) to make it clear that notochord markers are increased in *Cnot8* knockout gastruloids.

b. To support the compelling scRNA-seq data of expanded notochord cells in Fig. 6, it would be very nice if the authors could consider in situ staining or immunostaining for notochord markers, to back up their transcriptomic data. At present, they show increased Brachyury-P2A-mCherry signal in Fig. 4, but as the authors are aware, Brachyury is broadly expressed in multiple cell-types and therefore is not the most specific marker.

1. This is a great suggestion. We now carried out a set of immunostaining experiments for the notochord marker *Shh* in two *Cnot8* KO clones, control sgRNA gastruloids and the SBR line.

While *Shh* expression was basically absent in control sgRNA gastruloids and SBR gastruloids, we found a marked *Shh* signal in both *Cnot8* KO clones. We also quantified the maximal intensity across these immunostainings, confirming a clear increase in *Shh* in the *Cnot8* KO clones. We included these data to the new Figure 7.

2. In addition, to confirm that the observed gastruloid phenotype is specifically mediated by loss of CNOT8, we generated a *Cnot8* rescue construct and transduced both Cnot8 KO clones to establish stable lines. While the *Cnot8* KO clones exhibited a penetrant elongation defect, re-expression of CNOT8 largely rescued the phenotype. These data are now included in the new Figure 7.

2. Cell-type assignment of scRNA-seq clusters. Overall, this was a strong study, but this reviewer had questions about the cell-type assignment of several scRNA-seq clusters. These questions could be addressed by re-labeling clusters, or potentially re-clustering at higher resolution.
a. “Neuronal progenitor” cluster (Fig. 1B,C,H): Expresses pluripotency transcription Nanog and Pou5f1, as well as the neural crest/surface transcription factor Tfap2c

(https://pubmed.ncbi.nlm.nih.gov/17258188/). It may comprise a heterogeneous mixture of pluripotent and ectoderm cells. While this cluster expresses Tubb3, this gene is also expressed in other cell-types. Plotting the expression of neuronal markers Mapt and Snap25 may help to clarify the identity of this cluster.

Thank you for this interesting comment. Indeed, the presence of *Nanog/Pou5f1* suggests a mix of neuronal and undifferentiated cells, which we were unable to further resolve by additional markers. Therefore, we now adjusted the cluster label to reflect this limitation.

b. “NMP” cluster (Fig. 1B,C,H): Expresses Foxa2, which typically is not thought of as an NMP marker. Could this reflect Foxa2+ notochord or anterior primitive streak cells (https://pubmed.ncbi.nlm.nih.gov/8306889/) within this cluster?

This is an interesting point about the small population with higher Foxa2 expression within the NMP cluster. Indeed, we think that the ectopic notochord-like cells induced by loss of *Cnot8* or other perturbations in the screen may cluster with WT NMPs. Given the relatively low number of cells per perturbation, these cells are unlikely to form a distinct cluster and may instead be embedded within closely related populations.

c. “Cardiac mesoderm” cluster (Fig. 1B,C,H): Expresses Prrx1, which is a marker of limb bud progenitors, not heart progenitors (https://pubmed.ncbi.nlm.nih.gov/8101172/). Perhaps this cluster comprises a mixture of lateral mesoderm derivatives? To claim that this cluster corresponds to cardiac mesoderm, the authors should plot expression of the archetypic cardiac mesoderm transcription factor Nkx2.5.

We thank the reviewer for this important correction.

Our initial annotation was based on the expression of *Isl1*. However, further analysis as suggested by the reviewer revealed that *Nkx2.5* is expressed only in a subset of cells within this cluster (Figure S2C). This supports the interpretation that the cluster comprises a mixture of cardiac and other lateral plate mesoderm derivatives. We have therefore adjusted the cluster annotation accordingly.

d. “Differentiation front mesoderm” cluster (Fig. 1B,C,H): Are the authors referring to the anterior paraxial mesoderm where cells are undergoing segmentation? If so, assessing the expression of Mesp2 and other genes would be critical (https://pubmed.ncbi.nlm.nih.gov/9242490/). At present, the authors show that this cluster lacks Tbx6 and expresses Pax3, but more evidence is needed to show that these cells correspond to cells undergoing segmentation into somites, or the others should perhaps rename this cluster.

This is a great point. We were referring to the differentiation front described in gastruloids (PMID: 37137587; PMID: 31915373). Consistent with this interpretation, the cluster shows high expression of *Mesp2* and additional markers such as *Lfng*. To illustrate this point further, we have added the corresponding Nebulosa plots to Supplementary Figure S2C.

e. Fig. 6C: What is the rationale for the authors to distinguish “node” vs. “notochord” clusters?

We thank the reviewer for raising this question.

Our initial distinction was based on the co-expression of *T* and *Foxa2*, while more differentiated notochord markers, including *Noto*, *Chrd* and *Shh*, were not detected. Upon closer inspection, we agree that this distinction is not sufficiently supported, particularly in the absence of data on an anatomically defined node structure. We have therefore revised the annotation to “notochord progenitors,” based on pseudotime analysis and marker expression.

f. Fig. 6C: What is the rationale for the authors to annotate the “CLE” cluster as such?

We based this annotation on the pseudotime analysis, which positions this cluster upstream of the NMPs identified in the organoids, as well as on the expression of Cdx2 and Nkx1-2, together with low levels of T. However, we could also re-annotate this cluster as Epiblast II.

g. Fig. 6C: A cluster is labeled as “neural crest”, but showing the expression of distinctive markers Sox9, Sox10, or Wnt1 would be helpful.

We initially based this annotation on the co-expression of neuronal markers and markers associated with EMT. However, we were unable to substantiate this assignment using the markers suggested by the reviewer, potentially due to limited sequencing depth. Given the concurrent expression of pluripotency and neuronal markers, we have revised the label to “Epiblast/Neuronal progenitors II.” A more precise annotation would likely require additional single-cell data, which we consider beyond the scope of the present study.

3. Potentially swapping the order of Figs. 5 and 6. In Fig. 4, the authors present preliminary evidence for increased notochord identity in Cnot8-/- gastruloids (Fig. 4G). However, Fig. 4G is not easy to interpret: it shows differential gene expression in Cnot8-/- vs. Cnot7-/- gastruloids, but ideally one would focus on Cnot8-/- vs. control gastruloids. After Fig. 4, it might be better for the authors to directly proceed to Fig. 6 (which presents very strong proof for expanded notochord cells), and then to close the manuscript with Fig. 5 (molecular mechanism).

We thank the reviewer for this very helpful suggestion.

After comparing both figure orders and the flow, we agree with reviewer that swapping Figures 5 and 6 indeed improves the logical flow of the manuscript. We think that moving directly from Figure 4 to the validation of notochord identity (now Figure 5) and subsequently to the molecular mechanisms (now Figure 6) strengthens the narrative. We therefore have updated the text, figures, figure legends, supplementary figures (rearranging Figures S5-7) and tables (S4-S7) accordingly.

Minor comments:
1. “Despite their promise, the application of these techniques to the embryonic system” (Introduction) — would it be clearer to replace “embryonic system” with “embryo” here?

We agree with the reviewer, the text has been adjusted accordingly.

2. “sgRNAs targeting the germ layer controls Eomes and Sox17 showed endodermal depletion (Figure 2E and 3B), while T/Brachyury and Snai1 sgRNAs were depleted in mesodermal lineages (Figure 2E), validating the high specificity of our screening strategy.” (Results) – Fig. 2E is a beautiful figure, but each row is too thin for the reader to interpret. The authors could consider showing in the supplemental figures a larger figure focusing on only a few positive control sgRNAs.

Thank you for the comment. We now included a new panel in Figure S3C with a selection of genes of interest and controls.

3. In the main text, the authors should consider citing the figure panels in order. At the end of pg. 6, the authors cite Fig. 2J, but at the beginning of pg. 7, Fig. 3D is mentioned next.

We now adjusted the text and, whenever possible, cite the figure panels in order.

4. Fig. 3B — In the Perturb-Seq screen, it seems that Cnot8 is required for endoderm induction (log2 fold change = 0.75). However, in Fig. 6E, when the authors perform detailed analysis of the Cnot8-/- gastruloids, there is now a modest increase in endoderm induction. Can the authors reconcile these two? This is a minor comment, because of course one would believe the results from detailed follow-up more than the screening results. In any case, would it be helpful for the Perturb-Seq screen to show the distribution of non-targeting sgRNAs? Maybe the variability in scores seen in non-targeting sgRNAs would encompass the endoderm score of Cnot8, implying that it was not statistically significant in the endoderm readout of the screen?

Thank you for this comment and the opportunity to clarify.

We think that direct comparison between the screen dataset and the full knockout is difficult, as the organoids in the screen are chimeras composed of majorly wild-type (uninfected) cells, with only about 6% cells carrying an sgRNA of which only a minority carries a knockout for *Cnot8*. Given that the phenotype is likely mediated through altered signaling, the presence of surrounding wild-type cells may modulate the observed effects. Thus, organoids consisting entirely of *Cnot8* KO cells are expected to behave differently from chimeras, potentially explaining the difference in endoderm percentage.

We have clarified this point in the manuscript and added corresponding analyses in Figure S2J-K, illustrating control sgRNA distributions and *Cnot8* sgRNA enrichment/depletion across endoderm and mesoderm in both replicates.

5. Fig. 3G — This figure displays scRNAseq expression of genes in their gastruloids. Is Cnot8 mRNA also decreased in axial mesoderm and notochord cells in vivo? scRNAseq datasets of the gastrulating mouse embryo are available: https://pubmed.ncbi.nlm.nih.gov/30787436/ and https://crukci.shinyapps.io/mousegastrulation2018/.

Thank you for this suggestion. There appears to be no significant decrease of *Cnot8* expression in axial mesoderm and notochord cells *in vivo* using the mouse embryo data, with *Cnot8* being relatively ubiquitously across cell types and time points.

6. Fig. 3E — If the reviewer understands this correctly, sgRNA deletion of T increased the number of NMPs? Is this surprising, given the central role of Brachyury/T in NMP development (https://pubmed.ncbi.nlm.nih.gov/26329597/)? Apologies if the reviewer did not understand this figure panel.

We agree with the reviewer that this is indeed an unexpected finding. The reviewer is correct that T perturbations were the top enriched sgRNAs in NMPs. Our current interpretation is that *sgT* cells may adopt an NMP-like state, allowing them to cluster with NMPs, but fail to properly transition out of this state. However, a detailed mechanistic understanding of this phenotype in gastruloid NMPs would require additional experiments, which we consider beyond the scope of the present study.

7. Fig. 4B — The authors should consider re-labeling the x-axis from “mCherry” to “T-P2A-mCherry”.

We have re-labeled the x-axis accordingly.

8. Fig. 4F — It would be terrific if the authors could label this cartoon to specify that T+ cells were isolated, so readers know what purple signifies.

We have specified in the cartoon that T+ cells were isolated.

9. Fig. 4G could be modified or replaced to make it easier to interpret.
a. To support the claim that Cnot8 deletion leads to an increase in notochord markers, the authors would have to show that notochord markers (e.g., Noto) are upregulated in Cnot8-/- gastruloids vs. control gastruloids. Instead, in Fig. 4G, the authors compare Cnot8-/- vs. Cnot7-/- gastruloids. A volcano plot of Cnot8-/- vs. control gene expression is shown in Fig. S6E, but notochord markers were not highlighted. It would be ideal if the authors could show two separate volcano plots comparing Cnot8-/- vs. wild-type, and separately, Cnot7-/- vs. wild-type.

We agree with the reviewer. However, one control replicate did not cluster with the other control samples or the *Cnot7* knockout gastruloids and was therefore excluded from the analysis shown in Figure 4. In our hands, *Cnot7* knockouts largely resemble control gastruloids, with only minimal differential gene expression (Figure 3H), and we compare *Cnot7, Cnot8 and Cnot9* to wild-type gastruloid cells in Figure 3.

For Figure 4, we were unfortunately unable to repeat the sorting and bulk RNA-sequencing experiment within the revision timeframe and therefore present a direct comparison between *Cnot7* and *Cnot8* to highlight the differences in gene expression.

However, importantly, we have now included rescue experiments demonstrating that re-expression of CNOT8 reverses notochord marker expression in Cnot8 knockout gastruloids. We included these new experiments in Figure 7A-D.

b. In volcano plots, ideally the authors could insert labels to indicate whether the “left” or “right” of the plot signifies that a gene is upregulated (or is downregulated) in a specific genotype.

We have now updated all volcano plots in the main figures (Figures 1–7) to clearly indicate the directionality, specifying what the left and right sides represent and in which genotype genes are up- or downregulated.

10. Fig. 6D — It would be helpful if the gene labels are spaced out more, so the reader can understand which feature plot corresponds to which gene. For instance, the left-most plot is intersected by both “T” and “Cdx2” text boxes.

We now adjusted the spacing and added dashed lines to make the link between plot and gene clearer. For consistency we used the same approach in Figure 1C.

11. “Additionally, notochord differentiation in a human model of trunk development has only recently been achieved under highly specialized culture conditions (49).” — It may be worth

also citing a second paper on the similar subject here (https://pubmed.ncbi.nlm.nih.gov/39611739/).

We now cite the second paper.

12. "BMP inhibitors Chrd, Nog, and Shh" — The authors should consider editing the phrasing to clarify that Shh is not a BMP inhibitor.

We adjusted the section as follows:

Further analysis of the putative notochord cluster revealed the presence of notochord-associated signaling factors, including BMP inhibitors *Chrd*, *Nog*, the hedgehog ligand *Shh*, as well as the cilia-related genes *Tppp3* and *Fam183b* (Figure 6F-G).

13. "This transcriptional profile suggests that these notochord cells represent a functional notochord akin to the embryonic equivalent" — Given that notochord functionality was not tested in this report, the adjective "functional" should be removed here.

Thank you for this comment. We agree, the lack of functional assessment makes any such claims speculative and we removed the sentence.

14. Fig. 6C — The "paraxial" and "somitic" labels are scrunched up and are a bit hard to read.

We adjusted the labeling to increase the readability.

15. Fig. 6P — This is a very nice summary cartoon, but the authors may want to revisit it. The cartoon indicates that anterior regions of the embryo are specified by high Nodal and low Wnt, but as the authors are aware, it is more complicated, since high Nodal specifies the anterior primitive streak, but subsequently it seems that Nodal must likely be inhibited to specify notochord identity (https://pubmed.ncbi.nlm.nih.gov/39611739/, https://pubmed.ncbi.nlm.nih.gov/39695233/). It is definitive endoderm that requires high and sustained Nodal signaling for its specification. Since it is difficult to depict these temporally- and spatially-dynamic signaling domains on a static photo of the embryo, maybe the authors could consider alternative cartoons, like a lineage roadmap, where it is easier to label which lineages are induced by what signals.

This is a great suggestion, thank you for this idea.

As suggested, we now included a lineage roadmap to contrast wild-type and *Cnot8* knockout lineages and the underlying signaling in Figure 7E.

16. If Nodal and Zic3 transcript stability is improved by Cnot8 deletion, are these mRNAs also elevated in their RNA-seq datasets?

Thank you for raising this point.

Revisiting our bulk RNA-seq data from 72 h and 96 h gastruloids does not reveal a significant increase in Zic3 or Nodal expression. This may be partly due to the limited sample size, the overall low expression levels of these factors and the lack of temporal resolution in our dataset. It is possible that *Zic3* stabilization occurs at a specific time point or within a subset of cells that cannot be captured by bulk RNA-seq. A more finely resolved temporal analysis of *Cnot8* knockout gastruloid development would be required to address this, which is unfortunately beyond the scope of the present study.

As additional information, we have in the meantime also updated our single-cell RNA-sequencing and CRISPR bioinformatics pipelines. To ensure consistency with the current analysis framework, which we plan to make publicly available on GitHub upon publication of this study, we reprocessed the present dataset using the updated pipeline. Consequently, we updated all affected panels, primarily Figures 1-3, resulting in minor changes to UMAP visualizations and differential gene expression analyses.

Reviewer #3 (Remarks to the Author):

In this manuscript, Taborsky et al conduct a perturb-seq screen focused on post transcriptional regulators in mouse gastruloids to identify candidates that influence germ layer fate. They focus on Cnot8, a deadynlase whose KO surprisingly leads to the generation of notochord cells. They characterize the Cnot8 KO mutant and find that Cnot8 is involved in transcript stability of several genes that are important for embryonic patterning.

This manuscript incorporates a lot of different genomic techniques (high throughput screening, long read sequencing, ribosome profiling, etc) with a hypothesis driven design resulting in a satisfying understanding of how post-transcriptional regulation through Cnot8 deadenylation affects cell fate. This work includes useful data resources for the community including the CROP-seq screens and the translation atlas for mouse gastruloids among others. The experiments and analysis are conducted well with appropriate controls. The manuscript is well written with clear logic and is easy to follow.

We appreciate the Reviewer's support and the very helpful suggestions that have strengthened the manuscript.

Comments:

1. Reproducibility of screen results
The authors perform CROP-seq screens targeting 243 genes with two replicates. It is not entirely clear how the screen is carried out, for example: is each replicate composed of a single gastruloid or does the screen take place across several gastruloids? What is the coverage, i.e. how many cells are infected by each gRNA? Most importantly, how reproducible are the screen results? The authors need to show a scatterplot of the log fold change reported by each screen. Points can be either gRNAs or genes (where the value is the avg across gRNAs) with a correlation coefficient to measure reproducibility. This is a valuable measurement for readers to understand how well these types of screens perform where the initial population undergoes stratification towards different outcomes.

We thank the reviewer for these valuable suggestions and agree that the original manuscript lacked clarity on these points.

1. We have revised the corresponding section to include coverage metrics and a more detailed description:

"We harvested ten 96-well plates of gastruloids from two independent cultures at 120 h post-aggregation and subjected them to single-cell RNA sequencing (scRNA-seq) with sgRNA capture. Following doublet removal, filtering, and sgRNA annotation, 135,392 cells were retained, corresponding to a median coverage of 201 cells per sgRNA."

Of note, the coverage is comparable to our previous in vivo CROP-seq screen (PMID: 39020166; 240 cells per guide in P4 and 366 in P60) and other Perturb-seq studies (PMID: 33243861). Nevertheless, we note that, given the transcriptomic resolution and sequencing depth required for scRNA-seq, coverage in these CROP-seq and perturb-seq experiments is generally lower than in conventional pooled CRISPR screens, primarily due to budget constraints. This limitation, however, is offset by the single-cell transcriptomic resolution provided by the approach.

2. As suggested by the reviewer, we now included for the reader a scatterplot demonstrating the reproducibility of the screen between the two replicates at the gene level in Figure 2A (right panel), and added a corresponding reference in the text:

As expected, we found a strong and reproducible depletion of *Eif1*, *Rps6, Eef2* or *Rpl10a* perturbed cells across gastruloid development (Figure 2A, C).

In general, we find a solid correlation between the two major replicates (Figure 2A). However, compared to *in vitro* screens that primarily measure proliferation effects within a single cell type, the increased cellular and lineage complexities of gastruloids, encompassing multiple differentiation trajectories, is expected to lead to lower overall correlation coefficients.

Given this complexity, we performed rather extensive validation of our screening results using orthogonal assays and independent knockout lines. In addition, we now included rescue experiments demonstrating that re-expression of wild-type CNOT8 restores the *Cnot8* mutant phenotype (Figure 7).

Finally, we now also assess the developmental potential of *Cnot8* knockout ESCs *in vivo.* To this end, we generated chimeric embryos by injecting GFP-labelled *Cnot8* knockout ESCs into blastocysts. Although further studies will be required to define the abnormalities in more detail, our findings suggest that the presence of *Cnot8* knockout cells in chimeric embryos may be associated with developmental abnormalities. We included these experiments in Figure S7N-O.

2. Cnot8 KO notochord cells in Figure 4
The authors observe an unusual phenotype in the Cnot8 KO gastruloids – the T-P2A-mCh cells do not localize to the posterior pole. They isolate these cells using FACS and perform bulk RNA-seq and conduct DEG analysis and GO enrichment to claim these cells have notochord phenotype instead of the expected NMP phenotype. Will the authors please compare the transcriptome profile from the Cnot8 KO gastruloids T-positive population directly with the notochord signature from mouse embryos and quantify their similarity? At this point in the manuscript, the authors have established the atlas data from Figure 1. I believe a comparison at this stage would be complementary to the analysis in Figure 6 because there is some location information provided by imaging. Also, Cnot8 KO cells appear in the crop-seq screen. Will the authors please comment on whether the notochord phenotype was apparent in the screen?

We thank the reviewer for raising these insightful points.

1. We now compared the transcriptome profile from the Cnot8 KO gastruloids T-positive population directly with the notochord signature from mouse embryos. Although comparing bulk-RNA seq with scRNAseq has its caveats, we created a pseudobulk count file of embryo "notochord" cells (assuming the annotations are largely correct) and compared them to *Cnot8* KO T-positive gastruloids. While one *Cnot8* knockout clone clustered with E8.25 notochord cells, the other *Cnot8* knockout clone did not, leaving the findings difficult to interpret conclusively (Reviewer figure).

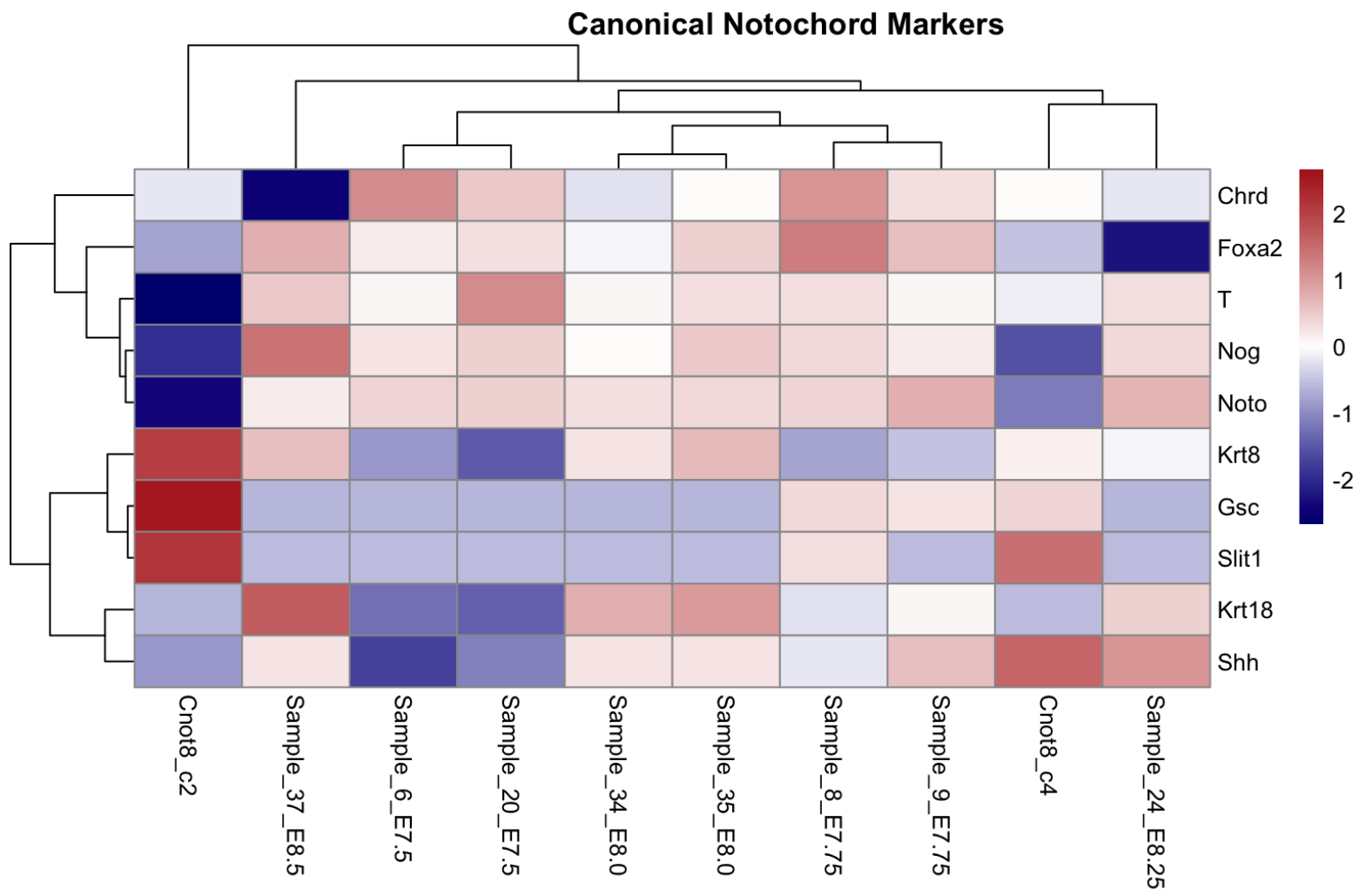

2. This is a great point and now also examined the *Cnot8*-perturbed cells in the CROP-seq screen. While *Cnot8* KO cells showed increased expression of notochord markers in the screen data, these changes were largely not statistically significant. We attribute this to the relatively low number of perturbed cells, potential masking by other cell types within the organoids, and the presence of unedited or heterozygous KO cells in the population.

However, to further validate the presence of notochord-like cells in *Cnot8* knockout gastruloids, we directly assessed notochord marker expression. Immunostaining for the notochord marker *Shh* revealed no detectable signal in wild-type or control sgRNA gastruloids, whereas both *Cnot8* knockout clones exhibited a pronounced *Shh* signal. These new experiments are included in Figure 7. Quantification of maximal staining intensity confirmed a clear increase in Shh levels in the *Cnot8* knockout clones (Figure 7B). To verify that these phenotypes are specifically attributable to *Cnot8* depletion, we re-expressed CNOT8 in both knockout clones. While *Cnot8* knockout gastruloids displayed the expected strong elongation defect, re-expression of CNOT8 largely restored normal elongation, supporting a direct role for CNOT8 in regulating this developmental phenotype (Figure 7C-D).

3. Other cell types enriched in Cnot8 KO
Inspection of Fig 6E and Supp Fig 7A reveals that “Node” also appears in addition to notochord upon KO of Cnot8. Will the authors please add a comment into the manuscript that addresses its presence because at present, it is a glaring omission. Also, it appears the neural progenitors produced in the Cnot8 KO are different from those in WT or the Cnot7 KO. Will the authors perform a DEG analysis for the neural progenitors partitioned by Cnot8KO vs rest to examine if meaningful differences exist between the two populations?

Thank you for raising these valid points.

1. In line with the concerns raised also by Reviewer 2, we re-examined the node classification. A more detailed analysis of the “node-like” population led us to reassign it as early notochord progenitors. Although this cluster exhibits expression of markers overlapping between notochord and node, these are not sufficient to support a bona fide node identity without accompanying anatomical evidence. We have therefore updated the annotation to “notochord progenitors” and apologize for any confusion.

2. The reviewer is correct regarding the point about neuronal progenitors. Our neuronal progenitor cluster does express pluripotency markers suggesting a very early state of neuronal specification, intermixed with pluripotent stem cells. Taking this caveat into account, *Cnot8* KO and control neuronal progenitors indeed appear to be clustering differentially. Cnot8 KO cells show higher expression of neuronal markers (e.g. *Tubb3*), indicating a more differentiated phenotype compared to *Cnot7* KO and control. While both populations include a mix of early neuronal and undifferentiated cells, *Cnot8* KO “neuronal progenitors” appear more mature overall. We have added a section to the manuscript to reflect this important point:

The upregulation of neuronal markers, including *Tubb3* could be indicative of increased maturity of the neuronal progenitor cells found in the organoids, even though the overall immature state of these cells limits our ability to make definitive conclusions regarding their differentiation.

Minor points:
- Fig 2E is not informative and should be replotted with each gene/sgRNA against the sgRNA control instead of raw cell type proportions. If the visualization is kept, then consider ordering the cell types so that paraxial meso is either on top or bottom of the column.

Thank you for this good suggestion. We now adjusted Figure 2E and re-ordered the panel as suggested so that the paraxial mesoderm is now at the bottom of the column.

- Fig 3D: “UpSet” not upset.

The typo has been fixed.

- Fig 3G: Statistical test to support claim that Cnot8 is “markedly reduced”.

This is a valid point to bolster this statement.

To substantiate this claim, we now run a Wilcoxon rank-sum test to specifically compare *Cnot8* expression in the different clusters compared to the epiblast population. (*Cnot7/8/9*

expression was comparable in the epiblast). We found that *Cnot8* was significantly reduced in paraxial and somitic mesoderm compared to the epiblast.

We have now also converted this panel to a dot plot to better illustrate expression levels and have included the corresponding statistical analysis in Figure 3G.

- In the scRNA-seq gastruloid data (Figure 5), why are there so many more control cells that passed filter for analysis than KO cells? From the text, it appears that the same number of gastruloids went into the pipeline. Were the KO gastruloids smaller or was there a technical issue for the KO scRNA-seq libraries?

Thank you for the opportunity to clarify this point.

Single cells from equal numbers of gastruloids derived from *Cnot7 KO*, *Cnot8 KO,* and control organoids were pooled at equal ratios and loaded onto a single BD Rhapsody cartridge. Cell lines were subsequently demultiplexed using sgRNA identities. Following demultiplexing, one *Cnot7* and one *Cnot8* population were underrepresented, likely reflecting technical variation during sample preparation, such as for example incomplete dissociation of the gastruloids. We now have revised the relevant section of the manuscript to provide additional detail for the reader:

Organoids showed comparable size, cell viability and appeared healthy, while recapitulating the described *Cnot8* knockout phenotype. For each condition, we harvested 16 gastruloids, dissociated the organoids and pooled single cells in equal ratios and subjected to scRNA-seq. After removal of doublets, low-quality and dead cells, 16,966 cells were retained for analysis, consisting of 4,873 *Cnot7* knockout, 4,858 *Cnot8* knockout and 7,235 control cells.

- Supp Fig 7A does not have a key to map colors to cell types

Thank you for raising this issue. We now have transferred the cell type annotations to match Figure 6A (new Figure 5A).

As additional information, we have in the meantime also updated our single-cell RNA-sequencing and CRISPR bioinformatics pipelines. To ensure consistency with the current analysis framework, which we plan to make publicly available on GitHub upon publication of this study, we reprocessed the present dataset using the updated pipeline. Consequently, we updated all affected panels, primarily Figures 1-3, resulting in minor changes to UMAP visualizations and differential gene expression analyses.
