# Supplementary material for: Regulation of mRNA poly(A) tail length governs mammalian body plan formation in gastruloids
Source: Nat Commun. 2026 Aug 29;17:10390. doi: 10.1038/s41467-026-76897-3 (PMC13627704; doi:10.1038/s41467-026-76897-3)
Supplement: Supplementary file 3 [file 41467_2026_76897_MOESM1_ESM.pdf]

Supplementary Figure 1

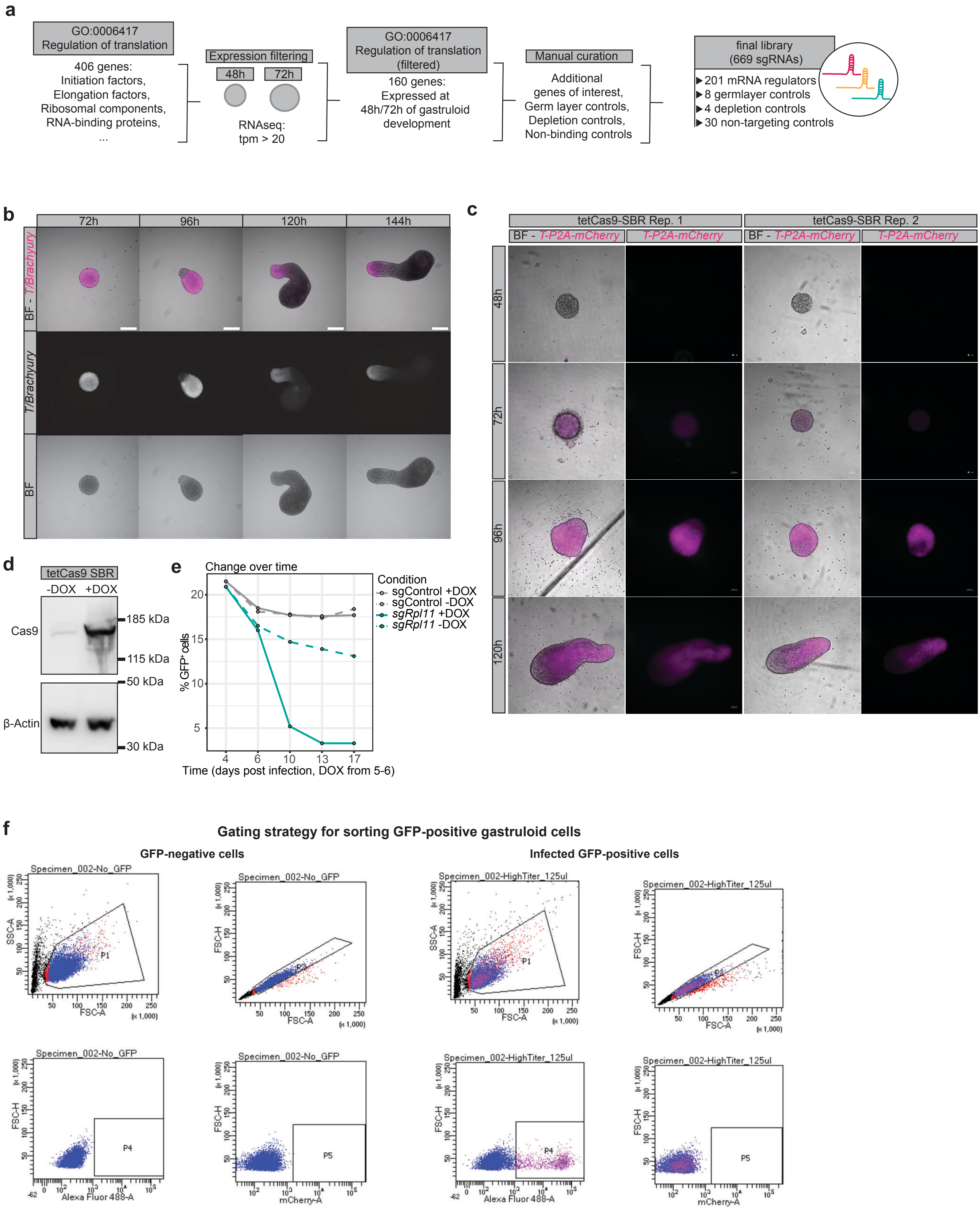

Supplementary Figure 1: Establishing a tetCas9 inducible murine embryonic stem cell (mESC) line in the SBR background.

(a) Outline of target gene selection. Genes annotated to the GO term "Regulation of translation (GO:0006417)" were filtered by expression in 48 h and 72 h gastruloids. The resulting list was then manually curated by adding germ layer controls, depletion controls and nontargeting controls for a final library of 669 sgRNAs.

(b) T/Brachyury-mCherry, SOX1-GFP reporter (SBR) mESC-derived gastruloids stained for T/Brachyury at different time points. Scale bar, 200 μm.

(c) Images of tetCas9 SBR-derived gastruloids during 48, 72, 96 and 120 h of gastruloid development.

(d) Western blot of CAS9 expression in tetCas9-SBR cells upon 48 h of 2 μM doxycycline treatment.

(e) Flow cytometry data of tetCas9 SBR cells transduced with sgRNAs targeting *Rpl11* (*sgRpl11*) and nontargeting controls (sgControl), with or without doxycycline induction.

(f) Representative gating strategy for sorting GFP-positive gastruloid cells.

Supplementary Figure 2

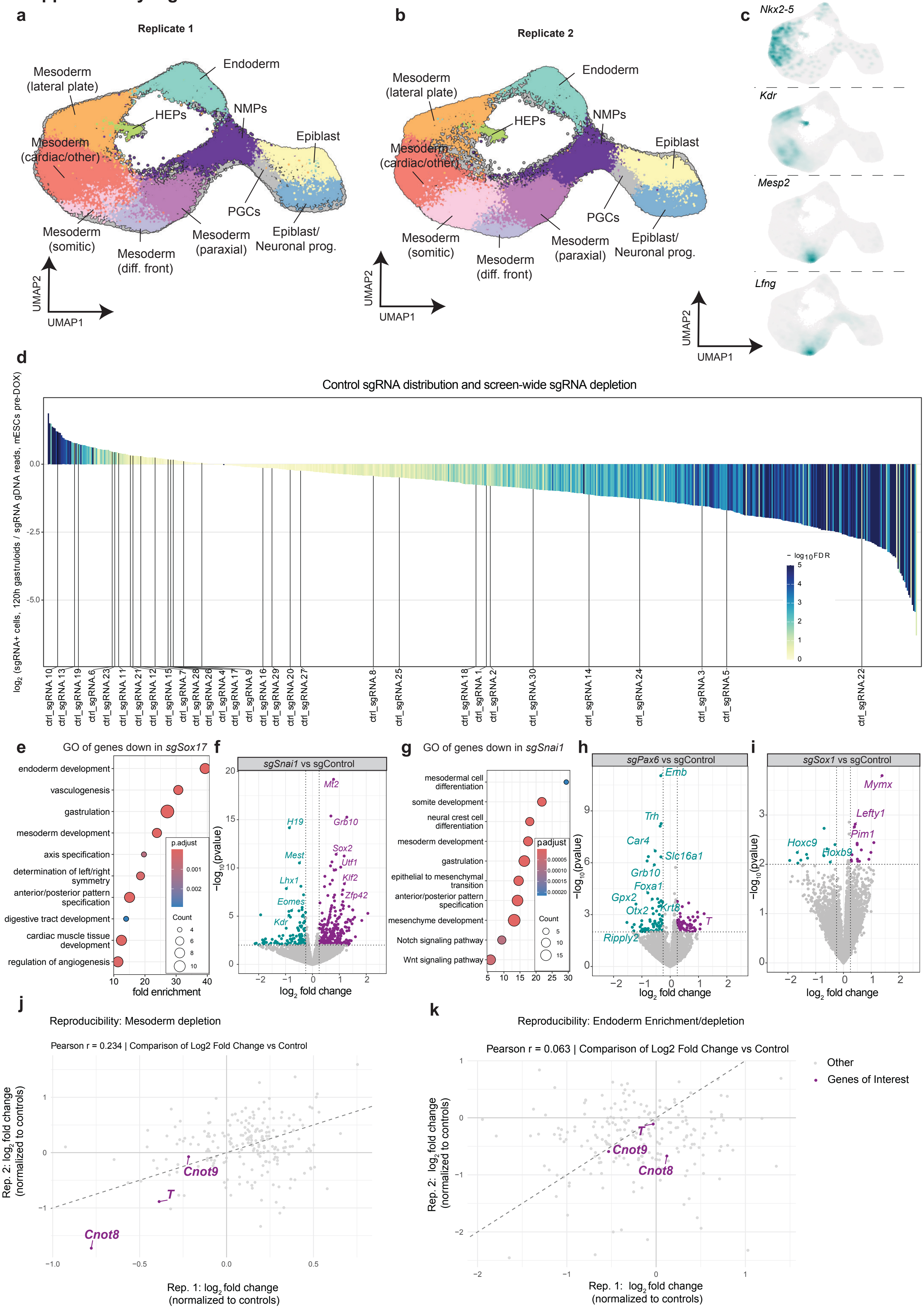

Supplementary Figure 2: Screen reproducibility and germ layer controls.

(a-b) UMAP showing cell type distribution of 120 h gastruloids from two independent screen replicates.

(c) Nebulosa plot showing expression of additional marker genes.

(d) Waterfall plot showing enrichment and depletion of 669 sgRNAs, comparing pre-doxycycline treatment with 120 h gastruloids, with nontargeting control sgRNAs labelled.

(e) GO term enrichment of genes downregulated (p-value < 0.01) in all *sgSox17* cells.

(f) Volcano plot displaying the top differentially expressed genes in 120 h gastruloids upon perturbation of *Snai1* (*sgSnai1*).

(g) GO term enrichment of genes downregulated (p-value < 0.01) in all *sgSnai1* cells.

(h) Volcano plot displaying the top differentially expressed genes in 120 h gastruloids upon perturbation of *Pax6* (*sgPax6*).

(i) Volcano plot displaying the top differentially expressed genes in 120 h gastruloids upon perturbation of *Sox1* (*sgSox1*).

(j, k) Scatterplots demonstrating the reproducibility of sgRNA enrichment and depletion (gene level) between screen replicates in mesoderm (j) and endoderm (k). Axes represent the $\log_2$ fold change of individual perturbations relative to pooled non-targeting controls for replicate 1 (x-axis) and replicate 2 (y-axis). *Cnot8* and *Cnot9* are highlighted in magenta. The dashed line represents perfect correlation. Pearson correlation coefficient (r) is indicated for each comparison.

Supplementary Figure 3

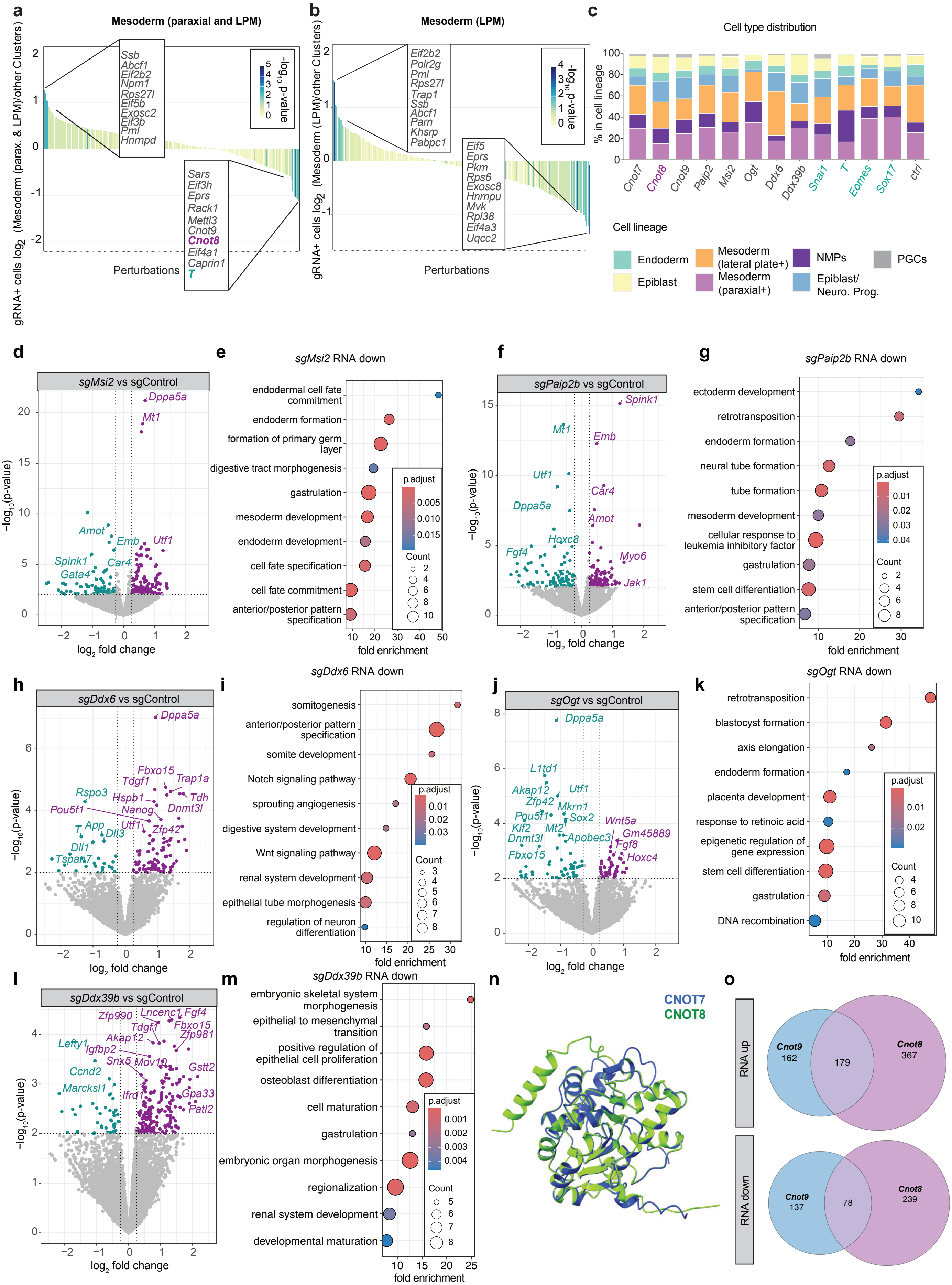

Supplementary Figure 3: Identification of additional novel regulators of germ layer differentiation in 120 h gastruloids.

(a-b) Waterfall plots displaying the changes in sgRNA representation in combined paraxial and LPM mesoderm (a) and lateral plate mesoderm (b).

(c) Higher magnification of Figure 2e, highlighting candidates, germ layer controls and nontargeting controls.

(d-m) Volcano plot and GO term analysis of additional candidates including endodermally depleted *Msi2* (*sgMsi2*) (d-e), neuromesodermally depleted *Paip2b* (*sgPaip2b*) (f-g), globally depleted *Ddx6* (*sgDdx6*) (h-i), neuronally depleted *Ogt* (*sgOgt*) (j-k) and globally depleted *Ddx39b* (*sgDdx39b*) (l-m).

(n) Alphafold structure of CNOT7 and CNOT8.

(o) Venn diagram of genes significantly upregulated (top panel) or downregulated (bottom panel) in *sgCnot8* and *sgCnot9* cells.

Supplementary Figure 4

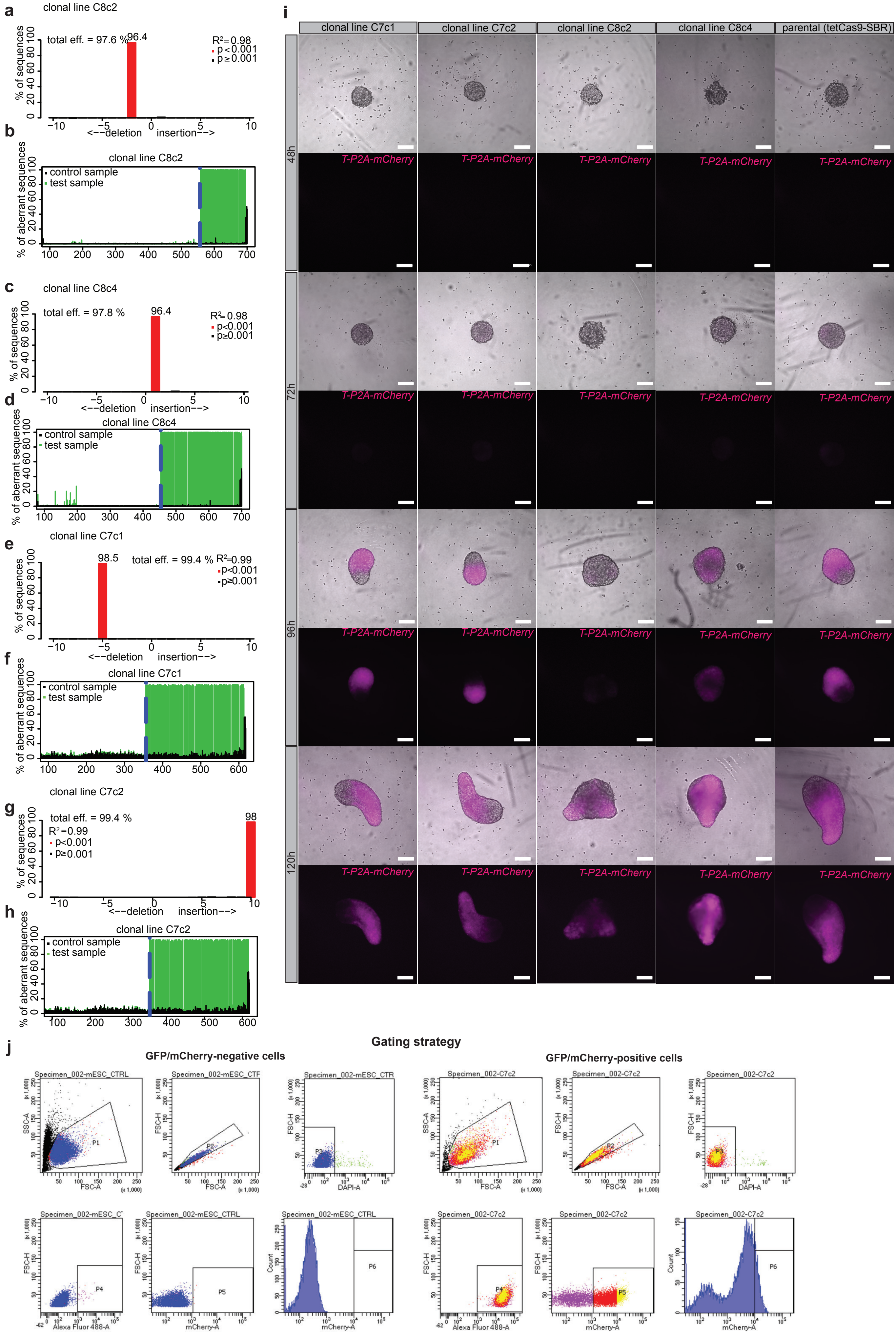

Supplementary Figure 4: Establishing *Cnot7* and *Cnot8* knockout clonal cell lines.

(a-h) Tide assay results for 2 *Cnot8* knockout (a-d) and 2 *Cnot7* knockout clonal cell lines (e-h).

(i) Time-lapse images of gastruloids from 2 *Cnot7* clonal cell lines, 2 *Cnot8* clonal cell lines, alongside the parental control lines (tetCas9-SBR).

(j) Representative gating strategy for sorting GFP-positive gastruloid cells.

Supplementary Figure 5

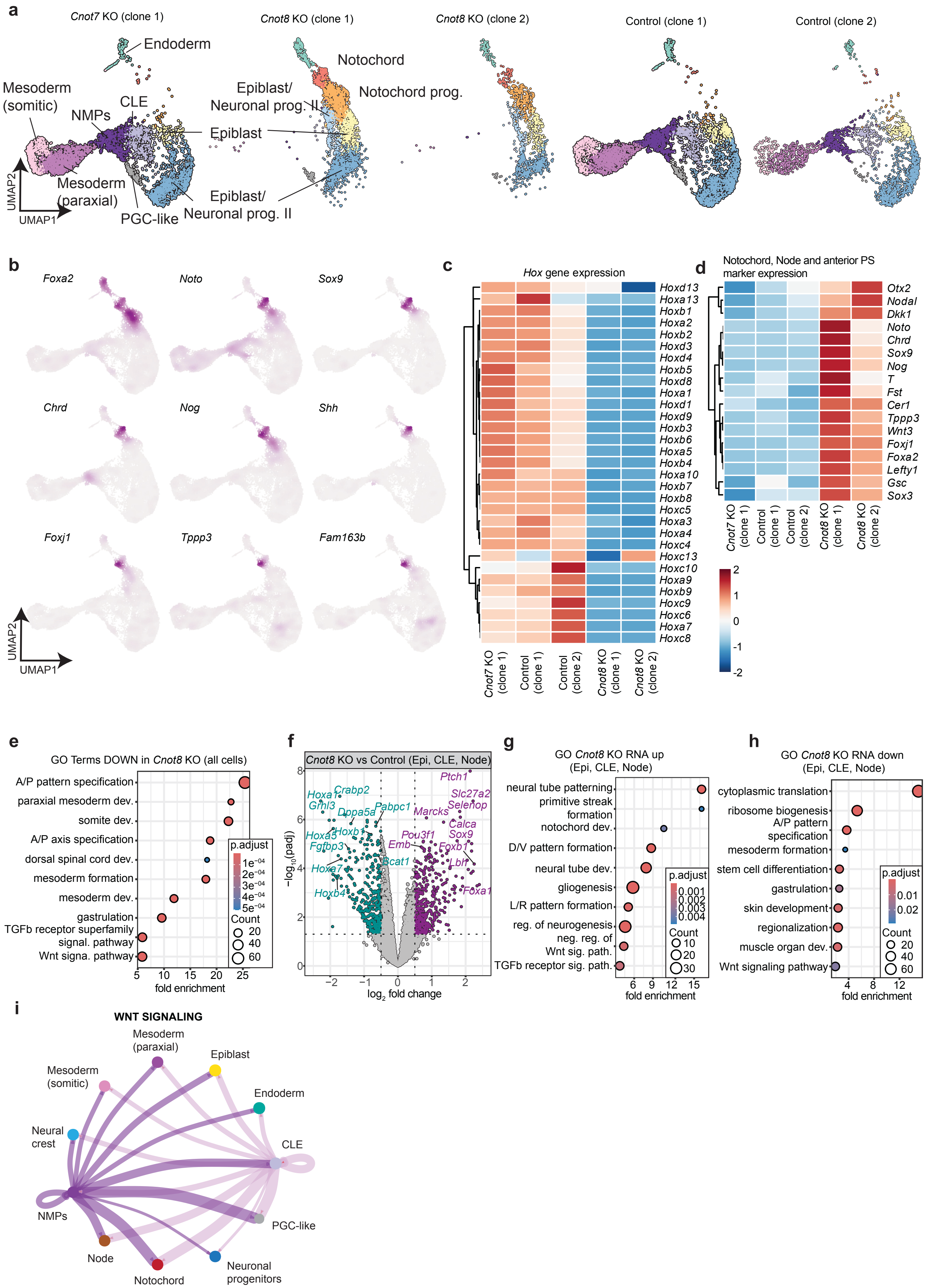

Supplementary Figure 5: Single-cell RNA sequencing in *Cnot8* KO reveals altered differentiation trajectories.

(a) UMAP showing cell type distribution of individual clonal cell lines.

(b) Full-size nebulosa density plots for the notochord markers *Foxa2*, *Noto*, *Sox9*, *Chrd*, *Nog*, *Shh*, *Foxj1*, *Tppp3* and *Fam183b.*

(c) Heatmap of *Hox* gene expression in *Cnot7* knockout, *Cnot8* knockout and control gastruloids.

(d) Heatmap of markers of the node, the notochord and anterior primitive streak (PS).

(e) GO term enrichment of genes downregulated (p-adj < 0.05, $\log_2$ fold change < -1) in all *Cnot8* knockout cells.

(f-h) Volcano plot (f) and GO enrichment analysis (g-h) in progenitor clusters (Epiblast, node, caudo-lateral epiblast (CLE)) in *Cnot8* KO cells versus control cells, revealing induction of notochord development and left-right (L/R) pattern formation.

(i) CellChat analysis of Wnt signaling across the entire dataset (*Cnot7* KO, *Cnot8* KO, control) identifies NMPs and CLE as major signaling sources.

Supplementary Figure 6

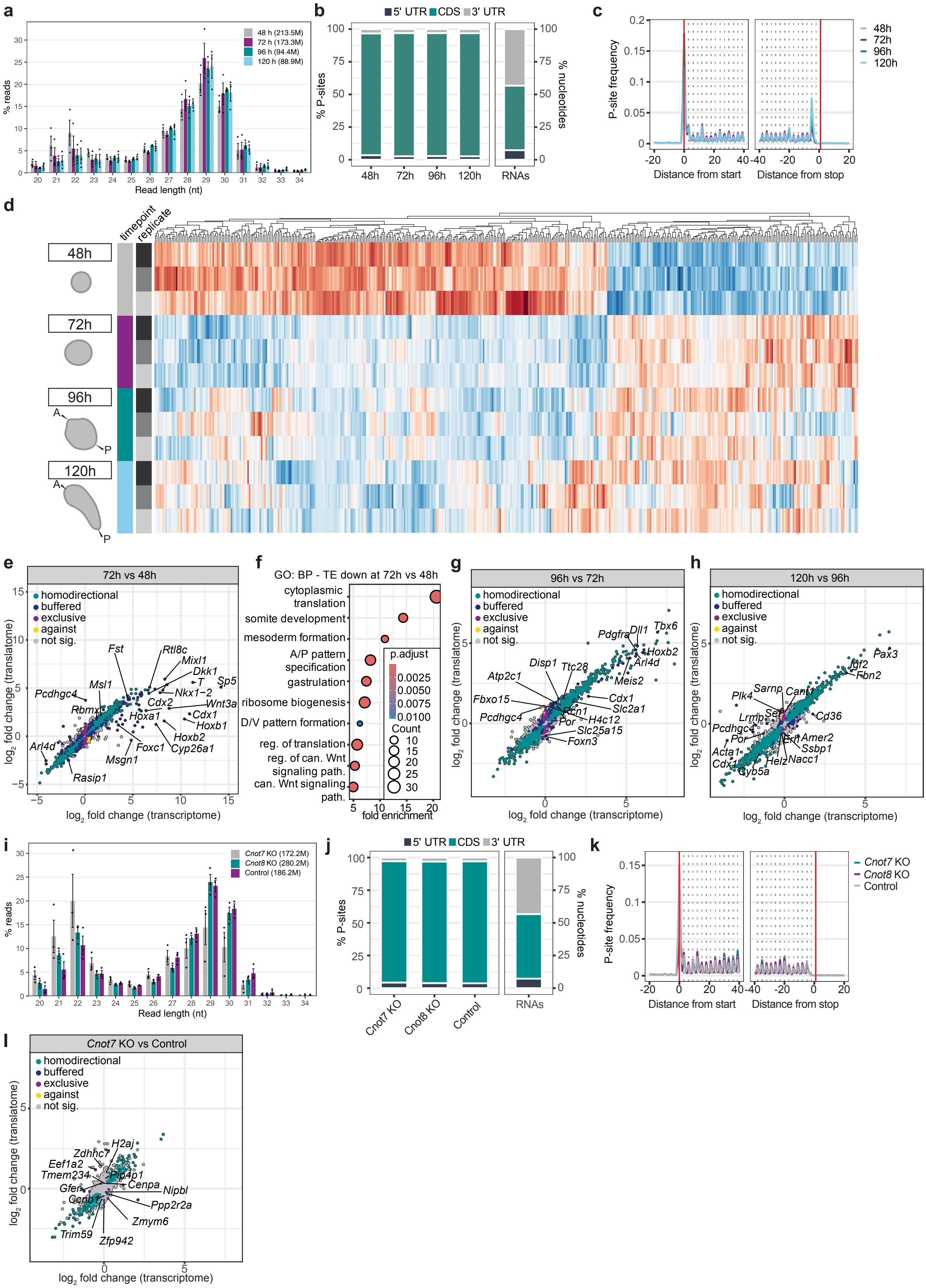

Supplementary Figure 6: Establishing the translational landscape of gastruloids.

(a) Fragment length distribution of ribosome profiling samples from 48 h, 72 h, 96 h and 120 h gastruloids. n = 3 biological replicates per timepoint (reads within 20–34 nt: 48 h 213.5M, 72 h 173.3M, 96 h 94.4M, 120 h 88.9M). Bars, mean; error bars, SEM; points, individual replicates.

(b) P-site distribution of ribosome profiling samples from 48 h, 72 h, 96 h and 120 h gastruloids shows enrichment in the coding sequence (CDS).

(c) Periodicity plot showing triplet periodicity in ribosome profiling samples from 48 h, 72 h, 96 h and 120 h gastruloids.

(d) Heatmap showing the translational efficiency (TE) of 483 genes with significantly altered TE between 72 h and 48 h of gastruloid development.

(e-h) Scatter plot showing the $\log_2$ fold changes in transcript abundance and ribosome occupancy (translatome) of (e) 72 h versus 48 h, (g) 96 h versus 72 h (h) and 120 h versus 96 h gastruloids. Genes regulated exclusively at the translational level are shown in magenta. GO terms enriched among genes with decreased translational efficiency (TE) at 72 h versus 48 h of gastruloid development are shown in (f).

(i) Fragment length distribution of ribosome profiling samples from *Cnot7* KO*, Cnot8 KO* and control gastruloids at 72 h. n = 3 biological replicates per condition (total mapped reads: Cnot7 KO 172.2M, Cnot8 KO 280.2M, Control 186.2M). Bars, mean; error bars, SEM; points, individual replicates.

(j) P-site distribution of ribosome profiling samples from *Cnot7* KO, *Cnot8* KO and control gastruloids at 72 h shows enrichment in the coding sequence (CDS).

(k) Periodicity plot showing triplet periodicity in ribosome profiling samples from *Cnot7* KO, *Cnot8* KO and Control gastruloids.

(l) Scatter plot showing the $\log_2$ fold changes in transcript abundance and ribosome occupancy (translatome) of *Cnot7* knockout vs control gastruloids.

Supplementary Figure 7

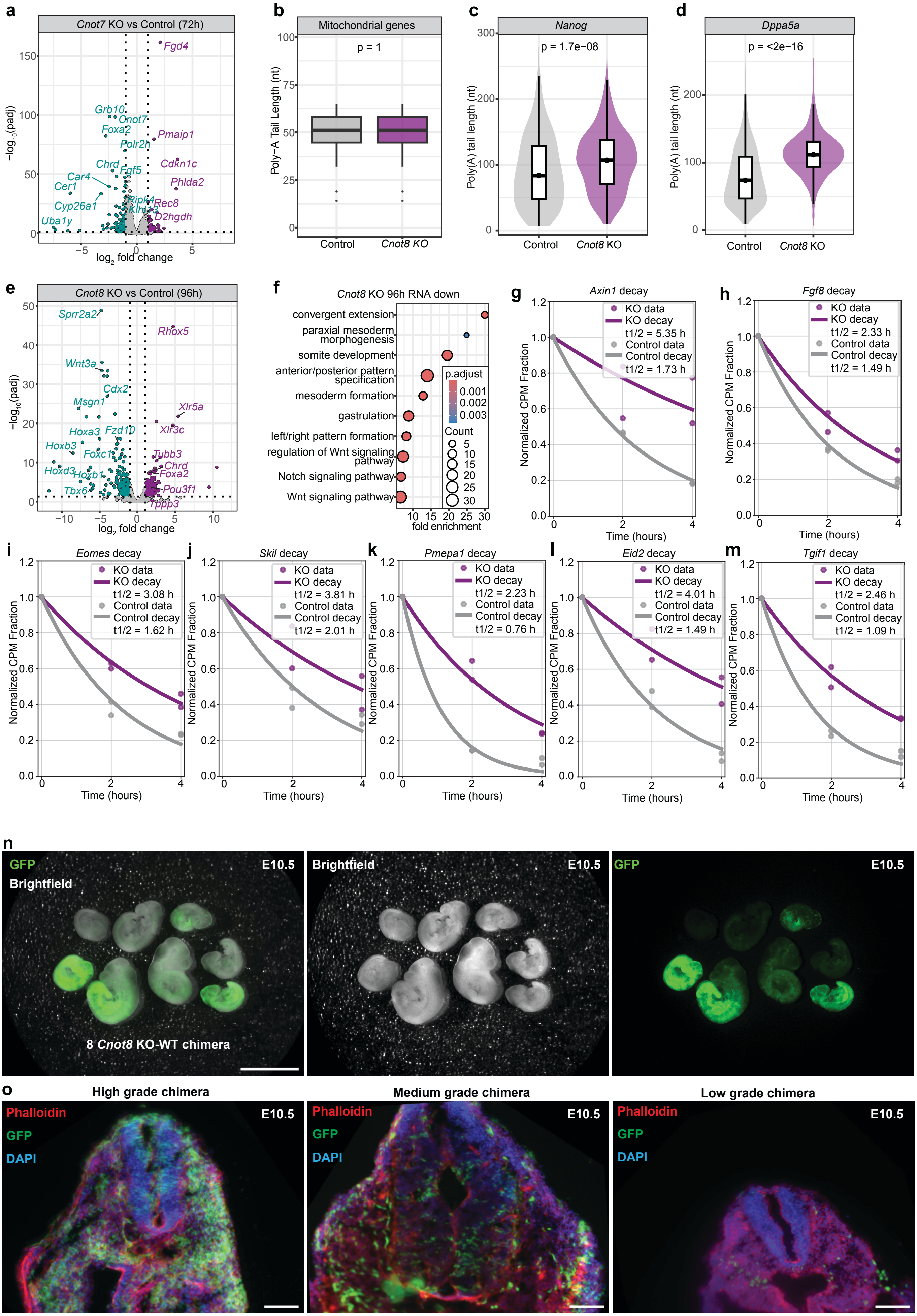

Supplementary Figure 7: Poly(A) length measurements and RNA degradation.

(a) Volcano plot displaying the top differentially expressed genes in 72 h *Cnot7* knockout gastruloids versus controls (replicates = 3).

(b) Box plot of median poly(A) tail lengths of mitochondrial genes.

(c-d) Box plot showing poly(A) tail lengths of *Nanog* (c) and *Dppa5a* (d).

(e) Volcano plot displaying the top differentially expressed genes in 96 h *Cnot8* knockout gastruloids versus controls (replicates =2).

(f) GO term enrichment of genes downregulated (FDR < 0.05, $\log_2$ fold change < -1) in 96 h *Cnot8* knockout gastruloids.

(g-m) Transcript decay plots of stabilized genes in *Cnot8* KO, including *Axin1* (g), *Fgf8* (h), *Eomes* (i), *Skil* (j), *Pmepa1* (k), *Eid2* (l) and *Tgif1* (m).

(n) Representative brightfield images and corresponding GFP overlays of E10.5 *Cnot8* knockout–wild-type mouse embryonic chimeras. Chimerism was assessed by GFP fluorescence, and most embryos were confirmed as *Cnot8* knockout–wild-type chimeras with varying degrees of contribution. Scale bar, 5 mm.

(o) Sections stained with Phalloidin (red) and GFP (green) in *Cnot8* knockout–wild-type mouse chimeras at E10.5. Scale bar, 100 μm.
