# Supplementary material for: Regulation of mRNA poly(A) tail length governs mammalian body plan formation in gastruloids
Source: Nat Commun. 2026 Aug 29;17:10390. doi: 10.1038/s41467-026-76897-3 (PMC13627704; doi:10.1038/s41467-026-76897-3)
Supplement: Supplementary file 4 [file 41467_2026_76897_MOESM2_ESM.pdf]

Description of Additional Supplementary Files

File name: Supplementary Data 1
Description: sgRNA library and MAGeCK results comparing library presentation in 120 h gastruloids with mESCs before doxycycline treatment as well as comparing individual cell types vs all other cell types.

File name: Supplementary Data 2
Description: Differential expression analysis of perturbated cells compared to non-binding control cells in 120h gastruloids.

File name: Supplementary Data 3
Description: Differential expression analysis and GO term analysis results from *Brachyury-P2A-mCherry*^{high} cells of 120h *Cnot7* KO and *Cnot8* KO gastruloids.

File name: Supplementary Data 4
Description: List of sgRNAs used for demultiplexing, Cluster Markers and differential expression of scRNA-seq data from 120 h *Cnot8* KO, *Cnot7* KO and Control gastruloids.

File name: Supplementary Data 5
Description: DeltaTE results showing changes in transcriptome, translatome and translational efficiency comparing 72 h vs 48 h, 96 h vs 72 h and 120 h vs 96 h wild type gastruloids.

File name: Supplementary Data 6
Description: DeltaTE results showing changes in transcriptome, translatome and translational efficiency in 72 h *Cnot7* KO vs Control and *Cnot8* KO vs Control gastruloids.

File name: Supplementary Data 7
Description: Median poly(A) lengths and $\log_2$ fold changes in 72 h and *Cnot8* KO vs Control gastruloids. Median half-lifes and half-life $\log_2$ fold changes in 96 h *Cnot8* KO vs Control gastruloids. Differential expression analysis of Cnot8 of 96 h *Cnot8* KO vs Control gastruloids.
